# Bone Phenotyping Approaches in Human, Mice and Zebrafish – Expert Overview of the EU Cost Action GEMSTONE (“GEnomics of MusculoSkeletal traits TranslatiOnal NEtwork”)

**DOI:** 10.3389/fendo.2021.720728

**Published:** 2021-12-01

**Authors:** Ines Foessl, J. H. Duncan Bassett, Åshild Bjørnerem, Björn Busse, Ângelo Calado, Pascale Chavassieux, Maria Christou, Eleni Douni, Imke A. K. Fiedler, João Eurico Fonseca, Eva Hassler, Wolfgang Högler, Erika Kague, David Karasik, Patricia Khashayar, Bente L. Langdahl, Victoria D. Leitch, Philippe Lopes, Georgios Markozannes, Fiona E. A. McGuigan, Carolina Medina-Gomez, Evangelia Ntzani, Ling Oei, Claes Ohlsson, Pawel Szulc, Jonathan H. Tobias, Katerina Trajanoska, Şansın Tuzun, Amina Valjevac, Bert van Rietbergen, Graham R. Williams, Tatjana Zekic, Fernando Rivadeneira, Barbara Obermayer-Pietsch

**Affiliations:** ^1^ Department of Internal Medicine, Division of Endocrinology and Diabetology, Endocrine Lab Platform, Medical University of Graz, Graz, Austria; ^2^ Molecular Endocrinology Laboratory, Department of Metabolism, Digestion and Reproduction, Imperial College London, London, United Kingdom; ^3^ Department of Clinical Medicine, UiT The Arctic University of Norway, Tromsø, Norway; ^4^ Norwegian Research Centre for Women’s Health, Oslo University Hospital, Oslo, Norway; ^5^ Department of Osteology and Biomechanics, University Medical Center, Hamburg-Eppendorf, Hamburg, Germany; ^6^ Instituto de Medicina Molecular João Lobo Antunes, Faculdade de Medicina, Universidade de Lisboa, Centro Académico de Medicina de Lisboa, Lisboa, Portugal; ^7^ INSERM UMR 1033, University of Lyon, Lyon, France; ^8^ Department of Hygiene and Epidemiology, Medical School, University of Ioannina, Ioannina, Greece; ^9^ Institute for Bioinnovation, Biomedical Sciences Research Center “Alexander Fleming”, Vari, Greece; ^10^ Department of Biotechnology, Agricultural University of Athens, Athens, Greece; ^11^ Rheumatology Department, Hospital de Santa Maria, Centro Hospitalar Universitário Lisboa Norte (CHULN), Lisbon Academic Medical Centre, Lisbon, Portugal; ^12^ Division of Neuroradiology, Vascular and Interventional Radiology, Department of Radiology, Medical University Graz, Graz, Austria; ^13^ Department of Paediatrics and Adolescent Medicine, Johannes Kepler University Linz, Linz, Austria; ^14^ The School of Physiology, Pharmacology and Neuroscience, Biomedical Sciences, University of Bristol, Bristol, United Kingdom; ^15^ Azrieli Faculty of Medicine, Bar-Ilan University, Ramat Gan, Israel; ^16^ Center for Microsystems Technology, Imec and Ghent University, Ghent, Belgium; ^17^ Department of Endocrinology and Internal Medicine, Aarhus University Hospital, Aarhus, Denmark; ^18^ Innovative Manufacturing Cooperative Research Centre, Royal Melbourne Institute of Technology, School of Engineering, Carlton, VIC, Australia; ^19^ Laboratoire de Biologie de l’Exercice pour la Performance et la Santé (LBEPS), Univ Evry, Université Paris Saclay, Evry, France; ^20^ Department of Clinical Sciences, Lund University, Malmö, Sweden; ^21^ Department of Internal Medicine, Erasmus MC Rotterdam, Rotterdam, Netherlands; ^22^ Department of Health Services, Policy and Practice, Center for Research Synthesis in Health, School of Public Health, Brown University, Providence, RI, United States; ^23^ Centre for Bone and Arthritis Research, Institute of Medicine, Sahlgrenska Academy at University of Gothenburg, Gothenburg, Sweden; ^24^ Department of Drug Treatment, Sahlgrenska University Hospital, Gothenburg, Sweden; ^25^ Musculoskeletal Research Unit, Translational Health Sciences, Bristol Medical School, University of Bristol, Bristol, United Kingdom; ^26^ MRC Integrative Epidemiology Unit, Bristol Medical School, Bristol, University of Bristol, Bristol, United Kingdom; ^27^ Physical Medicine & Rehabilitation Department, Cerrahpasa Medical Faculty, Istanbul University-Cerrahpaşa, Istanbul, Turkey; ^28^ Department of Human Physiology, School of Medicine, University of Sarajevo, Sarajevo, Bosnia and Herzegovina; ^29^ Department of Biomedical Engineering, Eindhoven University of Technology, Eindhoven, Netherlands; ^30^ Department of Rheumatology and Clinical Immunology, Faculty of Medicine, Clinical Hospital Center Rijeka, Rijeka, Croatia

**Keywords:** bone and skeletal diseases, phenotyping, imaging, animal models, GEMSTONE, COST

## Abstract

A synoptic overview of scientific methods applied in bone and associated research fields across species has yet to be published. Experts from the EU Cost Action GEMSTONE (“GEnomics of MusculoSkeletal Traits translational Network”) Working Group 2 present an overview of the routine techniques as well as clinical and research approaches employed to characterize bone phenotypes in humans and selected animal models (mice and zebrafish) of health and disease. The goal is consolidation of knowledge and a map for future research. This expert paper provides a comprehensive overview of state-of-the-art technologies to investigate bone properties in humans and animals – including their strengths and weaknesses. New research methodologies are outlined and future strategies are discussed to combine phenotypic with rapidly developing –omics data in order to advance musculoskeletal research and move towards “personalised medicine”.

**Graphical Abstract:**
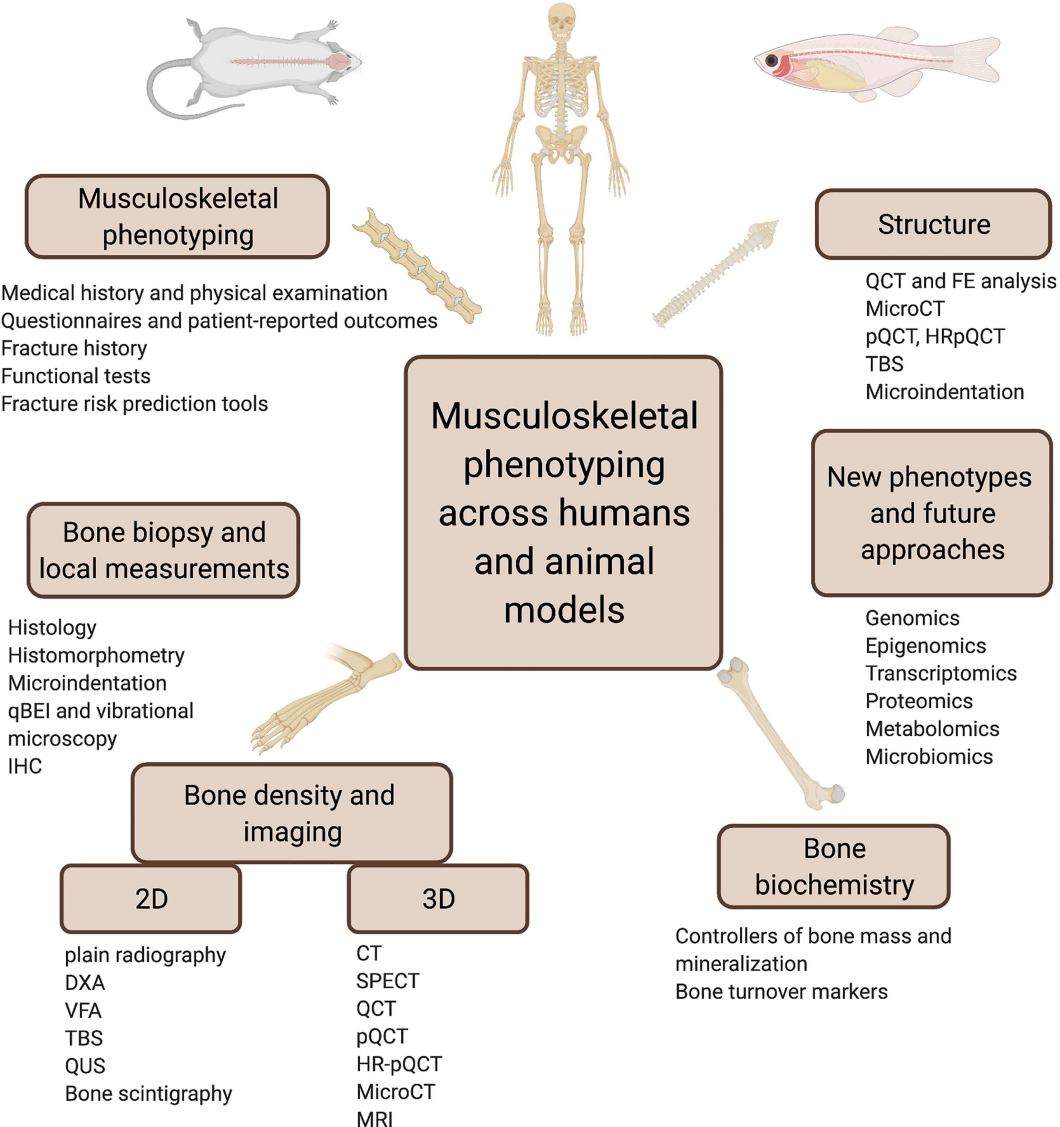


## Introduction

Bone metabolism and its regulation involves complex interactions and crosstalk across multiple tissues, physiological systems and pathways from fat and muscle to the immune system and gut-bone axis (1–3). With this knowledge and based on the recent advances in our understanding of genetics and genomics, this narrative overview of technological evidence intends a practical information for young researchers and/or scientists outside the respective bone areas to enable crosstalk between the disciplines.

The ultimate goal is to translate between clinical and preclinical research and aim for mutual interaction and development of future diagnostic and therapeutic approaches, drug development and risk assessment. This task is being undertaken by experts from the EU Cost Action GEMSTONE (“Genomics of MusculoSkeletal traits Translational Network”). One remit is to facilitate interaction between researchers in animal and human bone science and establish common phenotypic terminology across different spheres of expertise, thus enabling translational comparability of phenotypic signatures. The first step in this process, undertaken by GEMSTONE working group 2, Phenotyping, is to curate a comprehensive catalogue of bone phenotyping methods used within GEMSTONE in human and give a compressed overview on comparable methodology used in mice and zebrafish studies.

In this publication, we summarise the current state of the art, identify gaps in knowledge and suggest future directions/needs to be addressed. We provide insights in how the presented animal models can be used to model bone disease and complement human studies in order to advance bone phenotyping. Integrating the aims of this working group and the larger GEMSTONE action, we briefly outline how –omics technologies can contribute to the phenotypic dissection of skeletal traits. Finally, we offer our perspective on triangulation of the diagnostic evidence and lay out strengths and limitations of the respective techniques.

Discrepancies in the translation of clinical and preclinical research results are an important issue that complicates the understanding and progress in the care for patients with bone disease but also in associated disciplines in the bone field. Bone diseases are complex and multifactorial and require more than the just traditional methods to aim for new horizons with future diagnostic and therapeutic approaches.

Phenotyping and endophenotyping can be mechanistically oriented towards drug development, targeted treatment or prognostically oriented towards treatment stratification and treatment decisions. With this expert view on phenotyping methods across species, we aim for building bridges between animal and human bone science to establish common phenotypic terminology including growth-specific aspects, enabling translational comparability of phenotypic signatures for all researchers involved.

There are many open questions and unmet needs in the field of bone diseases in humans, such as the achievement of an optimal peak bone mass, robust evaluations of bone strength in clinical practice, including cross-validation between measurement methods and more holistic approaches in diagnosis as well as personalized, tailored treatment (4). An increased understanding of perspectives in animal models might help to solve a number of these open questions, as they are important issues for millions of people, e.g., diagnosis and treatment in children, adolescents or young adults, questions of the ideal use of current imaging techniques including new technologies to measure bone quality and strength, the interaction of epigenetic factors and the microbiome with bone quantities and qualities and future treatment options. Many new aspects might be answered by specific animal models, which are described in more detail.

Mice models are popular in studies of skeletal physiology due to their relatively low cost, high rates of reproduction, and ease of handling and care (5). They also provide the opportunity to collect phenotype data not available from humans, and to study the effect of single, specific interventions that are not possible in patients such as changes to diet, age, or genetics (6).

A number of features of bone biology are shared between the mouse and human skeleton. Like humans, mice experience age-related bone loss (5). They also undergo similar patterns of bone turnover and bone healing to humans (7, 8). However, there are differences that should be considered such as the lack of Haversian organisation and non-closure of growth plates at skeletal maturity (6).

Mice have a high homology to the human genome, making them suitable models for many human genetic disorders (6, 7). Manipulation of the mouse genome has allowed for the creation of models for numerous human musculoskeletal diseases. Transgenic and gene-targeted mice have allowed for studies of global overexpression or deletion of genes of interest for decades, but more recent technologies are making more specific genetic manipulation possible. The Cre-lox system applies for cell-specific and temporal deletion of target genes. In this system, LoxP sites are inserted on either side of the target gene or sequence, and when bred to a mouse expressing the Cre recombinase the relevant segment of DNA is excised in the desired cell type or developmental stage (9). CRISPR/Cas9 is the most recently developed technology and uses adapted bacterial proteins which cleave double stranded DNA at specific sites, offering a quick and accurate option for gene editing (10).

An additional model for bone research are small teleost fish such as zebrafish (*Danio rerio*) and medaka (*Oryzias latipes*). This model has been increasingly used to interrogate the biology of human skeletal conditions. Here, we will focus on zebrafish as an emerging and alternative model system used for the study of molecular mechanisms and gene function associated with human skeletal diseases. Zebrafish show conserved physiology compared to mammals and display advantages as animal model, such as the generation of a high number of embryos per cross (over 150), their rapid and transparent embryonic development that combined with the availability of a number of bone specific transgenic lines, allow *in vivo* cell trackability (11, 12). Moreover, genetic manipulation in zebrafish is relatively simple and highly efficient. Evaluation of the first bones in larval stages and adult whole skeleton can be performed in high-resolution with reasonably high throughput (13, 14). Zebrafish have been used for genetic and drug screening, and they pose an attractive model system to accelerate functional validation of human-omics findings.

Despite being evolutionarily more distant from humans than mice, zebrafish share key bone similarities, showing the same bone cell types (osteoblasts, osteocytes osteoclasts and chondrocytes) and types of ossification (intramembranous and endochondral) as those found in mammals, with the advantage that the first bones and cartilage are available for studies from the 3rd day of development (11). During ageing, zebrafish show bone macro and microstructure reminiscent of osteoporosis (15) and osteoarthritis (16). Furthermore, non-invasive bone fracture experiments in zebrafish allow investigation of bone healing and fracture repair (17, 18). At the molecular scale, zebrafish bone is reminiscent of mammalian bone up to the level of aligned mineralized collagen fibrils (19). Zebrafish also show some differences that should be considered. Unlike in humans, zebrafish bones do only show few bones with trabeculae, whereas long bones are absent. The bone marrow in zebrafish is fatty and does not harbour a site for haematopoiesis, but blood vessels invade the bone marrow similar to mammals (20). Zebrafish have growth plates, but the main source of longitudinal growth relies on cartilage proliferation and not from accumulation of hypertrophic chondrocytes, as only a small portion of chondrocytes become hypertrophic (21).

For further information and details, see also the GEMSTONE WG3 publication on “Gene & Therapeutic Discoveries in Bone Mass Disorders”.

Insights into mouse and zebrafish biology and pathophysiology will allow for a better understanding for human investigations and open clinical questions. There are substantial differences between the views of experts in human disease on various aspects of bone. Therefore, a translational approach for new research reducing the discrepancy between the animal and human models is highly warranted. Even in case, techniques cannot be directly compared, they may be tailored to specific research questions in the future.

Many links liaise this publication to those of Working Group 3 and 4 of the GEMSTONE COST Action with important details to many topics mentioned in this manuscript. This comprehensive overview allows us to better classify and detect bone diseases, predict disease progression using radiographic and clinical scores, clustering (identification of different groups/phenotypes of patients with bone diseases), pinpoint the most important characteristics that could affect disease progression and identify patients who will be rapid progressors for the development of late sequelae, e.g. multiple fractures. This paper aims to link the knowledge and understanding of different aspects of bone disease from various expert viewpoints, contributing to a solid basis for further and more effective cooperation between various specialities to enable a personalized care in this field in the future.

## 1 Musculoskeletal Phenotyping of Bone Conditions

**Figure 1:**
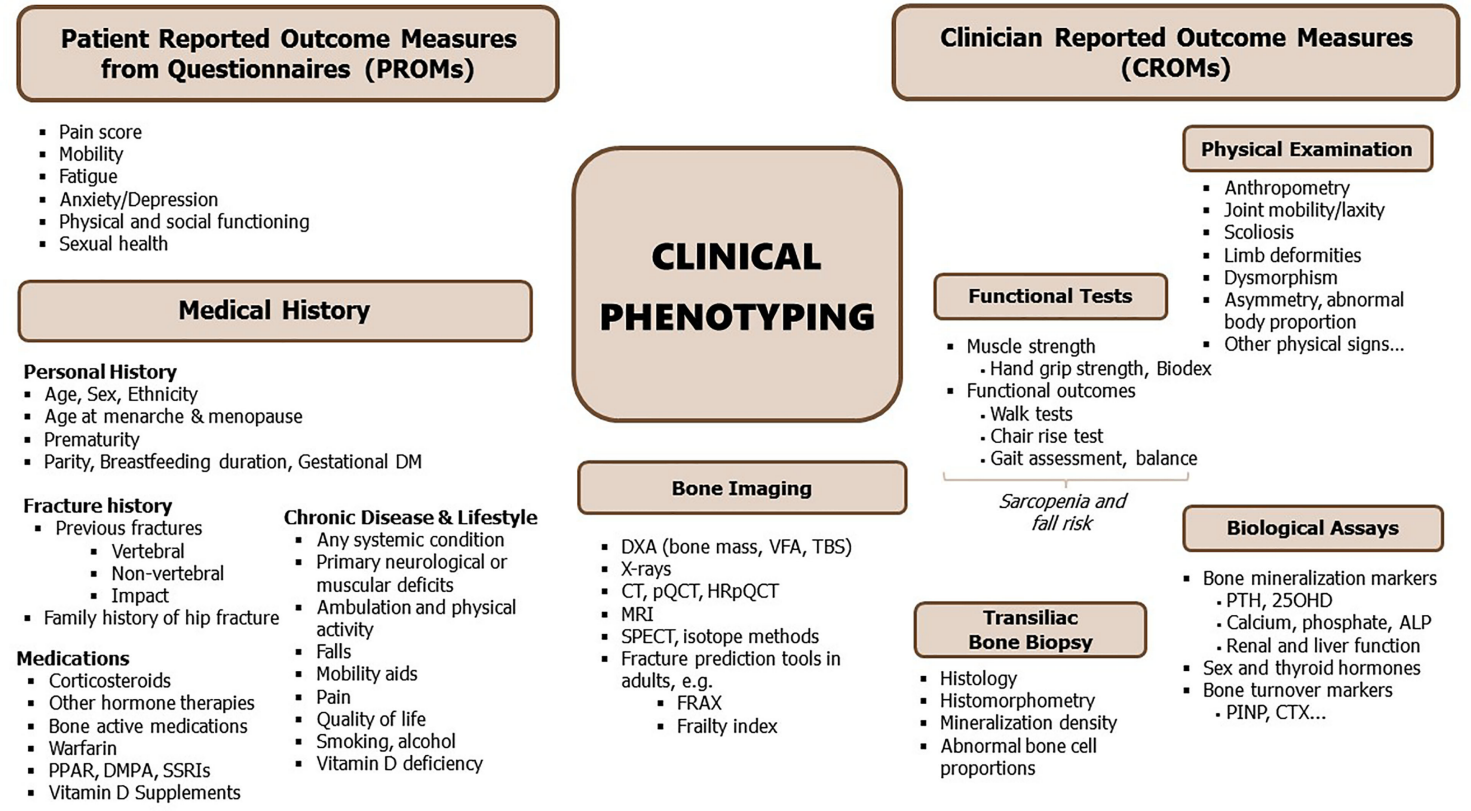
Synopsis of clinical phenotyping. ALP, alkaline phosphatase; Biodex, muscle strength by isodynamic dynamometer; CT, computed tomography; CTX, serum crosslaps; DM, diabetes mellitus; DXA, dual energy x-ray absorptiometry; FRAX, fracture risk assessment tool (https://www.sheffield.ac.uk/FRAX/); HGS, handgrip strength; HRpQCT, high-resolution peripheral quantitative CT; PINP, N-terminal propeptide of type I procollagen; PPAR, peroxisome proliferator-activated receptor agonists; pQCT, peripheral quantitative CT; QUS, quantitative bone ultrasound; SPECT, single photon emission computed tomography; SSRI, selective serotonin reuptake inhibitors; TBS, trabecular bone score; VFA, vertebral fracture assessment; biochemical parameters include 25OHD, 25-hydroxy-vitamin D; PTH, parathyroid hormone; [see also *Bone Turnover Markers (BTMs)*].

Musculoskeletal phenotyping is a broad and multi-faceted process that provides essential information for establishing a diagnosis of bone conditions, with or without bone fragility and muscle weakness. For all common or rare forms of musculoskeletal disorders, a comprehensive evaluation of clinical and functional aspects is required since fragility depends on much more than bone mineral density (BMD) alone (**Figure 1**).

### 1.1 Medical History and Physical Examination

A fragility fracture in children or adults is often the first sign of an underlying primary or secondary disease. A detailed medical history and thorough clinical examination can provide valuable insights into the overall state of musculoskeletal health. The content of the medical history depends on a patient’s age. In a child, family history of bone fragility, joint laxity or hearing loss gives essential clues towards the presence of genetic disorder, such as osteogenesis imperfecta. For humans of all ages, a history of back pain can relate to the presence of low-impact vertebral fractures, which may in turn increase the risk for future fractures. In addition, chronic or acute underlying conditions such as rheumatoid arthritis, diabetes, malabsorption, hypogonadism or premature menopause and stroke and neural damage may cause cytokine-, glucocorticoid- or immobility-induced metabolic disease that in turn can affect skeletal and muscular strength. The physical examination includes anthropometry, inspection of limbs and spine for deformities, assessing sclerae and teeth, palpation of spine and extremities along with observing the patient’s posture, limb length, muscle tone and mass, balance, joint mobility and gait. The spine is assessed for tenderness, and deformities (such as scoliosis, hyperkyphosis, or hyperlordosis). Decreased mobility and low lean mass predict low bone mass in humans according to the mechanostat theory (22). Sarcopenia, pain, presence of gait, balance and vision disturbances therefore provide important information on the risk of falling and future fractures. These parameters may be summarized in the concept of Patient Reported Outcome Measures from Questionnaires (PROMs) or Clinician Reported Outcome Measures (CROMs), respectively (see **Figure 1**) and may include a large number of additional terms, including psychological and social approaches. For more detailed phenotyping in genetic musculoskeletal diseases, see also, “Careful patient phenotyping is key to disease discovery” in the publication by GEMSTONE WG3.

**Limitations:** Taking a thorough medical history and assessing a deep clinical phenotype is time consuming and requires profound expertise of an experienced examiner. Studies may not even employ sufficient clinical phenotyping or time into this important investigation. An additional limiting factor may be a lack of knowledge and patients´ recall bias as well as the non-availability of x-rays or other clinical imaging for the clinician to confirm a patient’s fracture history and assess the radiological bone phenotype.

**Strengths:** Medical history and careful physical examination provide essential hints for the further diagnostic workup and avoid unnecessary or repetitive testing.

In mice models, detailed records and breeding charts should be kept for all mouse colonies, and these can and should be used as a proxy for medical history. These detailed colony records allow tracing of recurring skeletal problems or fractures. Physical examination is equally important in mice as in humans, and should include inspection of their condition, behaviour and environment. In regard to the skeleton and muscle tonus, this inspection should include examination of the incisors, gait abnormalities or lameness, and manually manipulating of the limbs (23).

In zebrafish, the skeleton can be regionalized into functional groups including the craniofacial skeleton and the vertebral column (together with the tail fin, being parts of the axial skeleton), and fins (pectoral, dorsal, anal fins). Since different genetic mutations often affect several skeletal compartments, it is common to perform skeletal phenotyping as a whole (13). Gross skeletal deformities such as scoliosis, hyperkyphosis, hyperlordosis, emaciation, as well as peculiar swimming patterns, are readily visible (16). Severe abnormal spinal curvature can be detected as the fish swim in the tank. It can be argued that “family history” is as relevant for the genetically-modified fish as for the mice of inbred lines; although the exact parents are usually not known for every specific fish, parental pairs usually come from well-documented strains/established mutants. Although there may be a lack of one-to-one relationship with the fish musculoskeletal phenotype during aging, the latter is measurable (24).

### 1.2 Questionnaires and Patient-Reported Outcome Measures

Patient-reported outcome measures (PROMs), which are collected using questionnaires are essential to comprehend the full extent of how musculoskeletal diseases influence the quality of life. In adults, questionnaires are often used to systematically collect information on self-reported socio-demography, medical conditions, family and fracture history, medication use, lifestyle such as dietary intake, smoking habits, alcohol consumption, physical activity and quality of life. Such information is important given that environmental factors, in combination with genetic susceptibility contribute to general frailty and risk for fracture.

In premenopausal women, there might be special attention to pregnancy and lactation based on hormonal changes and challenged calcium metabolism, due to the nutritional demands of the foetus and neonate (25–27). Female specific questions may address age at menarche, cycle abnormalities and conditions such as gestational diabetes (28) and preeclampsia, also for the child (29). In men, hypogonadism and other endocrine disturbances, but also exogenous toxins might be asked for. In children and adolescents, where heritable forms of osteoporosis are mostly diagnosed, questionnaires are not commonly used and emphasis is put on family history and physical examination.

A wide variety of patient-reported outcomes (pain, mobility, anxiety/depression, fatigue, peer relationship, physical function, sexual function) are available and can be collected as part of the European Registry for Rare Bone and Mineral conditions (https://eurr-bone.com). This EU initiative will provide extended phenotype information and increase knowledge about rare bone disorders.

Limitations: The quality of the patient interview is critical for the successful diagnostic support. Many PROMs questionnaires are validated and tested in a population-based setting and they are preferable over non-validated questionnaires. However, regular, systematic collection and assessment of PROMs in a clinical setting takes time and resources, which may not be available to doctors and patients alike. Self-reported information may not be well formulated to provide sufficient levels of detail, therefore, recall bias and other uncertainties have to be taken into consideration.

Strengths: PROMs questionnaires are widely available and at relatively low costs, and they are easy to administer, allow for repeated assessments and may use different formats (in person, postal, telephone, or electronic).

### 1.3 Fracture History

Family and personal fracture history are strong risk factors for fragility fractures in humans of all ages and can give hints to frailty in the elderly and genetic disorders and abnormalities in children. This reflects the genetic component of risk for fracture, particularly for hip fragility fracture (30).

Predicting the ‘first fracture’ is still challenging, since the majority who fracture do not have osteoporosis (31). A first fracture of any type doubles the risk of a new fracture (32). The timeframe for a new fracture is partially dependent on age and type; for a first fracture in young adulthood, the next may be 20 years ahead, but for an octogenarian, 2-3 years. Stress fractures – including both fatigue fractures (from abnormal, or repetitive loading on normal bone) and insufficiency fractures by normal loading on abnormal bone (33) - are important events in a patient’s history and should be an additional indication for a thorough clinical exploration for potential secondary causes (34). Fracture type and location are of particular relevance. Lower limb and vertebral fractures are typical for young children with osteogenesis imperfecta, vertebral fractures associated with back pain in acute lymphoblastic leukaemia and distal femur fractures in immobilized persons.

Limitations: Recall of elderly patients fracture history may be poor. Silent vertebral fractures can also come with little or no symptoms or they may be non-specific and therefore prone to be misinterpreted or overlooked (35).

Strengths: Information is easily ascertained in a healthcare setting and the well-established link with family history and previous fracture should be sufficient to merit bone characterization and potential pharmacological and/or non-pharmacological musculoskeletal management *via* a Fracture Liaison Service (FLS) (36).

In mouse models, most studies describe changes in material properties and histology at a certain timepoint (37). Therefore, fracture history for an individual mouse will not be evaluated. However, in the context of mouse strains, probability and time until a fragility fracture occurs might provide important information.

In the zebrafish skeleton, ribs and fins should be given attention when analysing fractures. Zebrafish models for osteogenesis imperfecta e.g. show recurrent fractures in the ribs and fins (38–40). Rib fractures can be evaluated through life using radiographs, as well as analysing the fins under a transmitted light microscope. Fracture recurrence can be annotated longitudinally.

### 1.4 Functional Tests

The functional assessment of an increased risk of fall *via* the muscle-bone unit involves evaluation of a) muscle force using tools such as dynamometer, leg press and chest press; and b) physical performance using tools such as 30sec or 6min walk test (gait speed test), chair rise test, short physical performance battery (SPPB) and timed-up-and-go test (TUG) (reviewed in detail elsewhere) (41).

In children, the chair rise test, the 30sec or 6min walk test or the BOT™-2 (Bruininks-Oseretsky-Test of Motor Proficiency, second edition) test are commonly used.

Limitations: The results of the functional tests are largely influenced by the presence of chronic diseases and the patient’s cooperation as well as trained health care personnel.

Strengths: The dynamometer and the gait speed tests can be of greatest utility given the fact they can be used in research settings, in specialist clinical settings and in primary care settings at very low expenditures. These techniques provide valuable information on muscle mass and function, important determinants of falls and fragility fracture risk.

In mice, gait analysis can be used to detect abnormalities in speed, stride length, and limb-force profile (42). This technique has been used to measure altered stride length, velocity and limb angle after fracture fixation in mice (43). For muscle mass and strength assessment, multiple methods such as grip strength test (44), wire hang test (45), treadmill test (46), vertical pole test (47)and swimming endurance (48). Additionally, invasive methods include *in vitro* and *in situ* muscle force measurement (49).

Adult swimming behaviour analysis in the fish provides information on how the skeletal system is functioning as a whole (bone and muscles), with potential measurements of angle achieved during the swim, velocity achieved after tail propulsion, as well the time that it takes for exhaustion and induction of fractures (50).

### 1.5 Fracture Risk Prediction Tools

For a potential prediction of future fragility fractures, information gathered from the above described tools can be used with risk calculators that combine several risk factors, with or without BMD testing, e.g. the Fracture Risk Assessment Tool (FRAX^®^) algorithm^1^, the Garvan Fracture Risk Calculator^2^ and QFracture^®^ ^3^. These tools provide a valuable risk stratification for the screening and management of osteoporotic patients (51). As an example, the FRAX^®^-based community screening in the elderly is increasingly used to provide individualized 10-year probability estimates of hip and major osteoporotic fractures (52). However, to date, there is no consensus on the discriminative ability of these tools to predict fragility fracture risk, except FRAX^®^ with BMD, Garvan with BMD and QFracture^®^ (53). Furthermore, the holistic approach of data collection together with physical and clinical measurements could help the construction of frailty index scores (54, 55) to identify subjects at higher risk of fragility fractures (56), and mortality (57).

In children, such prediction programs have not been developed since the underlying conditions vary in nature; osteoporosis can be transient (e.g., acute leukaemia) or permanent (genetic). For example, vertebral fractures may spontaneously reshape in a leukemic child if the remaining growth potential suffices but this would be highly unlikely in a child with osteogenesis imperfecta (58).

Limitations: Some tools might be less representative for a number of important factors, such as probably an individual bone turnover. A lack of medical history data or the number of prior fractures might result in over- or underestimating a person’s personal risk.

Strengths: Community screening is more easily feasible and patients may be more adherent to bone-active treatment options in view of numeral risk estimation.

In a quadrupedal mouse model, studying bipedal fracture risk and the link between muscle mass/strength and falls is difficult. However genetically modified models, as well as induced fracture models, allow for the study of changes in motion and function of the muscle bone unit which may provide insight into human cases.

Zebrafish fractures, their numbers and recurrence can be easily evaluated *in vivo* and longitudinally. As in mammals, fractures that happen early in life would indicate higher risks of fracture recurrence in zebrafish. Nevertheless, there are no estimates available, yet, for fracture risk predictions in zebrafish.

## 2 Bone Density and Imaging - 2D

**Figure 2:**
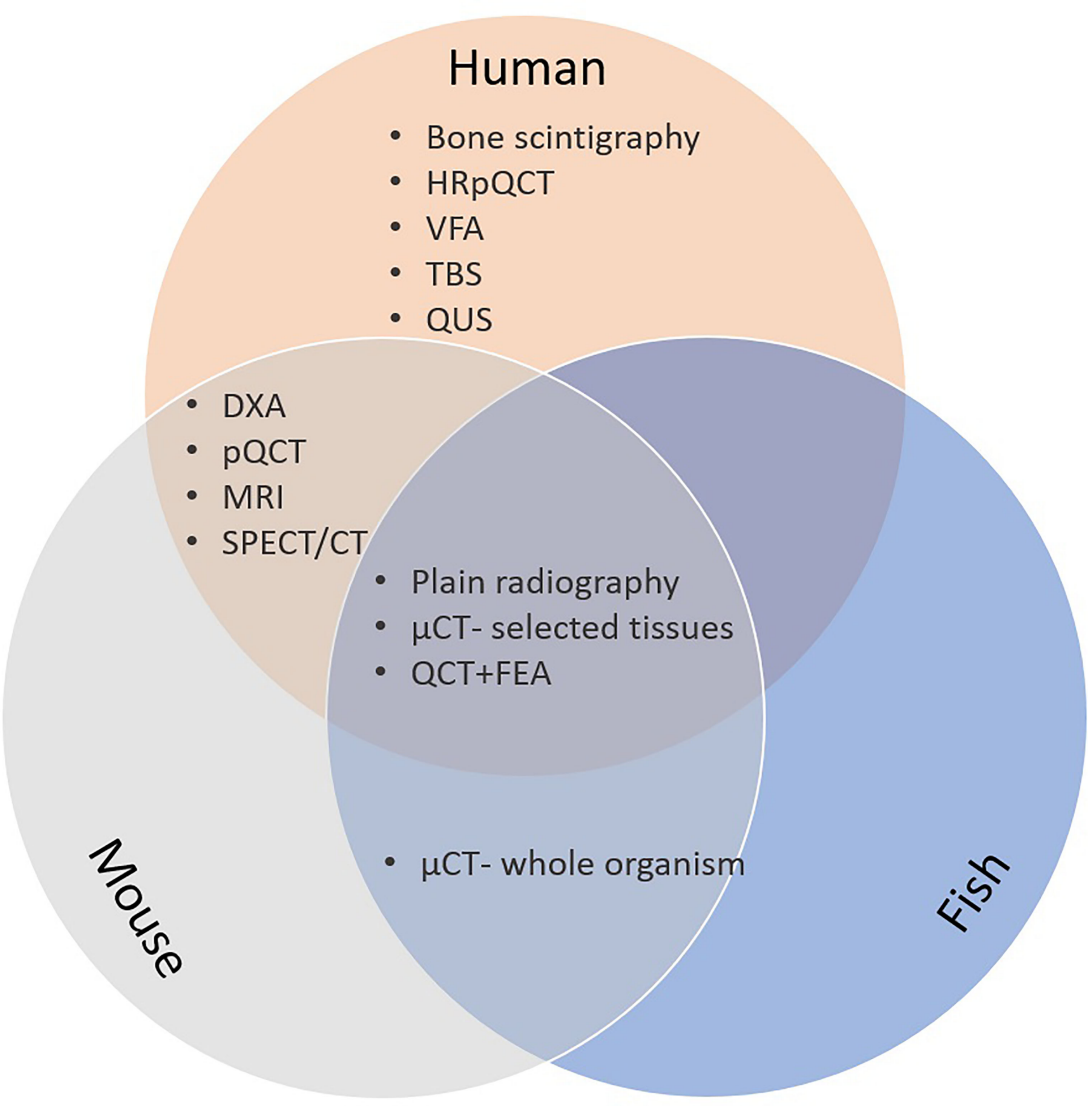
Bone imaging techniques in humans; mice and fish. CT, computed tomography; µCT, microCT; DXA, dual energy x-ray absorptiometry; FE, finite element analysis; HRpQCT, high resolution peripheral quantitative computed tomography; MRI, magnetic resonance imaging; pQCT, peripheral quantitative CT; QCT, quantitative CT; QUS, quantitative bone ultrasound; SPECT, single-photon emission computed tomography.

Many different imaging modalities have been used to quantify bone density, strength, fracture risk and remodelling (**Table 1**). Some of these methods are specific for the human, but many can be used as well (in modified form) for animals (**Figure 2**). Essentially, imaging methods can be 2D (slices or projections) or 3D. In this section we focus on the 2D imaging methods while the next section deals with 3D methods.

**Table 1:** Comparison of 2D imaging and bone density techniques between species.

Imaging technique	Human		Mouse/rat models		Zebrafish models	
	Strengths	Limitations	Strengths	Limitations	Strengths	Limitations
Plain radiographs	Widely available	2D analyses	availability	2D image	Longitudinal skeletal assessment	Detailed aspects of bone morphology and density are not captured due to the imaging resolution, overlay with soft tissues, and small bones in zebrafish
	Additional density estimation in development	Potential superposition		Poor or inconsistent positioning of the animal or bone.	Relative bone density estimation	
	Low cost				Low cost and rapid imaging for high-throughput screenings	
	Moderate radiation dose				Full fish recovery after imaging	
DXA	Low radiation	Artefacts from bone (fractures), osteophytes, vascular calcifications and other superpositions	Most suitable method for BMD measurement in small animals	General anaesthesia needed	N.A.	N.A.
	Fast and highly reproducible measurements	2D information only		Poor edge detection and accuracy for very small animals (<50 gr)		
	Widely available and full automatization	No differentiation of trabecular vs cortical compartments		Accurate positioning of the animals and placement of the region of interest can be challenging		
	WHO/ISCD definition for osteoporosis/osteopenia Individual longitudinal monitoring possible	No correction for bone size or skeletal maturity (e.g. in children)		Measurements affected by size and weight of the animal		
TBS	Non-invasive	No direct relation to fracture risk published	N.A.	N.A.	N.A.	N.A.
	Tool for trabecular bone structure	Improvement of risk prediction *via* FRAX				
	Discrimination in secondary osteoporosis e.g. in diabetes mellitus	Potential artefacts				
VFA	Information on vertebral fractures	Lateral positioning of patient sometimes difficult	N.A.	N.A.	N.A.	N.A.
	Low radiation exposure					
QUS	Transportable	No WHO definitions of osteoporosis/osteopenia	N.A.	N.A.	N.A.	N.A.
	Quick	Many different devices – no standardization				
	Non-invasive	Individual monitoring difficult				
	radiation free	No direct translation to bone structure				
	Inexpensive					
	It can be used apart from specialised centres					
Bone scintigraphy	Widely available	Potential false positive results	Mainly use of SPECT (see *Single-Photon Emission Computed Tomography (SPECT)*)	Mainly use of SPECT (see *Single-Photon Emission Computed Tomography (SPECT)*)	N.A.	N.A.
		Inferior to SPECT in 3D questions				

DXA, dual energy x-ray absorptiometry; FRAX, fracture risk assessment tool; QUS, quantitative ultrasound; SPECT, Single-photon emission computed tomography; TBS, trabecular bone score; VFA, vertebral fracture assessment.

N.A., not applicable.

Different options are available for 2D bone assessment based on imaging in humans, which include plain radiography, bone densitometry by dual energy x-ray absorptiometry (DXA), bone scintigraphy, as well as vertebral fracture assessment (VFA) and trabecular bone score (TBS) based on lumbar spine DXA. The different imaging modalities have specificities in their local availabilities, as well as varying advantages and disadvantages depending on the technology, like radiation exposure, spatial resolution and the information that can be obtained.

### 2.1 Plain Radiography

Conventional and digital x-rays are widely available and are frequently used as the first-line overview for imaging almost all pathological changes in the bone e.g. to assess bone structure and morphology in case of a suspected vertebral fracture. The main feature of osteoporosis in radiographs is increased radiolucency of the trabecular bone and cortical thinning, though this is mostly subjective and with low specificity, found at advanced stages of osteoporosis when bone mass is substantially reduced or bone mass accrual was insufficient as in osteogenesis imperfecta (OI) (59), and shows other mechanical or inflammatory changes of the vertebrae.

The Genant classification of vertebral fractures has been implemented using a semi quantitative technique (60) in five subtypes (OF1-5) (61) based on lateral vertebral imaging with a relatively low interobserver variation [see *Vertebral Fracture Assessment* (*VFA*)].

Limitations: The biggest disadvantage of plain radiography for assessing changes in the bone structure is the 2D nature, resulting in superposition of three-dimensional structures consisting of soft tissue and hard tissue onto a 2D plane. Thus, the interpretation can be difficult due to the superposition of shadows (62, 63). Another drawback is the limited resolution (order 200 microns) and the inability to discriminate between low bone mass and mineralization defects. As for all the techniques involving x-rays, there should be careful consideration between examination outcome and radiation dosage.

Strengths: Nevertheless, plain radiographs are widely available and some additional software techniques for bone density estimation from radiographs are under development. Generally, radiography is the first assessment due to the wide availability of the equipment, and the low cost (64). Radiographs may also provide an initial differential diagnosis covering also scoliosis assessment and other diseases of the spine presenting with back pain.

In mouse, 2D radiography is a highly sensitive method to study bone properties. The x-ray microradiography imaging is a useful tool for phenotyping. With this technique an X-ray tube with a small spot size (around 10 microns) is used that enables magnified projections of bone details. It can be used to assess changes in bone size and cortical thickness, and if used with appropriate standards, it can also provide a quantitative measure of mineral content (65). It also has the ability to detect cortical thinning and bone loss as seen in humans suffering osteoporosis (66). Lateral x-ray imaging has been applied in high-throughput format to identify bones with altered length and mineral content (66). It has the benefit of being fast and non-destructive, but drawbacks include that it only provides a two-dimensional image and may be affected by poor or inconsistent positioning of the animal or bone.

In zebrafish, radiographs are useful for rapid evaluation of skeletal deformities and bone density. As an example for the power of the technique, Fisher et al. have identified the zebrafish mutant Chihuahua (chi) (mutation in the α1 chain of collagen type I) through a zebrafish forward genetic screening in which the authors leveraged from radiographies to screen a high number of adult zebrafish for skeletal abnormalities (67). Radiographs allow longitudinal studies of the zebrafish spine. Imaging takes a few seconds, allowing anesthetized zebrafish to be imaged without water, and followed by full recovery. However, due to the small size of the zebrafish bones, many aspects of bone morphology, microarchitecture, and mineralization, are limited in radiographic analysis, while µCT captures all these metrics.

### 2.2 Dual Energy X-Ray Absorptiometry (DXA)

DXA provides a two-dimensional (2D) representation of bone, but also information about body composition including lean and fat mass. Measurement sites include the lumbar spine, hip, radius and whole body. Though anterior-posterior scans are generally obtained, lateral spine scanning is also performed to assess vertebral morphology and fractures (see *Vertebral Fracture Assessment* (*VFA*). The DXA image comprises a series of pixels containing information about mineral content. Total mineral content within a region of interest is defined, from which bone mineral density [BMD, in g/cm^2^, also often noted as areal BMD (aBMD)] is obtained after dividing bone mass by bone area. aBMD measured by DXA predicts fracture risk in adults (68), for which this method is widely used for clinical and research purposes. In clinical settings, aBMD is compared to a young reference cohort of the same ethnic background and sex, generating a T-score. The International Society of Clinical densitometry (ISCD) defines osteoporosis in adults as a T score ≤2.5, representing 2.5 standard deviations below the young reference mean value (69). Another score reported from DXA measurements is the Z-score. This score quantifies the number of SDs above/below the mean value of an age and sex matched population. This score is not used for the diagnosis of osteoporosis but provides information about an individual’s fracture risk compared to peers (70). Volumetric bone mineral density (mass per volume, (vBMD)) also noted as bone mineral apparent density (BMAD) is strongly correlated with bone strength in experimental studies (71). Although DXA just provides an ‘areal’ density, this remains the most common technique of assessing bone strength clinically. BMD thresholds also contribute, but less than in adults, to the diagnosis of osteoporosis in children (58, 72). In children, aBMD data require adjustment for body size to avoid misinterpretation from size artefacts, by using lumbar spine BMAD and total body less head (73). The ISCD definitions of osteoporosis in children are mainly based on the presence of fractures (74). Hip structural analysis (HSA) (75, 76), has been developed to derive other parameters related to bone strength, for example by calculating femoral neck width (77). Finite element analysis has also been applied to hip DXA images, which may provide additional information on fracture risk (78). In addition, current DXA devices enable specific morphological features to be assessed such as vertebral fractures and osteophytes. Due to the strong relationships with fat and particularly lean mass, DXA scans provide regional body composition measures which are particularly useful in evaluating android and gynoid fat distribution (79). In fact, DXA is considered the gold standard method for body composition assessment in clinical practice due to its advantages of high accuracy and precision, low cost, low radiation dose and short scan time. It has a variety of clinical applications, such as diagnosis and follow-up of lipodystrophy and sarcopenia, as well as being widely used in research studies of body composition. Further technologies based on DXA are analyses of fractures by Vertebral Fracture Assessment (VFA), and the trabecular bone score (TBS) described in section *Vertebral Fracture Assessment (VFA)* and *Trabecular Bone Score (TBS)*.

Limitations: BMD can be artificially elevated by collapsed vertebrae or mineral deposits at sites that do not contribute to bone strength, such as osteophytes and aortic calcification. In addition, due to its 2D nature, DXA is unable to capture the complex 3D morphological characteristics of skeletal elements. For instance, the trabecular vs cortical compartments cannot be differentiated and DXA gives no information on the bone microarchitecture (80). Moreover, DXA measurements are not corrected for skeletal size, hence DXA underestimates BMD in humans and animals with short stature, and overestimates BMD in those with tall stature. Such artefacts are not generally corrected for in adult medicine and in many animal studies. In children or growing animals alike, interpretation of DXA results requires adjustment not just for age and sex but also for body or bone size, and skeletal maturity (bone age or pubertal status).

Strengths: DXA scans use very low radiation doses and a fast-scanning mode, making this method suitable for research as well as clinical use. Derivation of commonly used measures such as BMD is fully automated and highly reproducible, enabling small changes to be detected in longitudinal studies.

In mice, as in humans, DXA is the most commonly used method for measuring BMD (81). DXA has been demonstrated to be accurate and precise in measuring total bone and bone mineral content in mice (82). It has been used to characterise the bone loss in multiple models of post-menopausal osteoporosis in mice (83). Benefits in animal characterization include the ability for live imaging, its low cost, relatively fast speed, and low-radiation emission. Limitations of the application of DXA in animal models are the low resolution of the technique, and the need for correct (sometimes repeated) positioning.

For zebrafish, techniques of bone density measurements are reflected in section *MicroCT (µCT)*.

### 2.3 Vertebral Fracture Assessment (VFA)

VFA uses lateral DXA imaging of the thoracic and lumbar spine for the presence of vertebral fractures (VF). Images can be obtained at the same time as areal (aBMD) measurement. The radiation exposure is lower than in plain radiographs of the spine (84). According to the ISCD, Genant’s semi-quantitative fracture assessment is the method of reference for the diagnosis of VF on VFA or other lateral spine imaging (74, 85–87). 

Limitations: In some devices, the analysis requires a lateral positioning of the patient, which is sometimes not feasible. This limitation can be solved by the use of a “C-arm”, allowing supine lateral spine imaging. The upper thoracic vertebrae (Th4 to Th7) might be poorly visible.

Strengths: The low radiation exposure of VFA is an advantage in all, but especially in paediatric patients (according to the latest ISCD paediatric recommendations (74, 88), as well as the combination of both aBMD and VFA in one session.

In mouse, VFA is not used in the mouse skeletal phenotyping.

In zebrafish, vertebral fractures can be assessed longitudinally through radiographs and post-mortem through µCT and whole mount staining (Alizarin Red S staining). Although vertebral fractures in zebrafish are not commonly observed, compressive forces applied *ex vivo*, anteroposterior at the vertebral column and visualized using µCT, have demonstrated points of stress in the vertebrae where it is subjected to fracture in zebrafish (89). Recently, a non-invasive method to induce fractures in zebrafish has been established. By using physical pressure applied to the fin rays of the anaesthetized fish, one can easily cause fin fractures (90). This allows the assessment and the study of fractures from the initial moment that they happened.

### 2.4 Trabecular Bone Score (TBS)

Trabecular bone score (TBS) is a texture-based index that provides an indirect assessment of trabecular bone microarchitecture. It is calculated based on the pixel gray-level variations in lumbar spine DXA images (78, 91). While this index is increasingly used in adult human patients, there are no animal studies to date, except *ex vivo* comparisons with porcine vertebrae (92). TBS provides additional information on fracture risk and is mainly used in secondary osteoporosis, e.g. in diabetes (93) and ankylosing spondylitis (94). However, the proportion of risk prediction in a more general osteoporosis approach warrants further studies, and also depends on the software used (TBS iNsight^®^, Version 4.0) with a 54% (OR 1.54; 95% CI, 1.18 to 2.00) increase of having a major osteoporotic fracture (MOF) for each standard deviation decline in TBSv4.0 values (95). Chronological age and TBS are related; significant age-related changes seem to occur with a turning point to higher TBS values at age 8 in girls and age 10 in boys (96). The use of the TBS has not yet been sufficiently explored or recommended for clinical use in children, (see the current ISCD position at iscd.org/learn/official-positions, last access Dec 2020). Assessment of TBS at other bone sites than the lumbar spine might be an interesting development (97), also in view of comparisons with animal measurements and high-resolution (peripheral) quantitative computed tomography HR(p)QCT or bone biopsies including state-of-the-art histomorphometry.

Limitations: TBS is used as an add-on tool to DXA-scans; patterns for specific osteoporosis risk prediction are warranted. An independent contribution of TBS to fracture prediction seems to be small (98), and potential artefacts can be due to collapsed vertebrae.

Strengths: The respective software is a widely used non-invasive tool for indirect assessment of trabecular bone structure based on already existing compatible DXA scans. TBS might allow for a discrimination of patients at risk, e.g. in secondary osteoporosis, where DXA alone does not. The inclusion of TBS into the Fracture Risk Assessment Tool (FRAX^®^) may improve the fracture prediction beyond FRAX^®^ without TBS.

In mice and zebrafish, TBS or similar scores are not currently used.

### 2.5 Quantitative Ultrasound (QUS)

QUS provides a measure of bone quality and quantity (99). Broadband ultrasound attenuation (BUA, dB/MHz) reflects anisotropic characteristics of trabecular bone, and speed of sound (SOS, m/s) refers to the division of sound waves by the length of the bone and transmission time. Some devices combine BUA and SOS values to provide a quantitative ultrasound index (QUI) or stiffness index (STI). Though providing a distinct measure to DXA-evaluated BMD, QUS and DXA have similar predictive value for hip fracture risk in elderly populations (100). QUS is used to assess easily accessible bones like the calcaneus, which is the most widely used measurement site, and patella, tibia, metatarsal bone at weight-bearing sites, as well as phalanges and radius (101). Clinically, QUS is used as a screening tool for osteoporosis. QUS has also provided major insights for genetic discovery, through its incorporation in UK Biobank, based on estimated bone mineral density (eBMD, g/cm^2^) derived as a linear combination of SOS and BUA (102). The device is not recommended for routine use in children and adolescents.

Fracture sonography is a special field of application of medical ultrasound diagnostics (sonography) for the detection of bone fractures. In addition, there are other applications of bone sonography, such as osteoporosis diagnostics and for the representation of callus. In patients younger than 12, proximal humerus or clavicle fractures can be visualized by ultrasound due to the changes at the bone surface (103).

Limitations: As WHO definitions of osteoporosis and osteopenia require DXA measurements (104), confirmation of QUS findings by DXA measurement is needed. A lack of standardization hampers result comparison, in view of the many different QUS devices available and of the influence of environmental conditions (temperature etc.). Furthermore, in contrast to DXA, the precision of QUS is insufficient for monitoring individual patients (101). QUS cannot assess bone structure.

Strengths: Advantages of QUS include being transportable, quick, non-invasive, radiation free, inexpensive, and useful for large population screening studies even apart from health care centres.

In mice and zebrafish QUS is not used.

### 2.6 Bone Scintigraphy

Bone scintigraphy detects an increase in osteoblastic activity or vascularization, which may be associated with osteoporotic fracture or localized bone lesions. Radionuclides such as technetium-99m [99m Tc], often linked to a bone-avid tracer molecule such as bisphosphonates, e.g. 99mTc-methylene diphosphonate (MDP) emit gamma-radiation in proportion to their attachment to a target structure. This technology may supplement radiographs with additional information about recent/old fractures or may identify radiographically occult injuries and differential diagnoses such as metastatic disease (63).

Limitations: Bone scans are a sensitive technique, but may produce false positive results and cannot determine the extension of a fracture, whereas SPECT is superior in the detection of vertebral fractures (see section *CT-Based Techniques*).

Strengths: Bone scintigraphy is widely available in specialized nuclear medicine departments and may be used to address clinical questions not only for oncological diagnosis. It can discriminate recent from healed spinal fractures and demonstrate evidence for radiographically difficult to assess fractures, e.g. atypical femoral fractures (105). Furthermore, positive tracer uptake is reported in areas that subsequently develop osteonecrosis of the jaw (ONJ) (106).

In mice, radioactive methods are widely used, but mainly in context with SPECT (see section *CT-Based Techniques*).

In zebrafish, scintigraphy is nor used mainly due to the water-based environment.

## 3 Bone Density and Imaging - 3D

Development of three-dimensional (3D) methods for bone imaging allowed for new approaches in bone phenotyping, such as computed tomography (CT), single-photon emission computed tomography (SPECT) and magnetic resonance imaging (MRI) (**Table 2**). Implementation in clinical use and research protocols depend on local availability and technical knowledge. For scientific purposes, international cooperation of researchers might be an additional benefit by bringing bone scientists together.

**Table 2:** Comparison of 3D imaging techniques between species.

Imaging technique	Human		Mouse/rat models		Zebrafish models	
	Strengths	Limitations	Strengths	Limitations	Strengths	Limitations
CT	Widely available	High radiation exposure	See µCT below	See µCT below	N.A	Low resolution
	Morphological use	No direct comparison to 2D methods (e.g. DXA)				
	Concomitant differential diagnosis					
SPECT/CT	Correlation of skeletal standardized uptake values (SUVs) and BMD possible	Not useful in children due to radiation and inflammation concerns radiation exposure	Good spatial resolution	Radiation	N.A.	N.A.
			Useful for bone growth and repair			
			Non-invasive and longitudinal tracking of changes			
QCT	Volumetric bone density information	Considerable costs	See µCT below	See µCT below	See µCT below	See µCT below
	Can be used for continuum FE models	Higher radiation dosage				
		Long operational time				
pQCT	Evaluation of cortical and trabecular bone density, structure and strength	Thresholding and difficulties with standardisation at distal sites	See µCT below	See µCT below	See µCT below	See µCT below
	Relatively low radiation dose	Size artefacts by partial volume effect				
		Needs adjustment for bone length				
HR-pQCT	Only existing non-invasive imaging method obtaining bone microarchitecture	Only for distal extremities	See µCT below	See µCT below	See µCT below	See µCT below
	Fast and safe	Movement artefacts				
	Low radiation dose 3 µSv/scan	Manual analysis required due to potential inaccurate estimates				
	Good reproducibility No side effects					
MicroCT (µCT)	In bone specimen, fast and non-destructive assessment	*Ex vivo* use in humans only	Most suitable method for skeletal measurement	Time-consuming	Most suitable method for skeletal measurements as well as assessment of individual bone morphologies	High radiation allows only *ex vivo* bone assessment
	Excellent reproducibility and accuracy	Lack of specificity for soft tissues		Stabilization required		
				Radiation exposure		
MRI	No radiation exposure	No direct comparison to 2D methods (DXA)	Longitudinal assessment	Long scanning time	Bones and muscles can be visualized	Aquatic flow cell system is needed for *in vivo* scanning
	Widely available			Low resolution due to small sample		Low resolution
	Well-defined morphological tools					Difficult in use

CT, computed tomography; µCT, microCT; FE, finite element analysis; HRpQCT, high resolution peripheral computed tomography; MRI, magnetic resonance imaging; pQCT, peripheral quantitative CT; QCT, quantitative CT; SPECT, Single-photon emission computed tomography.

N.A., not applicable.

### 3.1 CT-Based Techniques

#### 3.1.1 Computed Tomography (CT)

Computed tomography is a sectional imaging method that allows a representation of soft tissues, bones and vessels. Thanks to the spiral technology (except QCT), clinical CTs produce small isotropic voxels, which enables a high spatial resolution in any spatial direction. The multiplanar slicing also allows sagittal and coronal representations of high quality and 3D visualization provides structural and morphological information. The x-ray-based imaging technique is widely used for characterization of degenerative changes, vascular and soft tissue calcifications (84). The voxel size varies according to the method, e.g., 250 - 1000 µm for clinical whole-body CTs, 50 - 80 μm for HR-pQCT [see *High Resolution Peripheral Quantitative Computed Tomography (HR-pQCT)*] and 10 - 30 µm for microCT (see *MicroCT*, used for instance for microscopic bone structure analysis on bone biopsies) (107).

Quantitative CT enables the measurement of volumetric BMD (vBMD) at the spine and any bone, and allows separate evaluation of cortical and trabecular bone. Further details are available in *Quantitative CT*.

Limitations: CT scans involve high radiation exposure. A direct comparison with DXA is not possible.

Strengths: CT scans are widely available and can be used for the characterization of morphological changes and differential diagnoses.

In mice and zebrafish, clinical CT scanners are not used due to the low resolution (See µCT section *MicroCT*).

#### 3.1.2 Single-Photon Emission Computed Tomography (SPECT)

Non-quantitative bone scintigraphy using 99mTc-MDP may be combined with CTs for Single-photon emission computed tomography SPECT)/CTs. Standardized uptake volume (SUV) is also used for bone metabolism measurements. The range of SUV in normal lumbar spine is roughly coherent with 18F-fluoride in positron emission tomography (PET). In addition, it correlates positively with Hounsfield units (HU) of the lumbar spine and negatively with age (108). As a fusion method SPECT/CT has been shown to be superior to SPECT alone in the identification of vertebral lesions especially in distinguishing acute fractures in a multiple fracture setting (109) and is consistent with MRI in patients with osteoporotic vertebral compression fractures (109).

Limitations: SPECT should be avoided in children unless oncologic or inflammatory conditions are suspected (110).

Strengths: The correlation of skeletal standardized uptake values (SUVs) and BMD suggests its use for clinical and research purposes (108).

In mice, SPECT scanners designed for pre-clinical models, can have a spatial resolution of <0.5mm due to pinhole and multi-pinhole collimators (111). This is useful when used in combination with CT scanning (SPECT/CT) which allows co-registration of the area of activity, and the skeleton, hence areas of new bone formation or high bone turnover (112). Multi-pinhole SPECT has successfully been used to track bone growth and repair in a mouse model for 12 weeks – specifically to track the temporal and spatial positioning of hydroxyapatite deposition in a bone defect mouse model (113). Benefits of this technique in mice are the non-invasive character allowing longitudinal tracking of changes in individual animals, which may reduce cost, animal numbers and inter-animal variability. Fast scan times (minutes) require less time under anaesthetic. However, exposure to radiation is required and this may be significant if repeated scans are taken.

In zebrafish, as for other radioactivity-based measurements, no special scintigraphy technology is available due to the water-based environment.

#### 3.1.3 Quantitative CT

Quantitative CT (QCT) enables 3D imaging of bone *in vivo* while providing quantitative information about the spatial bone density distribution at a resolution of around 0.5 mm (114, 115). The possibility to calculate vBMD provides a true density measure of the whole bone cross-section, in contrast to areal BMD obtained from DXA. QCT images can also be used as the basis for Finite Element (FE) models (116, 117). With such models, the bone geometry is represented by a large number of sub-volumes (the ‘elements’), typically one or a few mm^3^ in size. As the QCT resolution is not enough to resolve the trabecular or cortical microstructure, these models represent bone as a ‘continuum’ in which the bone microstructure is homogenized and represented by its density only (116, 118). Using such models, it is possible to calculate the bone stiffness, the stresses in the bone and the bone strength for a specified set of forces (‘boundary conditions’) (116, 119, 120). Such loading conditions can represent physiological loading (e.g. vertebral forces in the spine, or hip joint forces) to calculate physiological stress values in the bone tissue, or can represent loading conditions that typically lead to fracture (e.g. a fall) to calculate bone strength. QCT can be used in clinical trials aiming at quantifying the effects of drugs or other treatments on bone strength or in research studies correlating e.g. nutrition, lifestyle or genetic factors with bone strength. In addition, images can be analysed as an “add-on” screening tool in cases where QCT images are made for other reasons, e.g. during virtual colonoscopy or cardiovascular research focus, in which vertebrae are in the field of view (116, 121, 122). In both human and animal, FE modelling of bone in young versus older ages may differ. In particular, the growth plate can lead to artefacts, as these may appear as gap regions. In addition, the tissue mineralization in young versus old bone can differ, which may require using different empirical relationships to translate density to material properties.

Limitations: QCT images involve considerable costs, radiation dose and operational time. QCT based FE is not suitable as a screening tool (120). It is not possible to account for bone microstructure (other than its mere density), and thus empirical relationships between bone density and material properties are needed. Routine use of QCT in children is not established.

Strengths: A particular strength of QCT-based continuum FE models is that the technique is based on well-validated mechanical principles. This is in contrast to stochastic relationships that predict bone strength from bone density and structural parameters, for which no underlying theoretical relationship exists.

QCT based continuum FE is less suitable for small animals because of the smaller size of their bones (123). Thereby, the assumption that the bone microstructure can be homogenized to a continuum becomes less accurate. The resolution is not enough to resolve thin cortices. For small animals, the use of high-resolution micro-finite element analysis (micro-FE) therefore is more appropriate.

See section 3.1.6 MicroCT (µCt).

#### 3.1.4 Peripheral Quantitative CT (pQCT)

Peripheral quantitative CT (pQCT) is used to image the radius and tibia. The spatial distribution of fat, muscle and bone within the cross section is obtained after applying density thresholds for each of these tissues. At diaphyseal sites, cortical bone indices are obtained including cortical vBMD, periosteal circumference and cortical thickness, as well as muscle and fat cross-sectional area (124). In addition, estimates of cortical bone strength can be generated, such as cross-sectional moments of inertia. At the distal radius (i.e. metaphysis), trabecular vBMD is obtained at a pre-defined central region of the medullary space.

Limitations: A disadvantage is the poor standardisation at distal sites (positioning of reference line relative to the growth plate), which in children and growing animals limits reproducibility. In addition, in humans and animals alike, the partial volume effects (the situation where a voxel volume is only partially filled by bone tissue) lead to size artefacts, i.e. cortical vBMD is artificially reduced in individuals with reduced cortical thickness as a larger part of the voxel is not within the bone tissue. Adjustment for bone length may be required for subjects with tall or small stature, to correct for bone size.

Strengths: The ability of pQCT to evaluate cortical and trabecular vBMD, structure and strength variables separately represents an important advantage compared to DXA scans, and has provided the basis for separate genetic studies of cortical vBMD (125), trabecular vBMD (126) and cortical thickness (127). This may be even more important for the assessment of bone conditions where the relations between cortical and trabecular bone are shifted (128). Furthermore, pQCT scans are associated with a relatively low radiation dose, making this method suitable for clinical studies.

In mouse models, pQCT is useful to accurately measure both trabecular and cortical vBMD, as well as predicting bone strength (129). pQCT has been shown to be accurate and precise in mouse models, confirmed by both µCT and histology (130). It can be used *in vivo* on live animals. There is however the potential for errors in vBMD measurements based on specimen thickness and positioning (129, 131).

See section 3.1.6 MicroCT (µCt).

#### 3.1.5 High Resolution Peripheral Quantitative Computed Tomography (HR-pQCT)

Like the classical pQCT, HR-pQCT assesses bone microarchitecture in the cortical and trabecular compartments, but with higher resolutions of 82 μm for the first-generation devices and 61 μm for the second-generation devices (132, 133). Depending on technical developments, the clinically standardized volume of interest (VOI) is to be set to 9.5 mm in length for the first generation and 10.2 mm for the second-generation of devices. In adults, the beginning of the VOI is situated at a fixed distance proximally from a reference line through the joint at the distal cortex of radius and tibia (134). Thus, in taller individuals, the VOI is relatively more distal and has greater cross-sectional and trabecular areas as well as thinner cortices. For HR-pQCT imaging, the participant’s extremity is placed in a cast, which reduces motion. The cast is then inserted into the device and is fixed in position, while the x-ray source rotates around the extremity. The scanning time is around 2.5 min for these standard measurements. Cortical and trabecular vBMD, cortical and medullary cross-sectional area, cortical thickness, cortical porosity, trabecular spacing (Tb.Sp), trabecular number (Tb.N = 1/Tb.Sp) and Tb.Sp standard deviation are typically reported and satisfactorily accurate (135). For the first generation HR-pQCT only Tb.N was directly measured, while other parameters were derived from Tb.N and BV/TV using standard methods adapted from histomorphometry (134). For the second generation all parameters were measured directly. HR-pQCT is used for longitudinal assessment of changes in bone microarchitecture, e.g., of age-related bone loss (136). However, bone loss at the endocortical bone surface results in trabecularisation of the inner cortex and errors in estimation of cortical and trabecular bone loss (137). More recent software permits to transfer the initial endocortical contour on the follow-up scans and assess bone loss in the cortical and trabecular compartment. HR-pQCT scans may be used for micro-FE analysis to estimate bone strength (138, 139). In addition, vascular and tissue calcifications are targets of HR-pQCT measurements and currently under development.

Limitations: For now, HR-pQCT is available only for distal extremities, although with the second generation scanning of areas up to the knee and elbow has become possible as well (140, 141). As HR-pQCT is sensitive to movements, some scans have to be excluded due to poor quality. The occurrences of movement artefacts are higher for radius than for tibia scans, probably because the sitting position is less comfortable. Furthermore, the necessity to stay in a resting position without any movement of the scanned limb might be challenging especially in the elderly patient with tremor and people with pain in joints may have trouble being positioned. Any x-ray based method is not conclusive in areas with metallic or other implants (133).

In the structural analysis, identification of the endocortical limit between cortical and trabecular compartments by software is challenging (142). Thus, estimates of cortical thickness and area and that of trabecular area may be inaccurate. Manual analysis is time-consuming and has only moderate-to-good reproducibility. However, the endocortical limit on the HR-pQCT scan is not always evident even for experts and the manual analysis does not improve its identification. Several algorithms assess cortical porosity, but they are based on unverified assumptions (143–145).

Strengths: HR-pQCT is the only existing non-invasive imaging method obtaining bone microarchitecture in clinical studies. It is fast and safe (low radiation dose 3 µSv/scan), has good reproducibility (<1% for vBMD, <4% for structural variables) and gives no side effects (146).

HR-pQCT permits to assess the structural basis of the effects of the risk factors of osteoporosis (e.g., sex steroid deficit), predict fragility fracture and the effect of anti-osteoporotic treatments on bone (147–149). The biomechanical parameters assessed by micro-FE can improve fracture prediction (148). Scanning protocols for children are being developed.

For mouse and zebrafish context see section 3.1.6 MicroCT (µCt).

#### 3.1.6 MicroCT (µCT)

Micro-computed tomography (MicroCT or µCT) is a high-resolution imaging modality that offers quantitative analysis of trabecular and cortical bone morphology in animals and human specimens. First introduced in the late 1980s (150), µCT now has become the gold standard for the evaluation of bone microarchitecture throughout species.

The method, such as the other CT-methods, is based on the use of x-rays to create cross-sections of an object. For µCT voxel sizes lower than 10 μm can be obtained (151). The degree of x-ray beam absorption is recorded, so that the 3D structure of the object can be visualized and numerous bone structural parameters can be quantified with a high degree of accuracy, such as cortical and trabecular vBMD, cortical thickness, and if used at high enough resolution/voxel size, cortical porosity.

As an *ex vivo* imaging modality in humans, µCT enables 3D characterization of small bone specimens acquired from bone biopsies, or of larger cadaveric specimens such as vertebrae (152, 153). Studies have shown that µCT can reproducibly quantify 3D microarchitecture of the trabecular and cortical bone in iliac crest biopsies, demonstrating significant changes in 3D trabecular structural parameters in postmenopausal samples, including a decrease in BV/TV, an increase in trabecular separation and a shift from platelike to rodlike structure (154). µCT quantification of bone structure from iliac crest biopsies is an important end point in longitudinal drug efficacy studies (155). Assessment of 3D trabecular and cortical structural characteristics may improve our ability to understand the pathophysiology of osteoporosis, to test the efficacy of pharmaceutical intervention, and to predict bone biomechanical properties.

Limitations: Due to the high radiation exposure, the use in humans is restricted to *ex vivo* measurements and thereby limits the clinical application of µCT. High-resolution scans produce large amounts of data that require support for data acquisition, processing and management. Even though a considerable limitation of the µCT technology is the lack of specificity for soft tissues, it can be combined with contrast agents for the visualization and quantification of soft tissues like vascular structures and bone marrow adiposity within the bone specimens (156, 157).

Strengths: Compared with histology, µCT has many advantages as larger volumes are analysed, 3D-measurements can be performed faster with higher resolution, excellent reproducibility and accuracy. The assessment of bone morphology is non-destructive and does not require fixating agents, enabling subsequent analyses of specimens for histology, mechanical testing and biochemical analysis.

In mice, µCT is a widely used method for analysing bones of small animal models *in vivo* and *ex vivo*, due to its high resolution, with the ability to achieve resolutions as small as 1-micron (158) and identifying body composition. Guidelines for µCT assessment of rodent bone specimens have been recommended including sample preparation, image acquisition, processing and analysis (159).

*Ex vivo* µCT can be used to measure cortical and trabecular vBMD, cortical thickness, and if used at high enough resolution/voxel size, cortical porosity (160). It has been used at high-throughput format to identify bones with altered BV/TV, trabecular thickness (Tb.Th) and trabecular number (Tb.N) (66) and can be used for longitudinal assessment of the same animal over time due to the non-destructive character. It can be performed on living animals, although long scan times do require large doses of anaesthetics and radiation (above 400 mGy/scan) can affect osteoblasts and subsequent evaluation of bone formation (161, 162).

In adult zebrafish, µCT is a well-established and widely used tool for the detection of skeletal abnormalities (13, 40, 163–165). Due to the small size of the bones in zebrafish, the visualisation of the skeleton through standard µCT has been mostly limited to skeletally mature animals. The use of contrast agents, such as AgNO3, has been shown useful for the visualisation of earlier ages of the zebrafish skeleton, as well as for soft tissue [28]. 3D tissue mineral density (TMD) reflects the amount of mineral per unit volume of bone tissue and is used to measure cortical TMD in zebrafish. TMD values of 450-600 mg HA/cm^3^ have been reported in the vertebrae of adult zebrafish (13) which is noticeably less than the TMD values of 800-1000 mg HA/cm3 in the cortical bone of adult mice (166) or human cancellous bone (167). These differences in TMD have been attributed to differences from human bone in material properties and mineralization dynamics (12) and as a possible reflection of adaptation to mechanical loading and bi- or quadrupedalism in terrestrial mammals (168). In parallel with TMD, values of the vertebrae length, area, volume, thickness and other measurements of shape are often used for phenotypic characterisation of the zebrafish vertebral column (13, 164, 169). The vertebral column, as a major skeletal structure of the zebrafish adult skeleton, is most commonly studied by µCT. However, it is also used for the analysis of other parts, such as the zebrafish craniofacial skeleton (11, 164, 170, 171). Semi- to full automation of bone segmentation from µCT imaging data would allow rapid and robust analysis. In this line, a supervised segmentation algorithm (Fish-µCT) enables segmentation of each vertebrae and profiling of phenotypic measures (13, 39).

### 3.2 Magnetic Resonance Imaging of Bone (MRI)

Magnetic resonance imaging is an intersectional imaging method. It technically uses a combination of a strong magnetic field (1.5-9T) and stimulation of protons by radiofrequency pulses. MRI provides high contrast resolution and better soft tissue display than computed tomography. Due to different imaging techniques, like fat suppression, it provides a high sensitivity for findings like periosteal edema and bone marrow changes as well as intracortical signal abnormalities (172). Frequent findings in acute and subacute vertebral fractures are vertebral edemas with a low signal on T1-weighted images (WI, using basic pulse sequences in MRI) and a high signal on T2-WI, and high signals on STIR (Short tau inversion recovery), while old fractures show the opposite (109). MRI has been analysed for “M-scores”, deviated from signal to noise ratios (SNR) in the vertebrae L1-L4 as compared to T-scores using DXA. The SNR in L1–L4 is negatively related to BMD, but the cut-off value for M-scores is still under debate (173). Some of the novel MRI imaging techniques are able to quantify bone composition and may generate precise phenotypes of bone changes related to age (174). Future developments should define calibration phantoms for routine imaging. Artificial intelligence (AI) algorithms may be used for existing images to identify patients at risk for bone fractures.

Limitations: MRI requires expensive equipment and training. A direct comparison to 2D DXA is not possible, and limited resolution is often not sufficient for morphological analysis. Especially high field MR scanners (over 7T) are not widely available and costly, therefore only accessible in well-equipped institutions. In general, this equipment is exclusively used for research purposes. MR examinations are time-consuming due to the longer scanning time and therefore more susceptible to motion artefacts, which can affect the accuracy of evaluations. Furthermore, MR is very susceptible to artefacts caused by metallic implants, for example postoperatively in the case of spondylodesis, which in turn reduces image quality and makes partial evaluations impossible.

Strengths: MR technologies are widely available, at least in developed countries. There is no radiation exposure, therefore repeated and large areal scans are possible. Well-defined morphological tools may help to characterize significant changes in clinical work-up. A powerful strength of magnetic resonance is the excellent soft tissue imaging. It is superior for imaging muscle pathologies and, through special techniques such as the Dixon technique (175), for quantifying adipose tissue and muscle mass in a reasonable time frame, which might also add important information in connection with osseous pathologies. Furthermore, cartilage damage and degenerative as well as inflammatory changes in articular cartilage and intervertebral disc tissue can be identified and quantified, which is not technically possible to the same extent using computer tomography.

MRI is a useful tool in mouse phenotyping as it allows concurrent imaging of soft tissue (cartilage, bone marrow, muscle, fat) and bone with good spatial resolution. MRI has been used to image bone injuries in mice with good distinction between the bone, soft tissue and injury sites, with a good signal-noise ratio (176). MRI is particularly useful for monitoring endochondral fracture healing, which involves a cartilaginous tissue callus (177). MRI has the benefit of providing 3D images, and allowing longitudinal assessment of single animals. Disadvantages include a long scanning time (up to hours), the potential for artefacts at the bone-soft tissue interfaces, and low resolution due to the size of the sample (176, 178).

MRI has not been widely used in zebrafish. However, recent studies demonstrated the use of the imaging technology for longitudinal and non-invasive studies. 3D scans covering the thoracic region of the same adult zebrafish at an isotropic voxel resolution of 31 µm allowed longitudinal studies of the zebrafish heart. Bone and muscles were observed with MRI (179). To overcome the limitations of the aquatic system, a flow cell system has been developed for MRI imaging, allowing to monitor the zebrafish during the scan and to fully recover the animal (180). However, the methodology needs to be further improved to establish it as a routine bone assessment in zebrafish.

## 4 Bone Biopsy and Local Measurements

Investigations at the tissue level have a long tradition for histology and several microscopic technologies which are important in clinical practice for the differential diagnosis of disease entities. However, new approaches will help to expand our understanding of bone properties using microindentation (see *Microindentation*) or compositional bone matrix analyses *via* quantitative backscattered electron microscopy imaging (qBEI) and vibrational spectroscopy (see *Compositional Bone Matrix Analysis Using Quantitative Backscattered Electron Microscopy Imaging (qBEI) and Vibrational Spectroscopy*) – these new approaches and their use in human and animal bone research is of increasing importance.

### 4.1 Histology

Histology of bone biopsies provide qualitative information about bone cells, matrix (e.g. the orientation of collagen fibres), mineralization and bone marrow. Evaluation of bone biopsy should comprise its histological (visual, qualitative) and histomorphometric (quantitative) assessment (181). The biopsy should be examined for the presence of mast cells and cancer cells infiltrating the bone marrow or the bone. It should be noted if the bone has the normal lamellar texture or if woven bone is present (182).

Limitations: Histology only provides information on the 2D structure of tissues and cells which can lead to an over- or underestimation of morphological features. However, the stacking of layers can be applied to regain 3D-information. It is a destructive method, and only the remaining parts of an embedded sample can be analysed with other techniques than the histological assessment.

Strengths: Histology is one of the most established and versatile methods to identify different types of tissues, and osseous cell components at high resolution. Various staining protocols are readily available for the detection of bone matrix alterations due to diseases or treatment.

In mouse and zebrafish studies, histology is widely used. As with human studies, it can provide information on cell type and number, bone matrix and mineralisation and help to characterize specific disease models.

### 4.2 Histomorphometry (Static and Dynamic)

In addition to specific histology, a histomorphometric evaluation of bone modelling and remodelling can provide quantitative information about mineralization disorders, metabolic bone diseases, and secondary bone diseases including cancer. “Static” bone histomorphometry (HM) consists in counting cells and measuring bone tissue components. For “dynamic” purposes, oral tetracyclines are administered separated by 10-12 days. Tetracycline is incorporated into new bone at the “mineralization front”, and its fluorescence allows for the assessment of bone turnover (183–185).

Histomorphometry from patients requires bone biopsies obtained standardly from the iliac crest under local or (in children) general anaesthesia.

Bone samples are processed without prior decalcification according to published protocols. The stains should allow the differentiation between mineralized bone tissue and osteoid, and the identification of bone and marrow cells by using several methods, with Goldner’s trichrome and toluidine blue being most widely used. Solochrome cyanine R allows the observation of bone texture under polarized light. Unstained sections are prepared for the observation of the tetracycline labels by fluorescence microscopy. May-Grünwald-Giemsa or toluidine blue are used for the analysis of bone marrow and especially for the identification of mast cells and TRAP-staining is common to assess osteoclast parameters.

Quantitative analysis is performed on complete and unbroken samples. Measurements are performed by using automatic or semi-automatic image analysers. Parameters can be measured separately on periosteal, cortical, endocortical and cancellous bone. The bone histomorphometric parameters with abbreviations have been standardized by the American Society for Bone and Mineral Research (ASBMR) Histomorphometric Nomenclature Committee (186, 187).

For some specific diagnoses histomorphometric examination is required. For example, osteomalacia shows an accumulation of osteoid i.e. non-mineralised bone. While the experienced examinator can give the diagnosis of osteomalacia without quantification, the degree of the delay of mineralization requires HM. Hyperparathyroidism (HPTH) is associated with high bone turnover and an increased amount of immature bone showing a diverged picture from the usual lamellar structure referred to as woven bone, as well as marrow fibrosis. An important indication for bone biopsies is chronic kidney disease (CKD) with potential high or low turnover conditions. In mild CKD, changes may be similar to HPTH with woven bone and peri-trabecular marrow fibrosis, referred to as osteitis fibrosa. Osteomalacia and adynamic bone disease are showing with low turnover features in bone histomorphometry. Both conditions require careful therapy adaptation. Bone fragility disorders such as osteogenesis imperfecta are associated with typical static and dynamic HM. Reference values of healthy children and adolescents (188), adult osteoporosis (183) as well as patients with OI type 1 (189) are used for interpretation of HM results.

Limitations: This invasive method depends on established procedures and trained personnel. Localised bone diseases like Paget’s disease of bone and fibrous dysplasia are usually not seen in iliac biopsies. Analysis is performed on an iliac bone sample, an unloading site not prone to fracture in contrast to vertebra, forearm or femoral neck. Despite differences in microarchitecture and turnover between iliac crest and the other skeletal sites, significant correlations were found (190).

Strengths: Bone HM remains the only method allowing the study of bone at the tissue and cell levels to enable measurements at intermediary levels of organization of bone i.e., the osteon. It also remains the only established method to diagnose osteomalacia.

In the study of mouse bone, both static and dynamic histomorphometry are widely used mostly on sections of the distal femoral metaphysis and, for cancellous bone, in the appendicular skeleton. Several staining methods are used to measure osteoblast parameters, such as Toluidine blue or Von Kossa and McNeal stain (191). Osteoclast parameters additionally are measured using the TRAP staining (192). In dynamic histomorphometry, bone formation and apposition rates are calculated using a timed fluorescent agent which is incorporated into newly formed bone, much like described for human studies. Fluorochromes such as calcein, tetracycline and alizarine red (193), can even be combined for double labelling that has demonstrated both increased and reduced bone formation and mineral apposition rates in cortical and trabecular bone (160, 192).

As in human and mice, static and dynamic histomorphometry are used in zebrafish. Vertebral endplates are active sites of bone formation, providing a suitable region for typical static bone histomorphometry (50). Number of osteoblasts per bone perimeter (N.Ob/B.Pm), osteoid thickness (O.Th), osteoid surface per bone surface (OS/BS) and osteocyte density (N.Ot/B.Ar) can be assessed (40). As zebrafish are transparent during skeletogenesis and through juvenile stages, bone staining are often readily observed in whole-mount, alleviating the time and resources required for tissue sectioning. Alizarin Red and Calcein staining are used to label mineralizing tissues (194), which can be monitored *in vivo* in bones that are optically accessible, such as early developing vertebrae, growing vertebrae, scales and adult fin rays. Pulse labelling with Alizarin and Calcein can demarcate bone formation between labelling periods (50, 195–197), similar to dynamic histomorphometric approaches in mammals. Mineralised bone can also be assessed by Von-Kossa staining and activity of alkaline phosphatase (ALP) (198). Cartilage is frequently visualized using Alcian Blue (199–201). Moreover, the use of transgenic lines also allows *in vivo* assessment of specific cell types, including osteoblasts (202, 203), osteocytes (203) and osteoclasts (204, 205). Osteoclast activity can be observed in whole-mount or histological sections using TRAP staining (206).

### 4.3 Microindentation

Micro- and nano- indentation have been used in many studies to quantify the modulus (stiffness) and hardness (resistance to yielding) of bone tissue (207, 208). A limitation of these techniques is that they can be applied only to extracted bone samples or biopsies. Reference Point Indentation (RPI) estimates the resistance of the cortical bone to fracture (209, 210). It is based on the hypothesis that the microindentation of the bone surface induces the separation of mineralized collagen microfibers and the initiation of micro-cracks (211). Whereas this measure is related to the resistance of bone tissue to fracture, it is incompletely understood which mechanical properties of bone are captured by RPI. For this reason, measurements typically quantify a parameter called Bone Material Strength index (BMSi) units representing the ratio between the penetration of the probe into the bone and its penetration in a methyl methacrylate reference phantom (209, 210). In RPI, a probe is applied to the outer surface of the cortical bone of the tibia under local anaesthesia to produce a microindentation (of a size similar to a resorption lacuna), and thus, to measure the distance the probe can penetrate the bone. The higher this distance, the less the bone is able to resist the formation and propagation of micro-cracks, and thus the weaker it is (210).

Two distinct RPI techniques exist (208). The first to be developed was the cyclic reference point microindentation (CMI) using the BioDent™ device (211). CMI was used in the first human clinical studies and is currently the most used technique in animal studies (209). The second technique called impact microindentation (IMI) is conducted with the Osteoprobe R device (212). IMI was developed for *in vivo* use in clinical studies exclusively from 2013 on (210), and in larger animals (209). As the two techniques differ in mechanical challenges, and do not exactly measure the same mechanical properties, the preclinical results from CMI cannot be extrapolated to clinical results from IMI (209).

Limitations: The use of IMI in the clinical practice is still hampered by methodological and technical limitations and the lack of reference values validated according to ethnicity, sex and geographical regions. The development of standardized procedure (213) and future prospective multicentre studies will clarify the benefit of the methods for the assessment of the pathophysiology and the response to treatment interventions (208–210).

Strengths: This technique holds great promise as it provides clinicians a minimally invasive, simple and safe tool for assessing the material properties of bones *in vivo* (209). Existing data support IMI as a valuable technique for the assessment of bone fragility in research studies and possibly for its follow-up (209, 210). Importantly, IMI has been proposed as an additional tool to assess and comprehend bone quality, instead of replacing the existing techniques (208–210).

In mice, micro- and nanoindentation is not a commonly used technique, but has been used to assess bone homeostasis and bone repair following micro-damage (214, 215). Among the techniques, RPI is used most frequently. Benefits are the capacity for longitudinal, *in vivo* assessment of the mechanical properties of bone. However, during *ex vivo* technique validation, it has been shown that RPI testing data is poorly correlated with fracture data from traditional biomechanical testing, and has relatively large variability (216, 217).

In zebrafish, nano-indentation is used instead of micro-indentation due to the small size of the zebrafish bones (vertebral length ~ 500 µm, and width ~ 50 µm). It allows the determination of local mechanical properties in sagittal or transverse planes of individual zebrafish bone (40, 218–220). Specifically, the modulus of elasticity, hardness, and modulus-to-hardness ratio (E/H; used as a surrogate measure bone fracture toughness (221) can be extracted and correlated to compositional parameters. In this context, an increase in mineralization under physiological conditions, e.g. with aging, results in an increasing elasticity of zebrafish vertebrae, homologous to human and mammalian bone in general. However, in case of a more disoriented bone matrix, e.g. due to collagen pathologies, an altered organization of the mineral has been correlated with a decrease in mechanical performance. Given the high resolution of nano-indentation experiments, i.e. penetration depth of several 100 nm with a Bercovic tip, heterogeneities in mechanical performance can be assessed, e.g. vertebral end plate region vs. vertebral centrum.

### 4.4 Compositional Bone Matrix Analysis Using Quantitative Backscattered Electron Microscopy Imaging (qBEI) and Vibrational Spectroscopy

Blocks from bone biopsies can be analysed using quantitative backscattered electron microscopy imaging (qBEI). For qBEI, specimens are commonly embedded, polished coplanar and coated with carbon to provide stable electron conductivity. Assessed is bone mineralization density distribution (BMDD), reflecting the calcium content of cortical and trabecular bone matrix (222–224). With qBEI, the phenotype of several conditions can be further delineated, for example the typically elevated bone tissue density in osteogenesis imperfecta (223). Using a backscattered electron (BSE) detector, variations of intensity of the BSE-signal are measured. Backscattered electrons interact mainly with the sample surface, whereby the intensity is dependent on the local mean atomic number of the sample. Calcium, being the heaviest element in bone, is used to quantify the degree of mineralization based on a linear correlation between the calcium content and the grey value of the BSE image. With the help of reference materials, the brightness and contrast of the image are calibrated and the mean calcium weight percent distribution can be determined based on the grey value histograms of the BSE image, which allow to assess the average calcium content in the mineralized bone tissue area (Ca_mean_), the heterogeneity of mineralization (Ca_width_), as well as areas of high and low mineralization (Ca_high_ and Ca_low_, respectively).

Whereas qBEI provides compositional information mainly on the inorganic component of bone, vibrational spectroscopy can be used for the simultaneous analysis of mineral- and protein-related parameters in bone. The identification of molecular components based on their energy-specific vibrations is used in both Fourier-Transform Infrared spectroscopy and Raman spectroscopy (225). In the context of bone quality assessment, vibrational spectroscopy is a specialized tool to evaluate the “structural fingerprint” for the identification of molecular bonding involved. During vibrational spectroscopy, the sample is irradiated with a specific wavelength, which leads to changes in the vibrational modes of specific molecules, allowing to detect mineral-related components in a spectrum (peaks of phosphate, carbonate) and protein-related components (peaks of amide I and II, phenylalanine, hydroxyproline and proline). Typical Raman and FTIR parameters of bone quality include the mineral-to-matrix-ratio (e.g. phosphate-to-amide I, indicative of the degree of mineralization), the carbonate-to-phosphate ratio (indicative of carbonate substitution in the crystal lattice), and crystallinity (fill-width-at-half-maximum of the phosphate peak, related to crystal size), e.g. with different reactions of bound-water compartments for collagen and mineral-bound water. Certain aspects of collagen are also assessable (226). For a more in-depth view also on strengths and limitations, please see e.g (227).

Limitations: Electron microscopy is a complex method, depending on specific clinical/research questions and requires established highly specialized procedures and trained personnel. Moreover, both qBEI and vibrational spectroscopy are generally limited to 2D information.

Strengths: True bone density distribution at the tissue level is measured. Additional information on molecular components and mineralisation enables for new approaches in the interpretation of bone metabolism and structure.

For studying mouse bone, backscattered electron scanning electron microscopy is particularly useful. Resin embedded samples have been used to determine local tissue level mineralisation of bone and the high resolution allows for the investigation of lacunar properties, such as their size and appearance of their surface. Osteoblasts and osteocytes bound to the surface of the specimen can be assessed for their various phenotypic stages. Macerated, non-embedded samples can be used to identify changes in microarchitecture and surface values (192, 227).

Similar to performing qBEI and vibrational spectroscopy in humans, zebrafish bones can be investigated in terms of calcium content and heterogeneity of mineralization as well as Raman spectral parameters. For instance, an increased calcium content has been observed after exercise of zebrafish, as well as in zebrafish OI models (40). Raman and Fourier-transform infrared (FTIR) spectroscopy (19) imaging in zebrafish have shown that zebrafish bone contains carbonated hydroxyapatite as well as other mineral phases, similar to mammalian bone. Moreover, in a zebrafish model of OI, lower matrix maturity is confirmed through reduced collagen maturity and altered carbonate-to-phosphate ratio using FTIR (40).

### 4.5 Immunohistochemistry of Bone (IHC)

Immunohistochemistry (IHC) allows to determine the cellular localization of proteins and their expression within tissues (228). This technique requires two phases: 1) specimen fixation and tissue processing and 2) interpretation and quantification of the obtained expression (229). Tissue properties can be analysed in depth, making it possible to study not only bone but also the surrounding tissues like cartilage, muscle and tendons. There are different approaches in immunohistochemistry analysis and reporting and for some IHC markers like bone morphogenic proteins (BMP), osteocalcin (OCN), osteopontin (OPN), and few others scoring systems are available (230), which may include IHC markers of the surrounding tissue. Sequential antibody immunostaining for quantification is used to detect antigens of interest. This is a complementary method to *in situ* hybridization histochemistry (ISHH) which detects cellular nucleic acids based on the formation of double-stranded hybrids between a nucleic acid fragment (the probe) and a DNA or RNA sequence present within bone cells (231).

Limitations: Decalcified bone samples are widely used for IHC. However, during the decalcification process, the integrity of the trabecular network is lost, which can cause changes in the overall appearance of the bone morphology. Careful specimen preparation and analysis by experienced researchers should be applied. As an alternative, methyl methacrylate embedding retains the mineral fraction of the bone tissue. However, the hard embedding makes sectioning more difficult and epitope retrieval complex (228). The process of optimization of the method for each target can be relatively time-consuming, costly and labour intense.

Strengths: For humans, a great variety of antibodies are available and established for IHC. On its own and in combination with different antibodies and other staining techniques, this enables a multitude of possibilities to visualize complex interactions.

In mouse studies, IHC can provide useful information on the temporo-spatial expression of key factors important for musculoskeletal development and function. However, careful and experienced specimen handling of the small mouse samples is needed due to the tendency for samples to detach from slides. IHC has been used to locate many important antigens in bone such as SOX9, OSX and sclerostin (232, 233). As in humans, decalcified bone specimens are most commonly used.

Immunohistochemistry is applied in histological sections (234) and whole-mount zebrafish samples, often performed in larval stages and dissected adult tissues (171, 235–237). While a plethora of antibodies are available for human and mice proteins, only few are available for zebrafish. Antibody tests and protocol optimization need to be performed in zebrafish as for mice and human samples.

## 5 Biochemistry for Bone Phenotyping

In patients, a number of general laboratory analyses are necessary to assess a patient’s general health and potential causes of secondary bone disease (238, 239). These include a full blood count, erythrocyte sedimentation rate (ESR) or C-reactive protein (CRP), markers of liver and kidney function and markers of calcium/phosphate metabolism. Further, optional tests include serum proteins (including electrophoresis; to exclude multiple myeloma), markers of thyroid function (to exclude thyrotoxicosis), sex hormones (to exclude hypogonadism) and measurement of free cortisol in 24-hour urine for screening for Cushing’s syndrome. Some additional tests might be useful to exclude other pathologies, e.g. celiac disease *via* transglutaminase antibodies or systemic mastocytosis *via* serum tryptase and/or urine methyl histamine.

Limitations: Optional laboratory parameters require a diagnostic plan for the individual patient to be useful and may be more expensive.

Strengths: Differential diagnoses are frequently based on the knowledge about general health conditions and should be available in acceptable quality.

In mice, most studies use a defined mouse model for distinct research questions. Therefore, it is not necessary to perform biochemistry for the diagnosis of a disease in this context. However, biochemistry can be performed on blood and plasma samples. Other than in humans, the amount of blood obtained from the living animal can be a limiting factor for such tests. They are therefore mostly performed with terminal blood collection in mice.

In zebrafish, although neither pregnancy nor lactation exist, the sex-hormonal changes and sexual-development milestones (analogous to “puberty” or “post-reproductive” age) are well characterized. Sex in zebrafish (as well as amphibians and reptiles) is not determined by a particular chromosome, but by the interaction between gene and environment. Sex hormones are well studied, hypogonadism could be easily obtained and well as orchid- and ovariectomy. Catecholamines, mineralocorticoids and microelements are measured similarly to mammals, as well as thyroid stimulating hormone and its receptor (13). Blood can be collected from the adult zebrafish through the aorta and decapitation. Recently, it has been shown that repeated blood collection can be performed from the same adult zebrafish longitudinally for the measurements of triglycerides and glucose (240). Due to the small size of zebrafish, a limitation of the method is the total blood sample volume that can be collected at time, ≤0.4% of body weight per week for repeated measurements.

### 5.1 Controllers of Bone Mass and Mineralisation

Many factors are involved in the regulation of bone mineralization, among them calcium, phosphate, calcitriol, fibroblast growth factor 23 (FGF23) and parathyroid hormone (PTH). A major clinical problem worldwide is vitamin D deficiency, which has attracted considerable interest over the last two decades (241). Active Vitamin D is the main supplier of bone minerals to bone tissue. In patients with sufficient vitamin D levels, additional supplementation has no effect on bone (242). Hence, recent studies in osteoporosis have seriously questioned the routine use of vitamin D supplementation for the purpose of preventing osteoporotic fractures (243). Bone mineralization disorders in general (osteomalacia/rickets) can easily be excluded, diagnosed and treated - there is global consensus for what constitutes sufficient vitamin D levels and calcium intake for the prevention of osteomalacia and rickets (244).

The calcium sensing receptor senses decreased dietary calcium supply and increases PTH secretion. Hence, PTH levels are inversely correlated with 25-hydroxycholecalciferol [25(OH)D] levels and dietary calcium supply. However, while PTH is generally known as an indicator of vitamin D status, there is no consensus regarding the accuracy of measuring PTH to determine vitamin D depletion (245). Dietary calcium supply is the likely reason for this (245). Through an unknown phosphate sensing mechanism, FGF23 controls renal phosphate reabsorption. Bone hypo-mineralisation (osteomalacia, rickets) only develops when serum phosphate is low (246) and is accompanied by elevated (bone) alkaline phosphatase and PTH concentrations.

Laboratory analysis of calciotropic hormones, mainly 25-hydroxycholecalciferol (25(OH)D) as a surrogate for the individual pool of vitamin D and several metabolites including the active 1,25-dihydroxycalciferol (1,25(OH2)D) have gradually undergone worldwide standardization with European and U.S. quality and accuracy methods including reference samples used as gold standards for both mass spectrometry and enzyme-linked assays (247). PTH measurements have undergone considerable development over the years, discriminating between the entire molecule and distinct fragments. Currently used assays identify intact PTH and assays for subforms are still available for specific questions.

Limitations: There is an urgent need to define non-invasive diagnostic criteria for osteomalacia (248). There is some discussion about the analytical approaches and the conversion of units used e.g. in 25(OH)D measurements. There is no single biochemical marker that represents normal bone mineralization.

Strengths: Bone mineralization disorders can be excluced by simple blood tests.

In mice, assays are available for testing serum levels of vitamin D and PTH in mice. Although these tests have been used in the study of dietary intervention and bone health, they are not regularly used in the routine monitoring of bone health in mice (249). On the other hand, a particular benefit of mouse models for studying skeletal disease is that they have less natural variation than humans. Identical diets, environments and genetics mean that mice should have minimal variation in vitamin D or PTH levels.

In zebrafish, measurements have not yet been performed *in vivo* in longitudinal studies. Upon fish decapitation, blood and serum readings could be potentially performed for levels of PTH, calcium phosphate (dependent on food intake), FGF23 levels and vitamin D. It would be interesting to test if such assays could be performed using small volumes of blood/serum that can be collected from zebrafish allowing longitudinal studies.

### 5.2 Bone Turnover Markers (BTMs)

**Figure 3:**
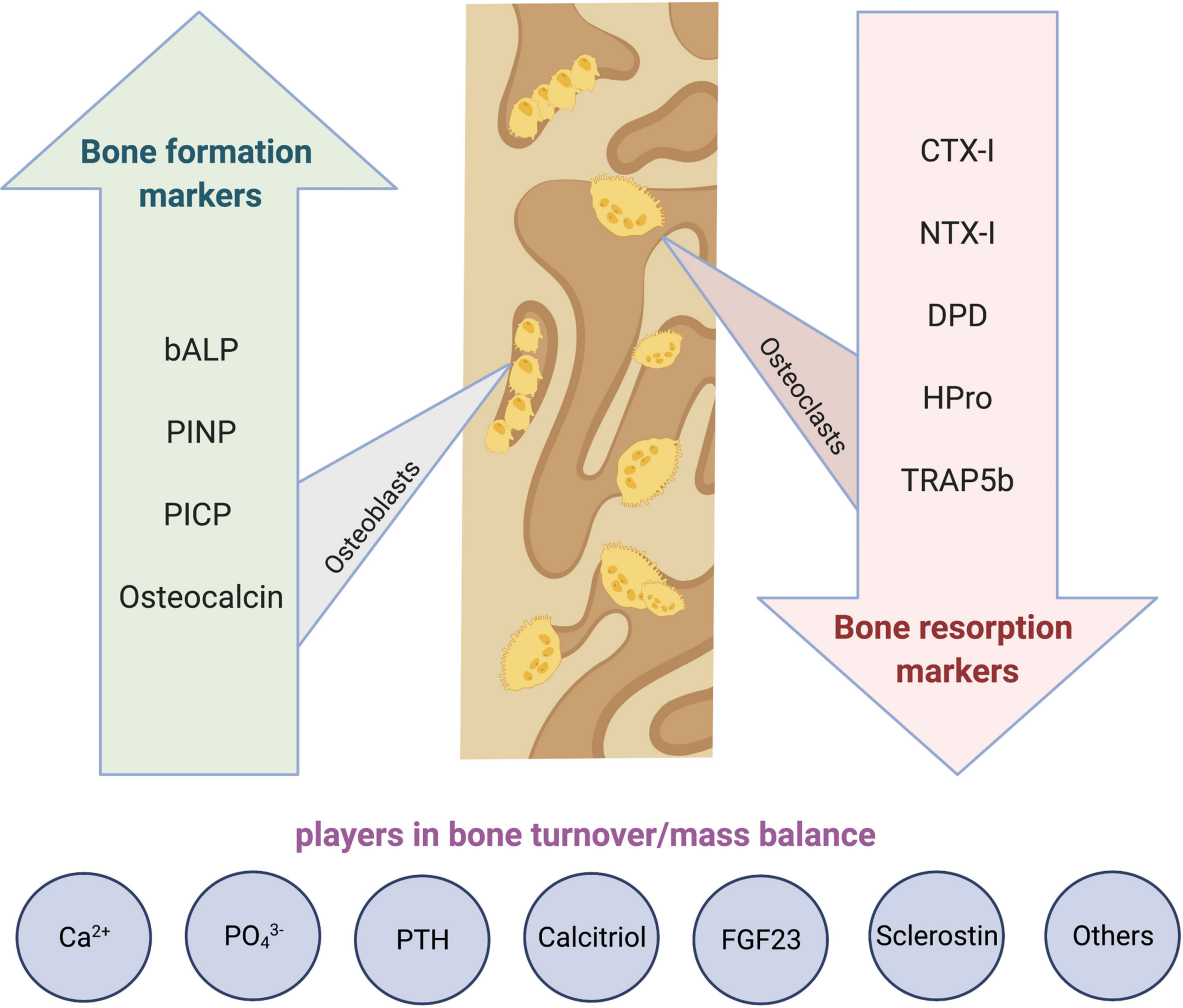
Bone turnover markers and players in bone turnover/mass balance. bALP; bone alkaline phosphatase, Ca^2+^; calcium/ionised calcium, CTX; C-terminal telopeptide of type I collagen, DPD; deoxypyridinoline, FGF23; fibroblast growth factor 23, HPro. hydroxyproline, PINP and PICP; N-terminal and C-terminal propeptides of type I procollagen, ${\text{PO}}_{4}^{2+}$ ; phosphate, PTH; parathyroid hormone, TRAP5b; tartrate-specific acid phosphatase type 5b.

Systemic markers of bone turnover (BTMs) reflect bone remodelling in adults (250), but also a combination of bone remodelling, modelling and 3-dimensional bone growth in children (251) (**Figure 3**).

Bone formation markers such as osteocalcin (OC), and N-terminal and C-terminal propeptides of type I procollagen (PINP, PICP) are proteins secreted by osteoblasts and represent the activity of bone formation. PINP is also expressed in other tissues (e.g., skin) and during fibrotic processes. Therefore, its concentration may be elevated in skin diseases and in case of active fibrosis (e.g., liver, lungs, heart) (252). Bone alkaline phosphatase (bone ALP) is an ectoenzyme present on the outer surface of osteoblasts. Their serum levels are correlated positively with histomorphometric measures of bone formation (e.g., osteoid surface, appositional rate, mineralization) (253).

The markers of bone resorption activity comprise C-terminal and N-terminal telopeptides of type I collagen (CTX, NTX), deoxypyridinoline (DPD) and hydroxyproline (HPro). They are products of bone collagen degradation. Blood and urinary levels of bone resorption markers reflect the activity of bone resorption. They are correlated positively with histomorphometric measures of bone resorption and decrease rapidly after administration of an anti-resorptive agent (254). Among bone resorption markers, CTX is the most specific for bone. Tartrate-resistant acid phosphatase 5b (TRAP5b) is an enzyme expressed by osteoclasts. It is an indicator of the presence (number) of osteoclasts, but not necessarily of their resorptive activity. Therefore, in some situations (osteopetrosis, treatment with cathepsin K inhibitors), discrepancy between the TRAP5b concentration and the levels of collagen degradation products may be observed (255, 256).

Serum PINP and CTX are defined as the reference markers of bone formation and bone resorption (257).

Limitations: Their specific technical and analytical limitations are categorized in two main groups (258):

Analytical variability: Despite the reduced analytical coefficient of variation (CV) of the techniques used to measure these markers, lack of a uniform standardization technique has resulted in difficulties in comparing values obtained by dissimilar methods in different laboratories. The intra-individual biological variability of BTMs is still of concern, especially when therapeutic approaches should be chosen based on a single measurement.

Pre-analytical variability: The BTMs’ preanalytical variability due to uncontrollable and controllable factors should be considered during their clinical interpretation. Uncontrollable factors are for instance age, sex, renal function, growth rate, pubertal or menopausal status, comorbidities or recent fracture. The use of appropriate reference ranges, in particular in children, and suitable adjustments can help overcome this variability to some extent. Controllable factors are for instance food intake/fasting status, circadian and menstrual cycle or exercise. The effects of these factors can be minimized by standardizing the timing and conditions of sample collection.

Measurements of BTMs are not helpful for diagnosis of osteoporosis, bone fragility or skeletal dysplasia. For instance, only 20% of osteoporotic women had serum CTX-I concentration exceeding the upper limit of the reference values in premenopausal women (259). However, elevated BTM levels (especially urinary bone resorption markers and bone ALP) may point to more rapid bone loss and higher risk of hip fracture in adults, mainly in postmenopausal women (260, 261). On an individual level, BTMs may be helpful for monitoring anti-osteoporotic treatments in adults (262) and children (263). Circadian rhythms and fasting are important factors in the clinical validation - blood samples for PINP can be collected at any time of the day due to only a slight circadian variation comparable to the measurement error of the assay (264). By contrast, blood CTX decreases rapidly after breakfast (265) and blood for CTX assay must be collected in the fasting status in the morning before 10 am.

In children, specific age- and development-associated reference ranges are used for most of the markers but the diagnostic value is limited (266).

Strengths: BTMs are non-invasive to bone. They closely monitor systemic bone turnover and are increasingly established and accessible. Standardization of the analytes is ongoing, but assay results may differ between providers.

BTMs are easily measured in mice using ELISA or commercially available assays. In an ovariectomized mouse (as a model of osteoporosis) OC and CTX provided conclusive outcomes on bone turnover when compared to µCT imaging (267, 268). Although serum BTMs hold the benefit of allowing multiple tests to be taken from one animal over a time course, it should be considered when planning an experiment whether more useful data can be gained by qPCR or western blot of bone tissue post mortem.

Bone formation (osteocalcin, alkaline phosphatase) and resorption markers (TRAP) are measured *ex vivo* through immunoassays in whole-mount, dissected tissues and on histological sections. For *in vivo* studies Alizarin Red and Calcein can be used to measure bone formation as well as the use of transgenic lines for osteocalcin, and TRAP line labelling osteoclasts (205, 269). *In vivo* longitudinal studies for such markers through blood and serum collection have not yet been performed in zebrafish.

## 6 New Phenotyping and Future Aspects

For the purpose of personalized medicine and to avoid a “one size fits all” approach, a differentiated pattern of patients’ characteristics might be a goal for future investigations based on age, sex and ethnicity and other background information, which should be taken into consideration. Duration of exposure to any harmful agent or environmental condition is important during the life course and of great interest from the epigenetic perspective. In the young, damaging environmental exposures have not yet accumulated. One example is obesity, where duration of exposure in relation to bone health is something comparatively little is known, but which could be explored in animal models.

There are numerous important new angles, e.g. the view on bone health early in life, shown by the recent insights in the paediatric field. Children with severe illness or skeletal disorders often have short stature, scoliosis, joint contractures and bone deformities. Hence, despite size adjustment, DXA data can be falsely low or unobtainable. Therefore, clinical assessment of bone health and research studies focus on defining bone phenotypes from bone biopsy, x-rays and vertebral fractures and less on DXA. This is improving our understanding of bone physiology of rare diseases. For example, skeletal effects associated with transgender pharmacotherapy have not yet been widely studied. Issues include start-time for treatment, particularly with regard to puberty, and the short- and long-term effects from cross-sex therapies on mineral metabolism.

Looking to the future, a more holistic approach to musculoskeletal health is needed given the multifactorial, polygenic nature of osteoporosis, to facilitate healthy aging/frailty prevention.

### 6.1 Future Developments in 2D and 3D Imaging

To date, bone mass measured by DXA is the most widely used bone phenotypic measure for genetic population studies of osteoporosis, in large part due to the ubiquitous presence of DXA scanners, which are widely used clinically and in large scale population studies. More recent genetic studies have examined other related phenotypes including hip geometry and shape (270). Looking to the future, DXA scans are likely to contribute to genetic studies of other age-related musculoskeletal conditions. For example, a range of other phenotypes, more closely related to osteoarthritis, are currently being generated in hip and knee DXA scans from approximately 100,000 individuals from UK Biobank^4^. However, a broad number of new technical approaches are under development.

For mammalian and fish studies, an optical coherence tomography (OCT) and synchrotron radiation microcomputed tomography (SR-µCT) may be developed for future use. OCT provides non-invasive high-resolution three-dimensional (3D) images of biological tissue and quantification of chromophores in tissues (271). Fish and human bones contain hydroxyapatite crystals so it can be compared and quantification of BMD is possible to obtain by comparison to sample with known hydroxyapatite levels. Better resolution (100 nm) can be achieved with synchrotron equipped μCT technologies (SR-μCT) with better assessing of bone micro-architecture (11).

Among recently developed MRI techniques is ultrashort echo time (UTE) MRI. Clinical MRI cannot detect water bound to organic matrix, or the free water in the pores of the Haversian system of cortical bone due to the very short apparent transverse relaxation times (T2 *). Therefore, a new class of sequences, ultrashort-TE (UTE) sequences have been developed recently, with TEs of less than 100 µs. This is much shorter than TEs of conventional sequences. These sequences can be used to detect water signals from within cortical bone (272, 273).

PET scanning can be used for some analyses in bone, and has been used in animal models to assess changes in e.g. bone vascularity or stress fractures (274). However, the reduction of radiation and dose exposures are critical in medical and research imaging, since high doses of radiation are associated with DNA damage. Multiple researchers are actively engaged in the development of clinical total-body PET hardware, promising improvements in dose reductions, reduced scan times, and quantitative kinetic modelling capabilities (275).

Artificial intelligence as new methods for automatic image segmentation, and prediction of fracture risk shows promising clinical value (276). Advances in artificial intelligence (deep learning) also perform well in classifying skeletal radiographs (277). Drug and genetic screening as well as longitudinal studies in zebrafish would benefit from AI, towards implementing novel platforms for gene functional validation through rapid skeletal phenotypic assessment.

### 6.2 Contribution of -omics Technologies to the Phenotypic Dissection of Musculoskeletal Traits

Historically, genome-wide association studies (GWAS) and next generation sequencing have revolutionized genetic diagnostic services and our understanding of common and rare bone diseases. For example, there are now 20+ genes identified that cause osteogenesis imperfecta through whole genome, exome and RNA sequencing and thousands of genetic variants arising from meta-analyses of the Genetic Factors of Osteoporosis (GEFOS) consortium, and the UK Biobank.

Nevertheless, several steps are needed before GWAS discoveries can be translated to biologic processes underlying the genotype-phenotype relationship. First, variants identified by GWAS need to be linked to the gene(s) in the region. Second, such target genes [identified through GWAS or whole exome sequencing (WES)] need to be placed in the context of pathways affected by the genetic variation. Third, functional, mechanistic studies need to establish how the given alteration of the biologic pathway(s) results in a phenotype.

It is here, where the confluence of a roadmap of gene functional evaluations and a detailed assessment of the laboratory and musculoskeletal phenotype can provide mechanistic insight into the relevant biological processes underlying disease. Multi-omics approaches are used to identify laboratory, phenotypic, disease-specific signatures.

Here we provide a succinct overview of multi-omics layers and their importance for bone phenotyping:

Genomics and Epigenomics: Gene coding regions (underlying most Mendelian disorders) make up less than 3% of the human genome while, approximately 90% of the single-nucleotide polymorphisms (SNPs) that are associated with human disease lie within intergenic or intronic regions. As such, genetic variation (polymorphisms, insertion/deletions and mutations) in intergenic regions, such as enhancers, can strongly affect gene expression, demonstrating a tight regulatory network between the coding and noncoding parts of the genome.

Epigenetic changes at DNA level include methylation, where methyl groups are added to the DNA molecule and change DNA activity, e.g. the repression of gene transcription without change of the DNA sequence. Important examples for these effects include genomic imprinting, X-chromosome inactivation, or repression of transposable elements. Effects on bone have been described in ageing (278) and oncology (279), but also increasingly during metabolic challenges, such as chronic kidney disease (280).

Another important epigenetic modification among others is considered histone tail modifications, where covalent post-translational modification (PTM) of nuclear histone proteins occur *via* methylation, phosphorylation, acetylation, ubiquitination, crotonisation, or sumoylation processes. These changes are currently addressed in a number of bone fields, such as bone and cancer as well as inflammation and rheumatic diseases (281). As an example, for bone/vasculature calcification interactions, a crosstalk between osteogenic transcription factors and histone deacetylases has been described. The inhibition/activation of histone deacetylases might help to develop potential therapeutic interventions in future (282).

For more specific information, see also the publication of GEMSTONE WG3 “Gene & Therapeutic Discoveries in Bone Mass Disorders”.

Transcriptomics: RNA Sequencing, gene expression, eQTLs, Non-coding RNAs (lncRNA and miRNA)

Non-coding RNAs (ncRNAs) are recent candidates to become future diagnostic bone biomarkers (283). ncRNA transcripts vary in length from around 22 nucleotides for microRNAs (miRNAs) to more than 200 nucleotides for long non-coding RNAs (lncRNAs). They are found in tissues, but importantly also in body fluids, where they are easier accessible for detection (283, 284).

Different types of ncRNAs are involved in several processes like DNA replication (285, 286), translation (287), RNA splicing (288) and transcriptional regulation (289). Especially miRNAs and lncRNAs are in focus as biomarkers for many conditions, including osteoporosis with some commercially available assays (290–292). They are stable in the bloodstream and protected from RNAse digestion. In the exosomal fraction of body fluids, both miRNAs and lncRNAs are enriched, indicating potentially active secretion of these RNA species from their cells of origin (293, 294).

miRNAs are best studied for their involvement in the control of bone formation and homeostasis through their regulatory functions in osteoblast and osteoclast development (295), not only in metabolic bone diseases, but also in case of cancer and bone metastases. Currently, ncRNA assays are not routinely used and require in most cases a dedicated lab with established PCR procedures. However, ncRNAs may help in the multi-omics phenotype characterization of rare bone diseases and defining disease state in common bone diseases.

Proteomics and metabolomics: The protein contents, i.e. the proteomes, of tissues and cells logically occupy a central position within the biologic processes underlying genotype-phenotype relationships. Proteomes can be studied qualitatively and/or quantitatively at a large scale by proteomics (296). Nowadays, mostly based on the use of liquid chromatography and mass spectroscopy methodologies, proteomic approaches can be employed for protein identification and quantification in samples as diverse as tissues, blood and cells, to provide a comprehensive and quantitative information on their proteomes (296). Therefore, a closer inspection of the bone proteome by proteomics is surely a fundamental tool to put in place towards the phenotypic dissection of musculoskeletal traits. For instance, proteomic approaches can help detect changes in the signal transduction of bone cells, in the regulatory mechanisms that govern bone cell differentiation, among other cellular and tissue processes enrolled in bone metabolism in both physiologic and pathological contexts (297). Accordingly up to now, several studies have made important contributions to our knowledge of the bone proteome, as well as, of the proteomes of individual bone cells (reviewed in (297–299). A special emphasis has been given to identify proteomic changes in osteoporosis (300).

Modern metabolomic analysis (analytical chemistry and bioinformatics) is capable of detecting hundreds of metabolites in human serum and hence identify novel biomarkers and biochemical signatures of disease. These topics are further described in the publication of GEMSTONE WG4.

Microbiomics: Animal studies using germ free mice, antibiotics, probiotics (i.e., microorganisms which confer a health benefit on the host) or prebiotics (i.e., nutrients capable to modify the gut microbiota) have shown that the complex community of microbes colonizing the gastrointestinal tract may regulate bone mass (301, 302). The impact of major alterations of the gut microbiota has been evaluated using either germ-free mice raised in sterile isolators and completely devoid of microbiota, or rodents depleted of gut microbiota by antibiotic use. These rodent models may be inoculated with specific microbes or communities of microbes to examine the effects they trigger on the skeleton in their host. All these studies using germ free mice or antibiotic treated mice exemplify extreme situations and it might be more physiological to look at the bone effects of treatments resulting in minor but specific changes of an already present gut microbiota. Probiotics and prebiotics given to rodents with an already present gut microbiota have been used to demonstrate that specific changes in the gut microbiota may protect against ovariectomy-induced and inflammation-induced bone loss (303). To characterize the skeletal phenotypes in these rodent bone loss models, standard analyses including DXA of areal BMD, MicroCT of cortical and trabecular bone parameters in the axial and appendicular skeleton, static and dynamic bone histomorphometry, bone strength estimates using three-point bending tests of long bones, *in vitro* studies of primary cultures of osteoblasts and osteoclasts precursors as well as analyses of circulating BTMs can be used (304).

The first promising findings of two randomized clinical probiotic treatment trials recently revealed that certain probiotic treatments had some bone sparing effects on DXA measurements of areal BMD at the lumbar spine (305) or by CT measurements of volumetric BMD of the distal tibia in humans (306).

The results from the first human cross-sectional association studies between the gut microbiota composition and bone related parameters have yielded conflicting results, most likely as some of the studies were underpowered and not adjusted for major confounders affecting the gut microbiota composition. Large-scale population-based studies assessing the association between the gut microbiome composition, as assessed by cost efficient 16S rRNA sequencing, and DXA-derived phenotypes adjusting for relevant confounders including lifestyle factors, diet and medications are underway. Furthermore, meta-genome wide association studies using state of the art sequence methodology in combination with other -omics platforms should be performed to characterize functional gut microbiota signatures associated with human bone health in detail. In addition, these analyses should not be restricted to DXA-derived areal BMD but include bone architecture and dimensions, specific cortical and trabecular bone parameters and incident fracture risk.

## Conclusion and the Triangulation of [Diagnostic] Evidence: The Path to Personalized Medicine

Understanding complex systems such as the skeleton requires the integration of multiple layers of evidence arising from a combination of analytical methods, in humans and animals, as outlined in this publication. Integration across many disciplines is required to solve outstanding questions and create a “deep phenotype” which accurately captures disease signatures. Key to this is translatability – whether across methodologies or species – and synchronization of efforts. The ‘triangulation of evidence’ (307) can overcome logistical and ethical constraints related to experimental design and speed the rate at which the reality of personalized medicine is attained. Looking to the future, there are several areas where the utility of animal models is obvious. For example, skeletal effects associated with transgender pharmacotherapy have not yet been widely studied. Issues include start-time for treatment, particularly with regard to puberty, and the short- and long-term effects from cross-sex therapies on mineral metabolism. Further, on a broader scale ‘duration of exposure’ to a given risk factor during the life course is of interest for skeletal health from an epigenetic perspective. In the young, damaging exposures have not yet accumulated, while with age, these contribute to accelerated biological aging. In conclusion, the idea of triangulation of evidence emerges as a solid way to weigh the robustness of each layer of evidence, but most importantly to gain insight from the integrated systems perspective. Similarly, the triangulation approach helps to overcome severe logistical and ethical constraints on experimental design potentially arising at each level. Once etiologic validity has been satisfactorily established across the different dimensions, the next challenge is to amalgamate these into a “deep phenotype”. This assembled deep phenotype will pave the road to find drug targets and clinical applications, ultimately charting the course toward personalized medicine approaches, for each of us.

## Author Contributions

IF and BO-P initiated and organised the manuscript. IF, FM, WH, and BO-P generated the figures for the manuscript. All authors contributed to the article and approved the submitted version.

## Funding

Funding was obtained from the GEMSTONE COST Action (CA18139). The Origins of Bone and Cartilage Disease Programme analysed the skeletal phenotypes of knockout mice generated by the International Mouse Phenotyping Consortium (IMPC) and was funded by a Welcome Trust Strategic Award (101123) to GRW and JHDB. IF is enrolled in the PhD program MOLIN at the Medical University of Graz, funded by the Austrian Science Fund (FWF). FM is funded by the Swedish Research Council (2018-02981), Greta and Johan Kock Foundation, the A. Påhlsson, A. Osterlund Foundation and H Järnhardt Foundations, King Gustav V 80 year fund, Swedish Rheumatism foundation, Skåne University Hospital Research Fund, Research and Development Council of Region Skåne.

## Conflict of Interest

The authors declare that the research was conducted in the absence of any commercial or financial relationships that could be construed as a potential conflict of interest.

## Publisher’s Note

All claims expressed in this article are solely those of the authors and do not necessarily represent those of their affiliated organizations, or those of the publisher, the editors and the reviewers. Any product that may be evaluated in this article, or claim that may be made by its manufacturer, is not guaranteed or endorsed by the publisher.

## Footnotes

1
https://www.sheffield.ac.uk/FRAX/.

2
https://www.garvan.org.au/bone-fracture-risk.

3
https://qfracture.org/.

4
https://www.ukbiobank.ac.uk/.

## References

[1] Sobacchi C Menale C Villa A. The RANKL-RANK Axis: A Bone to Thymus Round Trip. Front Immunol (2019) 10. doi: 10.3389/fimmu.2019.00629 PMC6450200 30984193

[2] Zaiss MM Jones RM Schett G Pacifici R. The Gut-Bone Axis: How Bacterial Metabolites Bridge the Distance. J Clin Invest (2019) 129:3018–28. doi: 10.1172/JCI128521 PMC6668676 31305265

[3] Behera J Ison J Tyagi SC Tyagi N. The Role of Gut Microbiota in Bone Homeostasis. Bone (2020) 135. doi: 10.1016/j.bone.2020.115317 PMC8457311 32169602

[4] Lems WF Raterman HG. Critical Issues and Current Challenges in Osteoporosis and Fracture Prevention. An Overview of Unmet Needs. Ther Adv Musculoskelet Dis (2017) 9(12):299–316. doi: 10.1177/1759720X17732562 29201155 PMC5700787

[5] Kan L. Animal Models of Bone Diseases-A. In: Animal Models for the Study of Human Disease. Elsevier Inc (2013). p. 353–90.

[6] Maynard RD Ackert-Bicknell CL. Mouse Models and Online Resources for Functional Analysis of Osteoporosis Genome-Wide Association Studies. Front Endocrinol (2019) 10:277. doi: 10.3389/fendo.2019.00277 PMC6515928 31133984

[7] Haffner-Luntzer M Kovtun A Rapp AE Ignatius A. Mouse Models in Bone Fracture Healing Research. Curr Mol Biol Rep (2016) 2(2):101–11. doi: 10.1007/s40610-016-0037-3

[8] Jilka RL. The Relevance of Mouse Models for Investigating Age-Related Bone Loss in Humans. Biol Sci Cite J as J Gerontol A Biol Sci Med Sci (2013) 68(10):1209–17. doi: 10.1093/gerona/glt046 PMC3779631 23689830

[9] Song AJ Palmiter RD. Detecting and Avoiding Problems When Using the Cre–lox System. Trends Genet (2018) 34:333–40. doi: 10.1016/j.tig.2017.12.008 PMC5910175 29336844

[10] Hsu PD Lander ES Zhang F. Development and Applications of CRISPR-Cas9 for Genome Engineering. Cell (2014) 157:1262–78. doi: 10.1016/j.cell.2014.05.010 PMC4343198 24906146

[11] Bergen DJM Kague E Hammond CL. Zebrafish as an Emerging Model for Osteoporosis: A Primary Testing Platform for Screening New Osteo-Active Compounds. Front Endocrinol (2019) 10:6. doi: 10.3389/fendo.2019.00006 PMC6361756 30761080

[12] Kwon RY Watson CJ Karasik D. Using Zebrafish to Study Skeletal Genomics. Bone (2019) 126:37–50. doi: 10.1016/j.bone.2019.02.009 30763636 PMC6626559

[13] Hur M Gistelinck CA Huber P Lee J Thompson MH Monstad-Rios AT. MicroCT-Based Phenomics in the Zebrafish Skeleton Reveals Virtues of Deep Phenotyping in a Distributed Organ System. Elife (2017) 6:e26014. doi: 10.7554/eLife.26014 28884682 PMC5606849

[14] Pardo-Martin C Allalou A Medina J Eimon PM Wahlby C Yanik MF. High-Throughput Hyperdimensional Vertebrate Phenotyping. Nat Commun (2013) 4(1):1–9. doi: 10.1038/ncomms2475 PMC3573763 23403568

[15] Monma Y Shimada Y Nakayama H Zang L Nishimura N Tanaka T. Aging-Associated Microstructural Deterioration of Vertebra in Zebrafish. Bone Rep (2019) 11:100215. doi: 10.1016/j.bonr.2019.100215 31388517 PMC6676153

[16] Hayes AJ Reynolds S Nowell MA Meakin LB Habicher J Ledin J. Spinal Deformity in Aged Zebrafish Is Accompanied by Degenerative Changes to Their Vertebrae That Resemble Osteoarthritis. Heymann D, Editor. PloS One (2013) 8(9):e75787. doi: 10.1371/journal.pone.0075787 24086633 PMC3782452

[17] Gilad S Meiri E Yogev Y Benjamin S Lebanony D Yerushalmi N. Serum MicroRNAs Are Promising Novel Biomarkers. Williams S, Editor. PloS One (2008) 3(9):e3148. doi: 10.1371/journal.pone.0003148 18773077 PMC2519789

[18] Tomecka MJ Ethiraj LP Sánchez LM Roehl HH Carney TJ. Clinical Pathologies of Bone Fracture Modelled in Zebrafish. Dis Model Mech (2019) 12(9):dmm037630. doi: 10.1242/dmm.037630 31383797 PMC6765199

[19] Mahamid J Sharir A Addadi L Weiner S. Amorphous Calcium Phosphate is a Major Component of the Forming Fin Bones of Zebrafish: Indications for an Amorphous Precursor Phase. Proc Natl Acad Sci USA (2008) 105(35):12748–53. doi: 10.1073/pnas.0803354105 PMC2529085 18753619

[20] Giovannone D Paul S Schindler S Arata C Farmer DT Patel P. Programmed Conversion of Hypertrophic Chondrocytes Into Osteoblasts and Marrow Adipocytes Within Zebrafish Bones. Elife (2019) 8:e42736. doi: 10.7554/eLife.42736 30785394 PMC6398980

[21] Heubel BP Bredesen CA Schilling TF Le Pabic P. Endochondral Growth Zone Pattern and Activity in the Zebrafish Pharyngeal Skeleton. Dev Dyn (2021) 250(1):74–87. doi: 10.1002/dvdy.241 32852849 PMC8284821

[22] Frost HM. Bone “Mass” and the “Mechanostat”: A Proposal. Anat Rec (1987) 219(1):1–9. doi: 10.1002/ar.1092190104 3688455

[23] Burkholder T Foltz C Karlsson E Linton CG Smith JM. Health Evaluation of Experimental Laboratory Mice. Curr Protoc Mouse Biol (2012) 2(2):145–65. doi: 10.1002/9780470942390.mo110217 PMC3399545 22822473

[24] Kishi S Slack BE Uchiyama J Zhdanova IV. Zebrafish as a Genetic Model in Biological and Behavioral Gerontology: Where Development Meets Aging in Vertebrates - A Mini-Review. Gerontology (2009) 55:430–41. doi: 10.1159/000228892 PMC2820570 19654474

[25] Salles JP. Bone Metabolism During Pregnancy. Ann Endocrinol (Paris) (2016) 77(2):163–8. doi: 10.1016/j.ando.2016.04.004 27157104

[26] Salari P Abdollahi M. The Influence of Pregnancy and Lactation on Maternal Bone Health: A Systematic Review. J Fam Reprod Heal (2014) 8(4):135–48. PMC4266784 25530765

[27] Ireland A Crozier SR Heazell AEP Ward KA Godfrey KM Inskip HM. Breech Presentation is Associated With Lower Bone Mass and Area: Findings From the Southampton Women’s Survey. Osteoporos Int (2018) 29(10):2275–81. doi: 10.1007/s00198-018-4626-2 PMC6173302 30003305

[28] Engberg E Koivusalo SB Huvinen E Viljakainen H. Bone Health in Women With a History of Gestational Diabetes or Obesity. Acta Obstet Gynecol Scand (2020) 99(4):477–87. doi: 10.1111/aogs.13778 31784976

[29] Hannam K Lawlor DA Tobias JH. Maternal Preeclampsia Is Associated With Reduced Adolescent Offspring Hip BMD in a UK Population-Based Birth Cohort. J Bone Miner Res (2015) 30(9):1684–91. doi: 10.1002/jbmr.2506 PMC4540657 25761963

[30] Cummings SR Nevitt MC Browner WS Stone K Fox KM Ensrud KE. Risk Factors for Hip Fracture in White Women. N Engl J Med (1995) 332(12):767–73. doi: 10.1056/NEJM199503233321202 7862179

[31] Siris ES Chen YT Abbott TA Barrett-Connor E Miller PD Wehren LE. Bone Mineral Density Thresholds for Pharmacological Intervention to Prevent Fractures. Arch Intern Med (2004) 164(10):1108–12. doi: 10.1001/archinte.164.10.1108 15159268

[32] Kanis JA Johnell O De Laet C Johansson H Oden A Delmas P. A Meta-Analysis of Previous Fracture and Subsequent Fracture Risk. Bone (2004) 35(2):375–82. doi: 10.1016/j.bone.2004.03.024 15268886

[33] Matcuk GR Mahanty SR Skalski MR Patel DB White EA Gottsegen CJ. Stress Fractures: Pathophysiology, Clinical Presentation, Imaging Features, and Treatment Options. Emergency Radiol (2016) 23:365–75. doi: 10.1007/s10140-016-1390-5 27002328

[34] Saunier J Chapurlat R. Stress Fracture in Athletes. Joint Bone Spine (2018) 85:307–10. doi: 10.1016/j.jbspin.2017.04.013 28512006

[35] Jager PL Jonkman S Koolhaas W Stiekema A Wolffenbuttel BHR Slart RHJA. Combined Vertebral Fracture Assessment and Bone Mineral Density Measurement: A New Standard in the Diagnosis of Osteoporosis in Academic Populations. Osteoporos Int (2011) 22(4):1059–68. doi: 10.1007/s00198-010-1293-3 PMC3046356 20571773

[36] Ganda K Puech M Chen JS Speerin R Bleasel J Center JR. Models of Care for the Secondary Prevention of Osteoporotic Fractures: A Systematic Review and Meta-Analysis. Osteoporosis Int (2013) 24:393–406. doi: 10.1007/s00198-012-2090-y 22829395

[37] Nilsson KH Henning P Shahawy M Nethander M Andersen TL Ejersted C. RSPO3 Is Important for Trabecular Bone and Fracture Risk in Mice and Humans. Nat Commun (2021) 12(1):4923. doi: 10.1038/s41467-021-25124-2 34389713 PMC8363747

[38] Kague E Roy P Asselin G Hu G Simonet J Stanley A. Osterix/Sp7 Limits Cranial Bone Initiation Sites and is Required for Formation of Sutures. Dev Biol (2016) 413(2):160–72. doi: 10.1016/j.ydbio.2016.03.011 PMC5469377 26992365

[39] Gistelinck C Kwon RY Malfait F Symoens S Harris MP Henke K. Zebrafish Type I Collagen Mutants Faithfully Recapitulate Human Type I Collagenopathies. Proc Natl Acad Sci USA (2018) 115(34):E8037–46. doi: 10.1073/pnas.1722200115 PMC6112716 30082390

[40] Fiedler IAK Schmidt FN Wölfel EM Plumeyer C Milovanovic P Gioia R. Severely Impaired Bone Material Quality in Chihuahua Zebrafish Resembles Classical Dominant Human Osteogenesis Imperfecta. J Bone Miner Res (2018) 33(8):1489–99. doi: 10.1002/jbmr.3445 29665086

[41] Bergquist R Weber M Schwenk M Ulseth S Helbostad JL Vereijken B. Performance-Based Clinical Tests of Balance and Muscle Strength Used in Young Seniors: A Systematic Literature Review. BMC Geriatr (2019) 19(1):9. doi: 10.1186/s12877-018-1011-0 30626340 PMC6327480

[42] Clarke KA Still J. Gait Analysis in the Mouse. Physiol Behav (1999) 66(5):723–9. doi: 10.1016/S0031-9384(98)00343-6 10405098

[43] Histing T Kristen A Roth C Holstein JH Garcia P Matthys R. *In Vivo* Gait Analysis in a Mouse Femur Fracture Model. J Biomech (2010) 43(16):3240–3. doi: 10.1016/j.jbiomech.2010.07.019 20832805

[44] Takeshita H Yamamoto K Nozato S Inagaki T Tsuchimochi H Shirai M. Modified Forelimb Grip Strength Test Detects Aging-Associated Physiological Decline in Skeletal Muscle Function in Male Mice. Sci Rep (2017) 7(1):1–9. doi: 10.1038/srep42323 28176863 PMC5296723

[45] Connolly AM Keeling RM Mehta S Pestronk A Sanes JR. Three Mouse Models of Muscular Dystrophy: The Natural History of Strength and Fatigue in Dystrophin-, Dystrophin/Utrophin-, and Laminin α2-Deficient Mice. Neuromuscul Disord (2001) 11(8):703–12. doi: 10.1016/S0960-8966(01)00232-2 11595512

[46] Justice JN Carter CS Beck HJ Gioscia-Ryan RA McQueen M Enoka RM. Battery of Behavioral Tests in Mice That Models Age-Associated Changes in Human Motor Function. Age (Omaha) (2014) 36(2):583–95. doi: 10.1007/s11357-013-9589-9 PMC4039275 24122289

[47] Brooks SP Dunnett SB. Tests to Assess Motor Phenotype in Mice: A User’s Guide. Nat Rev Neurosci (2009) 10:519–29. doi: 10.1038/nrn2652 19513088

[48] Matsumoto K Ishihara K Tanaka K Inoue K Fushiki T. An Adjustable-Current Swimming Pool for the Evaluation of Endurance Capacity of Mice. J Appl Physiol (1996) 81(4):1843–9. doi: 10.1152/jappl.1996.81.4.1843 8904607

[49] Nelson CE Hakim CH Ousterout DG Thakore PI Moreb EA Castellanos Rivera RM. *In Vivo* Genome Editing Improves Muscle Function in a Mouse Model of Duchenne Muscular Dystrophy. Science (2016) 351(6271):403–7. doi: 10.1126/science.aad5143 PMC4883596 26721684

[50] Suniaga S Rolvien T Vom Scheidt A Fiedler IAK Bale HA Huysseune A. Increased Mechanical Loading Through Controlled Swimming Exercise Induces Bone Formation and Mineralization in Adult Zebrafish. Sci Rep (2018) 8(1):3646. doi: 10.1038/s41598-018-21776-1 29483529 PMC5826918

[51] Leslie WD Morin SN. New Developments in Fracture Risk Assessment for Current Osteoporosis Reports. Curr Osteoporosis Rep (2020) 18:115–29. doi: 10.1007/s11914-020-00590-7 32285250

[52] Kanis JA Harvey NC Johansson H Liu E Vandenput L Lorentzon M. A Decade of FRAX: How has it Changed the Management of Osteoporosis? Aging Clin Exp Res (2020) 32:187–96. doi: 10.1007/s40520-019-01432-y 32043227

[53] Beaudoin C Moore L Gagné M Bessette L Ste-Marie LG Brown JP. Performance of Predictive Tools to Identify Individuals at Risk of non-Traumatic Fracture: A Systematic Review, Meta-Analysis, and Meta-Regression. Osteoporosis Int (2019) 30:721–40. doi: 10.1007/s00198-019-04919-6 30877348

[54] Searle SD Mitnitski A Gahbauer EA Gill TM Rockwood K. A Standard Procedure for Creating a Frailty Index. BMC Geriatr (2008) 8(1):24. doi: 10.1186/1471-2318-8-24 18826625 PMC2573877

[55] Rockwood K Howlett SE. Age-Related Deficit Accumulation and the Diseases of Ageing. Mech Ageing Dev (2019) 180:107–16. doi: 10.1016/j.mad.2019.04.005 31002924

[56] Kennedy CC Ioannidis G Rockwood K Thabane L Adachi JD Kirkland S. A Frailty Index Predicts 10-Year Fracture Risk in Adults Age 25 Years and Older: Results From the Canadian Multicentre Osteoporosis Study (CaMos). Osteoporos Int (2014) 25(12):2825–32. doi: 10.1007/s00198-014-2828-9 PMC5094886 25103215

[57] Bartosch P McGuigan FE Akesson KE. Progression of Frailty and Prevalence of Osteoporosis in a Community Cohort of Older Women—a 10-Year Longitudinal Study. Osteoporos Int (2018) 29(10):2191–9. doi: 10.1007/s00198-018-4593-7 PMC6154042 29947868

[58] Ward LM Weber DR Munns CF Högler W Zemel BS. A Contemporary View of the Definition and Diagnosis of Osteoporosis in Children and Adolescents. J Clin Endocrinol Metab (2020) 105:e2088–97. doi: 10.1210/clinem/dgz294 PMC7121121 31865390

[59] Messina C Maffi G Vitale JA Ulivieri FM Guglielmi G Sconfenza LM. Diagnostic Imaging of Osteoporosis and Sarcopenia: A Narrative Review. Quantitative Imaging Med Surgery (2018) 8:86–99. doi: 10.21037/qims.2018.01.01 PMC5835659 29541625

[60] Genant HK Wu CY van Kuijk C Nevitt MC. Vertebral Fracture Assessment Using a Semiquantitative Technique. J Bone Miner Res (1993) 8(9):1137–48. doi: 10.1002/jbmr.5650080915 8237484

[61] Schnake KJ Blattert TR Hahn P Franck A Hartmann F Ullrich B. Classification of Osteoporotic Thoracolumbar Spine Fractures: Recommendations of the Spine Section of the German Society for Orthopaedics and Trauma (DGOU). Glob Spine J (2018) 8(2 Suppl):46S–9S. doi: 10.1177/2192568217717972 PMC6130101 30210960

[62] Szulc P. Vertebral Fracture: Diagnostic Difficulties of a Major Medical Problem. J Bone Mineral Res (2018) 33:553–9. doi: 10.1002/jbmr.3404 29419882

[63] Fan YL Peh WCG. Radiology of Osteoporosis: Old and New Findings. Semin Musculoskelet Radiol (2016) 20(3):235–45. doi: 10.1055/s-0036-1592371 27741539

[64] Monsour PA Dudhia R. Implant Radiography and Radiology. Aust Dental J (2008) 53(Suppl 1):S11–25. doi: 10.1111/j.1834-7819.2008.00037.x 18498579

[65] Butterfield NC Logan JG Waung J Williams GR Bassett JHD. Quantitative X-Ray Imaging of Mouse Bone by Faxitron. Methods Mol Biol (2019) 1914:559–69. doi: 10.1007/978-1-4939-8997-3_30 30729486

[66] Bassett JHD Gogakos A White JK Evans H Jacques RM van der Spek AH. Rapid-Throughput Skeletal Phenotyping of 100 Knockout Mice Identifies 9 New Genes That Determine Bone Strength. PloS Genet (2012) 8(8):e1002858. doi: 10.1371/journal.pgen.1002858 22876197 PMC3410859

[67] Fisher S Jagadeeswaran P Halpern ME. Radiographic Analysis of Zebrafish Skeletal Defects. Dev Biol (2003) 264(1):64–76. doi: 10.1016/S0012-1606(03)00399-3 14623232

[68] Cummings SR Browner W Cummings SR Black DM Nevitt MC Browner W. Bone Density at Various Sites for Prediction of Hip Fractures. Lancet (1993) 341(8837):72–5. doi: 10.1016/0140-6736(93)92555-8 8093403

[69] Xi Z Mummaneni PV Wang M Ruan H Burch S Deviren V. The Association Between Lower Hounsfield Units on Computed Tomography and Cage Subsidence After Lateral Lumbar Interbody Fusion. Neurosurg Focus (2020) 49(2):1–8. doi: 10.3171/2020.5.FOCUS20169 32738801

[70] Dimai HP. Use of Dual-Energy X-Ray Absorptiometry (DXA) for Diagnosis and Fracture Risk Assessment; WHO-Criteria, T- and Z-Score, and Reference Databases. Bone (2017) 104:39–43. doi: 10.1016/j.bone.2016.12.016 28041872

[71] Johannesdottir F Thrall E Muller J Keaveny TM Kopperdahl DL Bouxsein ML. Comparison of non-Invasive Assessments of Strength of the Proximal Femur. Bone (2017) 105:93–102. doi: 10.1016/j.bone.2017.07.023 28739416

[72] Bishop N Arundel P Clark E Dimitri P Farr J Jones G. Fracture Prediction and the Definition of Osteoporosis in Children and Adolescents: The ISCD 2013 Pediatric Official Positions. J Clin Densitom (2014) 17(2):275–80. doi: 10.1016/j.jocd.2014.01.004 24631254

[73] Crabtree NJ Shaw NJ Bishop NJ Adams JE Mughal MZ Arundel P. Amalgamated Reference Data for Size-Adjusted Bone Densitometry Measurements in 3598 Children and Young Adults-The ALPHABET Study. J Bone Miner Res (2017) 32(1):172–80. doi: 10.1002/jbmr.2935 PMC5453244 27490028

[74] Weber DR Boyce A Gordon C Högler W Kecskemethy HH Misra M. The Utility of DXA Assessment at the Forearm, Proximal Femur, and Lateral Distal Femur, and Vertebral Fracture Assessment in the Pediatric Population: 2019 ISCD Official Position. J Clin Densitometry (2019) 22:567–89. doi: 10.1016/j.jocd.2019.07.002 PMC7010480 31421951

[75] Bonnick SL. HSA: Beyond BMD With DXA. Bone (2007) 41(1 SUPPL):S9–12. doi: 10.1016/j.bone.2007.03.007 17459802

[76] Cha YH Yoo J. Comparison of Hip Structure Analysis and Grip Strength Between Femoral Neck and Basicervical Fractures. BMC Musculoskelet Disord (2021) 22(1):461. doi: 10.1186/s12891-021-04363-w 34011356 PMC8135173

[77] Kaptoge S Beck TJ Reeve J Stone KL Hillier TA Cauley JA. Prediction of Incident Hip Fracture Risk by Femur Geometry Variables Measured by Hip Structural Analysis in the Study of Osteoporotic Fractures. J Bone Miner Res (2008) 23(12):1892–904. doi: 10.1359/jbmr.080802 PMC2686919 18684092

[78] Leslie WD Luo Y Yang S Goertzen AL Ahmed S Delubac I. Fracture Risk Indices From DXA-Based Finite Element Analysis Predict Incident Fractures Independently From FRAX: The Manitoba BMD Registry. J Clin Densitom (2019) 22(3):338–45. doi: 10.1016/j.jocd.2019.02.001 30852033

[79] Ponti F Santoro A Mercatelli D Gasperini C Conte M Martucci M. Aging and Imaging Assessment of Body Composition: From Fat to Facts. Front Endocrinol (2020) 10:861. doi: 10.3389/fendo.2019.00861 PMC6970947 31993018

[80] Busse B Hahn M Soltau M Zustin J Püschel K Duda GN. Increased Calcium Content and Inhomogeneity of Mineralization Render Bone Toughness in Osteoporosis: Mineralization, Morphology and Biomechanics of Human Single Trabeculae. Bone (2009) 45(6):1034–43. doi: 10.1016/j.bone.2009.08.002 19679206

[81] Shi J Lee S Uyeda M Tanjaya J Kim JK Pan HC. Guidelines for Dual Energy X-Ray Absorptiometry Analysis of Trabecular Bone-Rich Regions in Mice: Improved Precision, Accuracy, and Sensitivity for Assessing Longitudinal Bone Changes. Tissue Eng Part C Methods (2016) 22(5):451–63. doi: 10.1089/ten.tec.2015.0383 PMC4870654 26956416

[82] Nagy TR Clair AL. Precision and Accuracy of Dual-Energy X-Ray Absorptiometry for Determining *In Vivo* Body Composition of Mice. Obes Res (2000) 8(5):392–8. doi: 10.1038/oby.2000.47 10968731

[83] Wright LE Christian PJ Rivera Z Van Alstine WG L Funk J L Bouxsein M. Comparison of Skeletal Effects of Ovariectomy Versus Chemically Induced Ovarian Failure in Mice. J Bone Miner Res (2008) 23(8):1296–303. doi: 10.1359/jbmr.080309 PMC3276352 18348702

[84] Chou SH LeBoff MS. Vertebral Imaging in the Diagnosis of Osteoporosis: A Clinician’s Perspective. Curr Osteoporosis Rep (2017) 15:509–20. doi: 10.1007/s11914-017-0404-x 29103097

[85] Adult Positions - ISCD. Available at: https://iscd.org/learn/official-positions/adult-positions/.

[86] Lewiecki EM Gordon CM Baim S Binkley N Bilezikian JP Kendler DL. Special Report on the 2007 Adult and Pediatric Position Development Conferences of the International Society for Clinical Densitometry. Osteoporos Int (2008) 19(10):1369–78. doi: 10.1007/s00198-008-0689-9 18633664

[87] Cosman F de Beur SJ LeBoff MS Lewiecki EM Tanner B Randall S. Clinician’s Guide to Prevention and Treatment of Osteoporosis. Osteoporos Int (2014) 25(10):2359–81. doi: 10.1007/s00198-014-2794-2 PMC4176573 25182228

[88] Pediatric Positions - ISCD. Available at: https://iscd.org/learn/official-positions/pediatric-positions/.

[89] Newham E Kague E Aggleton JA Fernee C Brown KR Hammond CL. Finite Element and Deformation Analyses Predict Pattern of Bone Failure in Loaded Zebrafish Spines. J R Soc Interface (2019) 16(160):20190430. doi: 10.1098/rsif.2019.0430 31690186 PMC6893493

[90] Sousa S Valerio F Jacinto A. A New Zebrafish Bone Crush Injury Model. Biol Open (2012) 1(9):915–21. doi: 10.1242/bio.2012877 PMC3507236 23213486

[91] Lerchbaum E Trummer C Theiler-Schwetz V Kollmann M Wölfler M Pilz S. Effects of Vitamin D Supplementation on Bone Turnover and Bone Mineral Density in Healthy Men: A Post-Hoc Analysis of a Randomized Controlled Trial. Nutrients (2019) 11(4):731. doi: 10.3390/nu11040731 PMC6521067 30934881

[92] Mirzaali MJ Libonati F Ferrario D Rinaudo L Messina C Ulivieri FM. Determinants of Bone Damage: An Ex-Vivo Study on Porcine Vertebrae. Vashishth D, Editor. PloS One (2018) 13(8):e0202210. doi: 10.1371/journal.pone.0202210 30114229 PMC6095531

[93] Leslie WD Aubry-Rozier B Lamy O Hans D. TBS (Trabecular Bone Score) and Diabetes-Related Fracture Risk. J Clin Endocrinol Metab (2013) 98(2):602–9. doi: 10.1210/jc.2012-3118 23341489

[94] Richards C Hans D Leslie WD. Trabecular Bone Score (TBS) Predicts Fracture in Ankylosing Spondylitis: The Manitoba BMD Registry. J Clin Densitom (2020) 23(4):543–8. doi: 10.1016/j.jocd.2020.01.003 32094033

[95] Shevroja E Aubry-Rozier B Hans G Gonzalez-Rodriguez E Stoll D Lamy O. Clinical Performance of the Updated Trabecular Bone Score (TBS) Algorithm, Which Accounts for the Soft Tissue Thickness: The OsteoLaus Study. J Bone Miner Res (2019) 34(12):2229–37. doi: 10.1002/jbmr.3851 31419331

[96] Guagnelli MA Winzenrieth R Lopez-Gonzalez D McClung MR Del Rio L Clark P. Bone Age as a Correction Factor for the Analysis of Trabecular Bone Score (TBS) in Children. Arch Osteoporos (2019) 14(1):1–7. doi: 10.1007/s11657-019-0573-6 30815747

[97] Jazinizadeh F Quenneville CE. Enhancing Hip Fracture Risk Prediction by Statistical Modeling and Texture Analysis on DXA Images. Med Eng Phys (2020) 78:14–20. doi: 10.1016/j.medengphy.2020.01.015 32057626

[98] vom Scheidt A Grisolia Seifert EF Pokrant C Püschel K Amling M Busse B. Subregional Areal Bone Mineral Density (aBMD) is a Better Predictor of Heterogeneity in Trabecular Microstructure of Vertebrae in Young and Aged Women Than Subregional Trabecular Bone Score (TBS). Bone (2019) 122:156–65. doi: 10.1016/j.bone.2019.02.014 30776500

[99] Glüer CC. Quantitative Ultrasound Techniques for the Assessment of Osteoporosis: Expert Agreement on Current Status. J Bone Miner Res (1997) 12(8):1280–8. doi: 10.1359/jbmr.1997.12.8.1280 9258759

[100] Hans D Dargent-Molina P Schott AM Sebert JL Cormier C Kotzki PO. Ulrasonographic Heel Measurements to Predict Hip Fracture in Elderly Women: The EPIDOS Prospective Study. Lancet (1996) 348(9026):511–4. doi: 10.1016/S0140-6736(95)11456-4 8757153

[101] Knapp KM. Quantitative Ultrasound and Bone Health. Salud Publica Mex (2009) 51(SUPPL.1):S18–24. doi: 10.1590/S0036-36342009000700005 19287888

[102] Kemp JP Morris JA Medina-Gomez C Forgetta V Warrington NM Youlten SE. Identification of 153 New Loci Associated With Heel Bone Mineral Density and Functional Involvement of GPC6 in Osteoporosis. Nat Genet (2017) 49(10):1468–75. doi: 10.1038/ng.3949 PMC5621629 28869591

[103] Ackermann O Simanowski J Eckert K. Fracture Ultrasound of the Extremities. Ultraschall der Medizin (2020) 41(1):12–28. doi: 10.1055/a-1023-1782 32023628

[104] Kanis JA Melton LJ Christiansen C Johnston CC Khaltaev N. The Diagnosis of Osteoporosis. J Bone Miner Res (1994) 9(8):1137–41. doi: 10.1002/jbmr.5650090802 7976495

[105] Lentle BC Hammond I Firth GB Sutton RAL. Imaging of Osteoporotic Fractures on XR, CT, and MR. Curr Radiol Rep (2014) 2:1–9. doi: 10.1007/s40134-013-0032-x

[106] O’Ryan FS Khoury S Liao W Han MM Hui RL Baer D. Intravenous Bisphosphonate-Related Osteonecrosis of the Jaw: Bone Scintigraphy as an Early Indicator. J Oral Maxillofac Surg (2009) 67(7):1363–72. doi: 10.1016/j.joms.2009.03.005 19531404

[107] Grüneboom A Kling L Christiansen S Mill L Maier A Engelke K. Next-Generation Imaging of the Skeletal System and its Blood Supply. Nat Rev Rheumatol (2019) 15:533–49. doi: 10.1038/s41584-019-0274-y 31395974

[108] Huang K Feng Y Liu D Liang W Li L. Quantification Evaluation of 99mtc-MDP Concentration in the Lumbar Spine With SPECT/CT: Compare With Bone Mineral Density. Ann Nucl Med (2020) 34(2):136–43. doi: 10.1007/s12149-019-01425-x 31768820

[109] Li YB Zheng X Wang R Wu H Han S Deng ZY. SPECT-CT Versus MRI in Localizing Active Lesions in Patients With Osteoporotic Vertebral Compression Fractures. Nucl Med Commun (2018) 39(7):610–7. doi: 10.1097/MNM.0000000000000857 29893749

[110] Butterfield NC Logan JG Waung J Williams GR Bassett JHD. Quantitative X-Ray Imaging of Mouse Bone by Faxitron. In: Methods in Molecular Biology. New York, NY: Humana Press (2019). p. 559–69. 10.1007/978-1-4939-8997-3_30 30729486

[111] Khalil MM Tremoleda JL Bayomy TB Gsell W. Molecular SPECT Imaging: An Overview. Int J Mol Imaging (2011) 2011:1–15. doi: 10.1155/2011/796025 PMC3094893 21603240

[112] Tremoleda JL Khalil M Gompels LL Wylezinska-Arridge M Vincent T Gsell W. Imaging Technologies for Preclinical Models of Bone and Joint Disorders. EJNMMI Res (2011) 1(1):1–14. doi: 10.1186/2191-219X-1-11 PMC3251252 22214535

[113] Lienemann PS Metzger S Kiveliö AS Blanc A Papageorgiou P Astolfo A. Longitudinal *In Vivo* Evaluation of Bone Regeneration by Combined Measurement of Multi-Pinhole SPECT and Micro-CT for Tissue Engineering. Sci Rep (2015) 5:10238. doi: 10.1038/srep10238 25989250 PMC4437296

[114] Adams JE. Quantitative Computed Tomography. Eur J Radiol (2009) 71(3):415–24. doi: 10.1016/j.ejrad.2009.04.074 19682815

[115] Lang TF. Quantitative Computed Tomography. Radiologic Clinics North America (2010) 48:589–600. doi: 10.1016/j.rcl.2010.03.001 20609894

[116] Engelke K van Rietbergen B Zysset P. FEA to Measure Bone Strength: A Review. Clin Rev Bone Mineral Metab (2016) 14:26–37. doi: 10.1007/s12018-015-9201-1

[117] Johannesdottir F Allaire B Bouxsein ML. Fracture Prediction by Computed Tomography and Finite Element Analysis: Current and Future Perspectives. Curr Osteoporosis Rep (2018) 16(4):411–22. doi: 10.1007/s11914-018-0450-z 29846870

[118] Lengsfeld M Schmitt J Alter P Kaminsky J Leppek R. Comparison of Geometry-Based and CT Voxel-Based Finite Element Modelling and Experimental Validation. Med Eng Phys (1998) 20(7):515–22. doi: 10.1016/S1350-4533(98)00054-X 9832027

[119] Zysset PK Dall’Ara E Varga P Pahr DH. Finite Element Analysis for Prediction of Bone Strength. Bonekey Rep (2013) 2:386. doi: 10.1038/bonekey.2013.120 24422106 PMC3765052

[120] Viceconti M. Predicting Bone Strength From CT Data: Clinical Applications. Morphologie (2019) 103(343):180–6. doi: 10.1016/j.morpho.2019.09.007 31630964

[121] Keaveny TM. Biomechanical Computed Tomography-Noninvasive Bone Strength Analysis Using Clinical Computed Tomography Scans. Ann N Y Acad Sci (2010) 1192(1):57–65. doi: 10.1111/j.1749-6632.2009.05348.x 20392218

[122] Hoffmann U Massaro JM D’Agostino RB Sr Kathiresan S Fox CS O’Donnell CJ. Cardiovascular Event Prediction and Risk Reclassification by Coronary, Aortic, and Valvular Calcification in the Framingham Heart Study. J Am Heart Assoc (2016) 5(2):e003144. doi: 10.1161/JAHA.115.003144 26903006 PMC4802453

[123] Ryan TM Shaw CN. Trabecular Bone Microstructure Scales Allometrically in the Primate Humerus and Femur. Proc R Soc B Biol Sci (2013) 280(1758):20130172. doi: 10.1098/rspb.2013.0172 PMC3619467 23486443

[124] Stagi S Cavalli L Cavalli T De Martino M Brandi ML. Peripheral Quantitative Computed Tomography (pQCT) for the Assessment of Bone Strength in Most of Bone Affecting Conditions in Developmental Age: A Review. Ital J Pediatrics (2016) 42:1–20. doi: 10.1186/s13052-016-0297-9 PMC5037897 27670687

[125] Paternoster L Lorentzon M Vandenput L Karlsson MK Ljunggren Ö Kindmark A. Genome-Wide Association Meta-Analysis of Cortical Bone Mineral Density Unravels Allelic Heterogeneity at the RANKL Locus and Potential Pleiotropic Effects on Bone. Gibson G, Editor. PloS Genet (2010) 6(11):e1001217. doi: 10.1371/journal.pgen.1001217 21124946 PMC2987837

[126] Paternoster L Lorentzon M Lehtimäki T Eriksson J Kähönen M Raitakari O. Genetic Determinants of Trabecular and Cortical Volumetric Bone Mineral Densities and Bone Microstructure. Richards JB, Editor. PloS Genet (2013) 9(2):e1003247. doi: 10.1371/journal.pgen.1003247 23437003 PMC3578773

[127] Zheng H-F Tobias JH Duncan E Evans DM Eriksson J Paternoster L. WNT16 Influences Bone Mineral Density, Cortical Bone Thickness, Bone Strength, and Osteoporotic Fracture Risk. Gibson G, Editor. PloS Genet (2012) 8(7):e1002745. doi: 10.1371/journal.pgen.1002745 22792071 PMC3390364

[128] Compston J. Type 2 Diabetes Mellitus and Bone. J Intern Med (2018) 283(2):140–53. doi: 10.1111/joim.12725 29265670

[129] Gasser JA Willnecker J. Bone Measurements by Peripheral Quantitative Computed Tomography in Rodents. Methods Mol Biol (2019) 1914:533–58. doi: 10.1007/978-1-4939-8997-3_29 30729485

[130] Schmidt C Priemel M Kohler T Weusten A Müller R Amling M. Precision and Accuracy of Peripheral Quantitative Computed Tomography (pQCT) in the Mouse Skeleton Compared With Histology and Microcomputed Tomography (μct). J Bone Miner Res (2003) 18(8):1486–96. doi: 10.1359/jbmr.2003.18.8.1486 12929938

[131] Brodt MD Pelz GB Taniguchi J Silva MJ. Accuracy of Peripheral Quantitative Computed Tomography (pQCT) for Assessing Area and Density of Mouse Cortical Bone. Calcif Tissue Int (2003) 73(4):411–8. doi: 10.1007/s00223-002-0006-0 14743831

[132] Manske SL Zhu Y Sandino C Boyd SK. Human Trabecular Bone Microarchitecture can be Assessed Independently of Density With Second Generation HR-pQCT. Bone (2015) 79:213–21. doi: 10.1016/j.bone.2015.06.006 26079995

[133] Whittier DE Boyd SK Burghardt AJ Paccou J Ghasem-Zadeh A Chapurlat R. Guidelines for the Assessment of Bone Density and Microarchitecture *In Vivo* Using High-Resolution Peripheral Quantitative Computed Tomography. Osteoporos Int (2020) 31(9):1607–27. doi: 10.1007/s00198-020-05438-5 PMC7429313 32458029

[134] Boutroy S Bouxsein ML Munoz F Delmas PD. *In Vivo* Assessment of Trabecular Bone Microarchitecture by High-Resolution Peripheral Quantitative Computed Tomography. J Clin Endocrinol Metab (2005) 90(12):6508–15. doi: 10.1210/jc.2005-1258 16189253

[135] Laib A Hauselmann HJ Ruegsegger P. *In Vivo* High Resolution 3D-QCT of the Human Forearm. Technol Heal Care (1998) 6(5–6):329–37. doi: 10.3233/THC-1998-65-606 10100936

[136] Shanbhogue VV Brixen K Hansen S. Age- and Sex-Related Changes in Bone Microarchitecture and Estimated Strength: A Three-Year Prospective Study Using HRpQCT. J Bone Miner Res (2016) 31(8):1541–9. doi: 10.1002/jbmr.2817 26896351

[137] Hamilton EJ Ghasem-Zadeh A Gianatti E Lim-Joon D Bolton D Zebaze R. Structural Decay of Bone Microarchitecture in Men With Prostate Cancer Treated With Androgen Deprivation Therapy. J Clin Endocrinol Metab (2010) 95(12):E456–63. doi: 10.1210/jc.2010-0902 20881261

[138] van Rietbergen B Ito K. A Survey of Micro-Finite Element Analysis for Clinical Assessment of Bone Strength: The First Decade. J Biomech (2015) 48(5):832–41. doi: 10.1016/j.jbiomech.2014.12.024 25553670

[139] Whittier DE Manske SL Kiel DP Bouxsein M Boyd SK. Harmonizing Finite Element Modelling for non-Invasive Strength Estimation by High-Resolution Peripheral Quantitative Computed Tomography. J Biomech (2018) 80:63–71. doi: 10.1016/j.jbiomech.2018.08.030 30201250 PMC6188787

[140] Kroker A Zhu Y Manske SL Barber R Mohtadi N Boyd SK. Quantitative *In Vivo* Assessment of Bone Microarchitecture in the Human Knee Using HR-pQCT. Bone (2017) 97:43–8. doi: 10.1016/j.bone.2016.12.015 28039095

[141] Sada K Chiba K Kajiyama S Okazaki N Yonekura A Tomita M. Bone Mineral Density and Microstructure of the Elbow in Baseball Pitchers: An Analysis by Second-Generation HR-pQCT. J Clin Densitom (2020) 23(2):322–8. doi: 10.1016/j.jocd.2019.03.001 31006601

[142] Zebaze R Seeman E. Cortical Bone: A Challenging Geography. J Bone Miner Res (2015) 30(1):24–9. doi: 10.1002/jbmr.2419 25431247

[143] Nishiyama KK Macdonald HM Buie HR Hanley DA Boyd SK. Postmenopausal Women With Osteopenia Have Higher Cortical Porosity and Thinner Cortices at the Distal Radius and Tibia Than Women With Normal aBMD: An *In Vivo* HR-pQCT Study. J Bone Miner Res (2009) 25(4):091019190442039–30. doi: 10.1359/jbmr.091020 19839766

[144] Burghardt AJ Buie HR Laib A Majumdar S Boyd SK. Reproducibility of Direct Quantitative Measures of Cortical Bone Microarchitecture of the Distal Radius and Tibia by HR-pQCT. Bone (2010) 47(3):519–28. doi: 10.1016/j.bone.2010.05.034 PMC2926164 20561906

[145] Zebaze R Ghasem-Zadeh A Mbala A Seeman E. A New Method of Segmentation of Compact-Appearing, Transitional and Trabecular Compartments and Quantification of Cortical Porosity From High Resolution Peripheral Quantitative Computed Tomographic Images. Bone (2013) 54(1):8–20. doi: 10.1016/j.bone.2013.01.007 23334082

[146] Burghardt AJ Pialat JB Kazakia GJ Boutroy S Engelke K Patsch JM. Multicenter Precision of Cortical and Trabecular Bone Quality Measures Assessed by High-Resolution Peripheral Quantitative Computed Tomography. J Bone Miner Res (2013) 28(3):524–36. doi: 10.1002/jbmr.1795 PMC3577969 23074145

[147] Piot A Chapurlat RD Claustrat B Szulc P. Relationship Between Sex Steroids and Deterioration of Bone Microarchitecture in Older Men: The Prospective STRAMBO Study. J Bone Miner Res (2019) 34(9):1562–73. doi: 10.1002/jbmr.3746 30995347

[148] Samelson EJ Broe KE Xu H Yang L Boyd S Biver E. Cortical and Trabecular Bone Microarchitecture as an Independent Predictor of Incident Fracture Risk in Older Women and Men in the Bone Microarchitecture International Consortium (BoMIC): A Prospective Study. Lancet Diabetes Endocrinol (2019) 7(1):34–43. doi: 10.1016/S2213-8587(18)30308-5 30503163 PMC6354581

[149] Tsai JN Uihlein AV Burnett-Bowie SM Neer RM Derrico NP Lee H. Effects of Two Years of Teriparatide, Denosumab, or Both on Bone Microarchitecture and Strength (DATA-HRpQCT Study). J Clin Endocrinol Metab (2016) 101(5):2023–30. doi: 10.1210/jc.2016-1160 PMC4870854 26964731

[150] Feldkamp LA Goldstein SA Parfitt MA Jesion G Kleerekoper M. The Direct Examination of Three-Dimensional Bone Architecture *In Vitro* by Computed Tomography. J Bone Miner Res (1989) 4(1):3–11. doi: 10.1002/jbmr.5650040103 2718776

[151] Campbell GM Sophocleous A. Quantitative Analysis of Bone and Soft Tissue by Micro-Computed Tomography: Applications to Ex Vivo and In Vivo Studies. Bonekey Rep (2014) 3:564. doi: 10.1038/bonekey.2014.59 25184037 PMC4140449

[152] Ozan F Pekedis M Koyuncu Ş Altay T Yıldız H Kayalı C. Micro-Computed Tomography and Mechanical Evaluation of Trabecular Bone Structure in Osteopenic and Osteoporotic Fractures. J Orthop Surg (2017) 25(1):230949901769271. doi: 10.1177/2309499017692718 28215116

[153] Perilli E Parkinson IH Reynolds KJ. Micro-CT Examination of Human Bone: From Biopsies Towards the Entire Organ. Ann Ist Super Sanita (2012) 48(1):75–82. doi: 10.4415/ANN_12_01_13 22456020

[154] Jiang Y Zhao J Liao EY Dai RC Wu XP Genant HK. Application of Micro-Ct Assessment of 3-D Bone Microstructure in Preclinical and Clinical Studies. J Bone Mineral Metab (2005) 23:122–31. doi: 10.1007/BF03026336 15984427

[155] Burghardt AJ Link TM Majumdar S. High-Resolution Computed Tomography for Clinical Imaging of Bone Microarchitecture. Clin Orthopaedics Related Res (2011) 469(8):2179–93. doi: 10.1007/s11999-010-1766-x PMC3126972 21344275

[156] De Bournonville S Vangrunderbeeck S Kerckhofs G. Contrast-Enhanced microCT for Virtual 3D Anatomical Pathology of Biological Tissues: A Literature Review. Contrast Media Mol Imaging (2019) 2019:8617406. doi: 10.1155/2019/8617406 30944550 PMC6421764

[157] Tratwal J Labella R Bravenboer N Kerckhofs G Douni E Scheller EL. Reporting Guidelines, Review of Methodological Standards, and Challenges Toward Harmonization in Bone Marrow Adiposity Research. Report of the Methodologies Working Group of the International Bone Marrow Adiposity Society. Front Endocrinol (2020) 11:65. doi: 10.3389/fendo.2020.00065 PMC7059536 32180758

[158] Dall’Ara E Boudiffa M Taylor C Schug D Fiegle E Kennerley AJ. Longitudinal Imaging of the Ageing Mouse. Mech Ageing Dev (2016) 160:93–116. doi: 10.1016/j.mad.2016.08.001 27530773

[159] Bouxsein ML Boyd SK Christiansen BA Guldberg RE Jepsen KJ Müller R. Guidelines for Assessment of Bone Microstructure in Rodents Using Micro-Computed Tomography. J Bone Mineral Res (2010) 25:1468–86. doi: 10.1002/jbmr.141 20533309

[160] Leitch VD Brassill MJ Rahman S Butterfield NC Ma P Logan JG. PYY is a Negative Regulator of Bone Mass and Strength. Bone (2019) 127:427–35. doi: 10.1016/j.bone.2019.07.011 PMC6715792 31306808

[161] Oliviero S Giorgi M Laud PJ Dall’Ara E. Effect of Repeated *In Vivo* microCT Imaging on the Properties of the Mouse Tibia. PloS One (2019) 14(11):e0225127. doi: 10.1371/journal.pone.0225127 31751367 PMC6874075

[162] Nadel HR. SPECT/CT in Pediatric Patient Management. Eur J Nucl Med Mol Imaging (2014) 41(Suppl 1):S104–14. doi: 10.1007/s00259-014-2697-7 24554052

[163] Almeida M Laurent MR Dubois V Claessens F O’Brien CA Bouillon R. Estrogens and Androgens in Skeletal Physiology and Pathophysiology. Physiol Rev (2017) 97(1):135–87. doi: 10.1152/physrev.00033.2015 PMC5539371 27807202

[164] Charles JF Sury M Tsang K Urso K Henke K Huang Y. Utility of Quantitative Micro-Computed Tomographic Analysis in Zebrafish to Define Gene Function During Skeletogenesis. Bone (2017) 101:162–71. doi: 10.1016/j.bone.2017.05.001 PMC5512604 28476577

[165] Khajuria DK Kumar VB Gigi D Gedanken A Karasik D. Accelerated Bone Regeneration by Nitrogen-Doped Carbon Dots Functionalized With Hydroxyapatite Nanoparticles. ACS Appl Mater Interfaces (2018) 10(23):19373–85. doi: 10.1021/acsami.8b02792 29782148

[166] Main RP Lynch ME van der Meulen MCH. Load-Induced Changes in Bone Stiffness and Cancellous and Cortical Bone Mass Following Tibial Compression Diminish With Age in Female Mice. J Exp Biol (2014) 217(10):1775–83. doi: 10.1242/jeb.085522 PMC4020944 24577445

[167] Wang J Kazakia GJ Zhou B Shi XT Guo XE. Distinct Tissue Mineral Density in Plate- and Rod-Like Trabeculae of Human Trabecular Bone. J Bone Miner Res (2015) 30(9):1641–50. doi: 10.1002/jbmr.2498 PMC4540699 25736715

[168] Doherty AH Ghalambor CK Donahue SW. Evolutionary Physiology of Bone: Bone Metabolism in Changing Environments. Physiology (2015) 30(1):17–29. doi: 10.1152/physiol.00022.2014 25559152

[169] Gistelinck C Witten PE Huysseune A Symoens S Malfait F Larionova D. Loss of Type I Collagen Telopeptide Lysyl Hydroxylation Causes Musculoskeletal Abnormalities in a Zebrafish Model of Bruck Syndrome. J Bone Miner Res (2016) 31(11):1930–42. doi: 10.1002/jbmr.2977 PMC5364950 27541483

[170] Kague E Witten PE Soenens M Campos CL Lubiana T Fisher S. Zebrafish Sp7 Mutants Show Tooth Cycling Independent of Attachment, Eruption and Poor Differentiation of Teeth. Dev Biol (2018) 435(2):176–84. doi: 10.1016/j.ydbio.2018.01.021 29409769

[171] Lawrence EA Kague E Aggleton JA Harniman RL Roddy KA Hammond CL. The Mechanical Impact of *Col11a2* Loss on Joints; *Col11a2* Mutant Zebrafish Show Changes to Joint Development and Function, Which Leads to Early-Onset Osteoarthritis. Philos Trans R Soc B Biol Sci (2018) 373(1759):20170335. doi: 10.1098/rstb.2017.0335 PMC6158203 30249781

[172] Adams JE. Advances in Bone Imaging for Osteoporosis. Nat Rev Endocrinol (2013) 9(1):28–42. doi: 10.1038/nrendo.2012.217 23232496

[173] Shayganfar A Khodayi M Ebrahimian S Tabrizi Z. Quantitative Diagnosis of Osteoporosis Using Lumbar Spine Signal Intensity in Magnetic Resonance Imaging. Br J Radiol (2019) 92(1097):20180774. doi: 10.1259/bjr.20180774 30759992 PMC6580909

[174] Abbasi-Rad S Saligheh Rad H. Quantification of Human Cortical Bone Bound and Free Water in Vivo With Ultrashort Echo Time MR Imaging: A Model-Based Approach. Radiology (2017) 283(3):862–72. doi: 10.1148/radiol.2016160780 28051911

[175] Dixon WT. Simple Proton Spectroscopic Imaging. Radiology (1984) 153(1):189–94. doi: 10.1148/radiology.153.1.6089263 6089263

[176] Taha MA Manske SL Kristensen E Taiani JT Krawetz R Wu Y. Assessment of the Efficacy of MRI for Detection of Changes in Bone Morphology in a Mouse Model of Bone Injury. J Magn Reson Imaging (2013) 38(1):231–7. doi: 10.1002/jmri.23876 23125100

[177] Haffner-Luntzer M Müller-Graf F Matthys R Hägele Y Fischer V Jonas R. Evaluation of High-Resolution *In Vivo* MRI for Longitudinal Analysis of Endochondral Fracture Healing in Mice. Garcia Aznar JM, Editor. PloS One (2017) 12(3):e0174283. doi: 10.1371/journal.pone.0174283 28333972 PMC5363916

[178] Turnbull DH Mori S. MRI in Mouse Developmental Biology. NMR Biomed (2007) 20(3):265–74. doi: 10.1002/nbm.1146 PMC2694493 17451170

[179] Koth J Maguire ML McClymont D Diffley L Thornton VL Beech J. High-Resolution Magnetic Resonance Imaging of the Regenerating Adult Zebrafish Heart. Sci Rep (2017) 7(1):2917. doi: 10.1038/s41598-017-03050-y 28592901 PMC5462770

[180] Merrifield GD Mullin J Gallagher L Tucker C Jansen MA Denvir M. Rapid and Recoverable *In Vivo* Magnetic Resonance Imaging of the Adult Zebrafish at 7T. Magn Reson Imaging (2017) 37:9–15. doi: 10.1016/j.mri.2016.10.013 27751860 PMC5344283

[181] Recker RR Moreira CA. Bone Histomorphometry in Clinical Practice. (2018). pp. 310–318. doi: 10.1002/9781119266594.ch39

[182] Chappard D Baslé MF Legrand E Audran M. New Laboratory Tools in the Assessment of Bone Quality. Osteoporosis Int (2011) 22:2225–40. doi: 10.1007/s00198-011-1573-6 21347743

[183] Compston J. Bone Histomorphometry. In: Methods in Bone Biology. Boston, MA: Springer (2007). p. 177–97.

[184] Slyfield CR Tkachenko EV Wilson DL Hernandez CJ. Three-Dimensional Dynamic Bone Histomorphometry. J Bone Miner Res (2012) 27(2):486–95. doi: 10.1002/jbmr.553 PMC3288521 22028195

[185] Malhan D Muelke M Rosch S Schaefer AB Merboth F Weisweiler D. An Optimized Approach to Perform Bone Histomorphometry. Front Endocrinol (Lausanne) (2018) 9:666. doi: 10.3389/fendo.2018.00666 30519215 PMC6259258

[186] Dempster DW Compston JE Drezner MK Glorieux FH Kanis JA Malluche H. Standardized Nomenclature, Symbols, and Units for Bone Histomorphometry: A 2012 Update of the Report of the ASBMR Histomorphometry Nomenclature Committee. J Bone Mineral Res (2013) 28:2–17. doi: 10.1002/jbmr.1805 PMC3672237 23197339

[187] Parfitt AM Drezner MK Glorieux FH Kanis JA Malluche H Meunier PJ. Bone Histomorphometry: Standardization of Nomenclature, Symbols, and Units: Report of the Asbmr Histomorphometry Nomenclature Committee. J Bone Miner Res (1987) 2(6):595–610. doi: 10.1002/jbmr.5650020617 3455637

[188] Glorieux FH Travers R Taylor A Bowen JR Rauch F Norman M. Normative Data for Iliac Bone Histomorphometry in Growing Children. Bone (2000) 26(2):103–9. doi: 10.1016/S8756-3282(99)00257-4 10678403

[189] Rauch F Travers R Parfitt A Glorieux F. Static and Dynamic Bone Histomorphometry in Children With Osteogenesis Imperfecta. Bone (2000) 26(6):581–9. doi: 10.1016/S8756-3282(00)00269-6 10831929

[190] Amling M Pösl M Ritzel H Hahn M Vogel M Wening VJ. Architecture and Distribution of Cancellous Bone Yield Vertebral Fracture Clues. A Histomorphometric Analysis of the Complete Spinal Column From 40 Autopsy Specimens. Arch Orthop Trauma Surg (1996) 115(5):262–9. doi: 10.1007/BF00439050 8836458

[191] Erben RG Glösmann M. Histomorphometry in Rodents. Methods Mol Biol (2019) 1914:411–35. doi: 10.1007/978-1-4939-8997-3_24 30729480

[192] Bassett JHD Boyde A Howell PGT Bassett RH Galliford TM Archanco M. Optimal Bone Strength and Mineralization Requires the Type 2 Iodothyronine Deiodinase in Osteoblasts. Proc Natl Acad Sci USA (2010) 107(16):7604–9. doi: 10.1073/pnas.0911346107 PMC2867713 20368437

[193] Dion N Fortin A Ste-Marie L-G. Methods in Bone Histomorphometry for Animal Models. Osteoporos Res (2011) 37–43. doi: 10.1007/978-0-85729-293-3_4

[194] Jun Du S Frenkel V Zohar Y Kindschi G. Visualizing Normal and Defective Bone Development in Zebrafish Embryos Using the Fluorescent Chromophore Calcein. Dev Biol (2001) 238(2):239–46. doi: 10.1006/dbio.2001.0390 11784007

[195] Kimmel CB DeLaurier A Ullmann B Dowd J McFadden M. Modes of Developmental Outgrowth and Shaping of a Craniofacial Bone in Zebrafish. PloS One (2010) 5(3):e9475. doi: 10.1371/journal.pone.0009475 20221441 PMC2832765

[196] Recidoro AM Roof AC Schmitt M Worton LE Petrie T Strand N. Botulinum Toxin Induces Muscle Paralysis and Inhibits Bone Regeneration in Zebrafish. J Bone Miner Res (2014) 29(11):2346–56. doi: 10.1002/jbmr.2274 PMC5108653 24806738

[197] Inohaya K Takano Y Kudo A. The Teleost Intervertebral Region Acts as a Growth Center of the Centrum: *In Vivo* Visualization of Osteoblasts and Their Progenitors in Transgenic Fish. Dev Dyn (2007) 236(11):3031–46. doi: 10.1002/dvdy.21329 17907202

[198] Edsall SC Franz-Odendaal TA. A Quick Whole-Mount Staining Protocol for Bone Deposition and Resorption. Zebrafish (2010) 7(3):275–80. doi: 10.1089/zeb.2009.0641 20807038

[199] Tang P Xiong Q Ge W Zhang L. The Role of MicroRNAs in Osteoclasts and Osteoporosis. RNA Biol (2014) 11(11):1355–63. doi: 10.1080/15476286.2014.996462 PMC4615571 25692234

[200] Schilling TF Piotrowski T Grandel H Brand M Heisenberg CP Jiang YJ. Jaw and Branchial Arch Mutants in Zebrafish I: Branchial Arches. Development (1996) 123:329–44. doi: 10.1242/dev.123.1.329 9007253

[201] Piotrowski T Schilling TF Brand M Jiang YJ Heisenberg CP Beuchle D. Jaw and Branchial Arch Mutants in Zebrafish II: Anterior Arches and Cartilage Differentiation. Development (1996) 123:345–56. doi: 10.1242/dev.123.1.345 9007254

[202] DeLaurier A Frank Eames B Blanco-Sánchez B Peng G He X Swartz ME. Zebrafish Sp7:EGFP: A Transgenic for Studying Otic Vesicle Formation, Skeletogenesis, and Bone Regeneration. Genesis (2010) 48(8):505–11. doi: 10.1002/dvg.20639 PMC2926247 20506187

[203] Singh SP Holdway JE Poss KD. Regeneration of Amputated Zebrafish Fin Rays From *De Novo* Osteoblasts. Dev Cell (2012) 22(4):879–86. doi: 10.1016/j.devcel.2012.03.006 PMC3341140 22516203

[204] Sharif F De Bakker MA Richardson MK. Osteoclast-Like Cells in Early Zebrafish Embryos. Cell J (2014) 16(2):211–24. PMC4072079 24567948

[205] Kobayashi-Sun J Yamamori S Kondo M Kuroda J Ikegame M Suzuki N. Uptake of Osteoblast-Derived Extracellular Vesicles Promotes the Differentiation of Osteoclasts in the Zebrafish Scale. Commun Biol (2020) 3(1):190. doi: 10.1038/s42003-020-0925-1 32327701 PMC7181839

[206] Witten PE Hansen A Hall BK. Features of Mono- and Multinucleated Bone Resorbing Cells of the Zebrafish Danio Rerio and Their Contribution to Skeletal Development, Remodeling, and Growth. J Morphol (2001) 250(3):197–207. doi: 10.1002/jmor.1065 11746460

[207] Nyman JS Granke M Singleton RC Pharr GM. Tissue-Level Mechanical Properties of Bone Contributing to Fracture Risk. Curr Osteoporosis Rep (2016) 14:138–50. doi: 10.1007/s11914-016-0314-3 PMC4927361 27263108

[208] Allen MR McNerny EMB Organ JM Wallace JM. True Gold or Pyrite: A Review of Reference Point Indentation for Assessing Bone Mechanical Properties In Vivo. J Bone Miner Res (2015) 30:1539–50. doi: 10.1002/jbmr.2603 PMC4825864 26235703

[209] Herrera S Diez-Perez A. Clinical Experience With Microindentation *In Vivo* in Humans. Bone (2017) 95:175–82. doi: 10.1016/j.bone.2016.11.003 27840302

[210] Schoeb M Hamdy NAT Malgo F Winter EM Appelman-Dijkstra NM. Added Value of Impact Microindentation in the Evaluation of Bone Fragility: A Systematic Review of the Literature. Front Endocrinol (2020) 11. doi: 10.3389/fendo.2020.00015 PMC7020781 32117052

[211] Diez-Perez A Güerri R Nogues X Cáceres E Peñ MJ Mellibovsky L. Microindentation for *In Vivo* Measurement of Bone Tissue Mechanical Properties in Humans. J Bone Miner Res (2010) 25(8):1877–85. doi: 10.1002/jbmr.73 PMC3153354 20200991

[212] Bridges D Randall C Hansma PK. A New Device for Performing Reference Point Indentation Without a Reference Probe. Rev Sci Instrum (2012) 83(4):044301. doi: 10.1063/1.3693085 22559552 PMC3331866

[213] Diez-Perez A Bouxsein ML Eriksen EF Khosla S Nyman JS Papapoulos S. Technical Note: Recommendations for a Standard Procedure to Assess Cortical Bone at the Tissue-Level *In Vivo* Using Impact Microindentation. Bone Rep (2016) 5:181–5. doi: 10.1016/j.bonr.2016.07.004 PMC5152622 27975078

[214] Kennedy OD Lendhey M Mauer P Philip A Basta-Pljakic J Schaffler MB. Microdamage Induced by *In Vivo* Reference Point Indentation in Mice is Repaired by Osteocyte-Apoptosis Mediated Remodeling. Bone (2017) 95:192–8. doi: 10.1016/j.bone.2016.11.029 PMC5776007 27919734

[215] Williamson L Hayes A Hanson ED Pivonka P Sims NA Gooi JH. High Dose Dietary Vitamin D3 Increases Bone Mass and Strength in Mice. Bone Rep (2017) 6:44–50. doi: 10.1016/j.bonr.2017.02.001 28377981 PMC5365305

[216] Carriero A Bruse JL Oldknow KJ Millán JL Farquharson C Shefelbine SJ. Reference Point Indentation is Not Indicative of Whole Mouse Bone Measures of Stress Intensity Fracture Toughness. Bone (2014) 69:174–9. doi: 10.1016/j.bone.2014.09.020 PMC4228060 25280470

[217] Srisuwananukorn A Allen MR Brown DM Wallace JM Organ JM. *In Vivo* Reference Point Indentation Measurement Variability in Skeletally Mature Inbred Mice. Bonekey Rep (2015) 4:712. doi: 10.1038/bonekey.2015.81 26131362 PMC4478874

[218] Wang XM Cui FZ Ge J Zhang Y Ma C. Variation of Nanomechanical Properties of Bone by Gene Mutation in the Zebrafish. Biomaterials (2002) 23(23):4557–63. doi: 10.1016/S0142-9612(02)00201-6 12322976

[219] Zhang Y Cui FZ Wang XM Feng QL Zhu XD. Mechanical Properties of Skeletal Bone in Gene-Mutated Stöpseldtl28d and Wild-Type Zebrafish (Danio Rerio) Measured by Atomic Force Microscopy-Based Nanoindentation. Bone (2002) 30(4):541–6. doi: 10.1016/S8756-3282(02)00676-2 11934643

[220] Chang Z Chen PY Chuang YJ Akhtar R. Zebrafish as a Model to Study Bone Maturation: Nanoscale Structural and Mechanical Characterization of Age-Related Changes in the Zebrafish Vertebral Column. J Mech Behav BioMed Mater (2018) 84:54–63. doi: 10.1016/j.jmbbm.2018.05.004 29747057

[221] Fan ZF Smith P Rauch F Harris GF. Nanoindentation as a Means for Distinguishing Clinical Type of Osteogenesis Imperfecta. Compos Part B Eng (2007) 38(3):411–5. doi: 10.1016/j.compositesb.2006.08.006

[222] Roschger P Fratzl P Eschberger J Klaushofer K. Validation of Quantitative Backscattered Electron Imaging for the Measurement of Mineral Density Distribution in Human Bone Biopsies. Bone (1998) 23(4):319–26. doi: 10.1016/S8756-3282(98)00112-4 9763143

[223] Roschger P Paschalis EP Fratzl P Klaushofer K. Bone Mineralization Density Distribution in Health and Disease. Bone (2008) 42(3):456–66. doi: 10.1016/j.bone.2007.10.021 18096457

[224] Fratzl-Zelman N Roschger P Misof BM Pfeffer S Glorieux FH Klaushofer K. Normative Data on Mineralization Density Distribution in Iliac Bone Biopsies of Children, Adolescents and Young Adults. Bone (2009) 44(6):1043–8. doi: 10.1016/j.bone.2009.02.021 19268565

[225] Carden A Morris MD. Application of Vibrational Spectroscopy to the Study of Mineralized Tissues (Review). J BioMed Opt (2000) 5(3):259. doi: 10.1117/1.429994 10958610

[226] Unal M Akkus O. Raman Spectral Classification of Mineral- and Collagen-Bound Water’s Associations to Elastic and Post-Yield Mechanical Properties of Cortical Bone. Bone (2015) 81:315–26. doi: 10.1016/j.bone.2015.07.024 PMC4640992 26211992

[227] Shah FA Ruscsák K Palmquist A. 50 Years of Scanning Electron Microscopy of Bone—a Comprehensive Overview of the Important Discoveries Made and Insights Gained Into Bone Material Properties in Health, Disease, and Taphonomy. Bone Res (2019) 7:1–15. doi: 10.1038/s41413-019-0053-z 31123620 PMC6531483

[228] Akkiraju H Bonor J Nohe A. An Improved Immunostaining and Imaging Methodology to Determine Cell and Protein Distributions Within the Bone Environment. J Histochem Cytochem (2016) 64(3):168–78. doi: 10.1369/0022155415626765 PMC4810797 26718242

[229] Matos LL Trufelli DC de Matos MGL da Silva Pinhal S. Immunohistochemistry as an Important Tool in Biomarkers Detection and Clinical Practice. Biomark Insights (2010) 5:9–20. doi: 10.4137/BMI.S2185 20212918 PMC2832341

[230] Fedchenko N Reifenrath J. Different Approaches for Interpretation and Reporting of Immunohistochemistry Analysis Results in the Bone Tissue - A Review. Diagn Pathol (2014) 9:221. doi: 10.1186/s13000-014-0221-9 25432701 PMC4260254

[231] Malluche HH Mawad H Monier-Faugere MC. Bone Biopsy in Patients With Osteoporosis. Curr Osteoporosis Rep (2007) 5:146–52. doi: 10.1007/s11914-007-0009-x 18430388

[232] Yang J Pan H Mishina Y. Tissue Preparation and Immunostaining of Mouse Craniofacial Tissues and Undecalcified Bone. J Vis Exp (2019) 2019(147):e59113. doi: 10.3791/59113 PMC6996940 31132049

[233] McDonald MM Reagan MR Youlten SE Mohanty ST Seckinger A Terry RL. Inhibiting the Osteocyte-Specific Protein Sclerostin Increases Bone Mass and Fracture Resistance in Multiple Myeloma. Blood (2017) 129(26):3452–64. doi: 10.1182/blood-2017-03-773341 PMC5492093 28515094

[234] Oralová V Rosa JT Soenens M Bek JW Willaert A Witten PE. Beyond the Whole-Mount Phenotype: High-Resolution Imaging in Fluorescence-Based Applications on Zebrafish. Biol Open (2019) 8(5):bio042374. doi: 10.1242/bio.042374 31126903 PMC6550072

[235] Kague E Hughes SM Lawrence EA Cross S Martin-Silverstone E Hammond CL. Scleraxis Genes are Required for Normal Musculoskeletal Development and for Rib Growth and Mineralization in Zebrafish. FASEB J (2019) 33(8):9116–30. doi: 10.1096/fj.201802654RR PMC6662971 31100023

[236] Brunt LH Begg K Kague E Cross S Hammond CL. Wnt Signalling Controls the Response to Mechanical Loading During Zebrafish Joint Development. Dev (2017) 144(15):2798–809. doi: 10.1242/dev.153528 PMC5560048 28684625

[237] Paul S Schindler S Giovannone D de Millo Terrazzani A Mariani FV Crump JG. Ihha Induces Hybrid Cartilage-Bone Cells During Zebrafish Jawbone Regeneration. Dev (2016) 143(12):2066–76. doi: 10.1242/dev.131292 PMC4920169 27122168

[238] Lee J Vasikaran S. Current Recommendations for Laboratory Testing and Use of Bone Turnover Markers in Management of Osteoporosis. Ann Lab Med (2012) 32:105–12. doi: 10.3343/alm.2012.32.2.105 PMC3289774 22389876

[239] Fink HA Litwack-Harrison S Taylor BC Bauer DC Orwoll ES Lee CG. Clinical Utility of Routine Laboratory Testing to Identify Possible Secondary Causes in Older Men With Osteoporosis: The Osteoporotic Fractures in Men (MrOS) Study. Osteoporos Int (2016) 27(1):331–8. doi: 10.1007/s00198-015-3356-y PMC4719570 26458388

[240] Zang L Shimada Y Nishimura Y Tanaka T Nishimura N Novel A. Reliable Method for Repeated Blood Collection From Aquarium Fish. Zebrafish (2013) 10(3):425–32. doi: 10.1089/zeb.2012.0862 23668933

[241] Holick MF. The Vitamin D Deficiency Pandemic: Approaches for Diagnosis, Treatment and Prevention. Rev Endocrine Metab Disord (2017) 18:153–65. doi: 10.1007/s11154-017-9424-1 28516265

[242] Reid IR. Vitamin D Effect on Bone Mineral Density and Fractures. Endocrinol Metab Clinics North America (2017) 46:935–45. doi: 10.1016/j.ecl.2017.07.005 29080644

[243] Bischoff-Ferrari HA. Influence of Vitamin D on Fracture Reduction Among Older Adults: A Discussion of Recent Meta-Analysis Findings. Osteologie (2019) 28(2):136–9. doi: 10.1055/a-0861-2813

[244] Munns CF Shaw N Kiely M Specker BL Thacher TD Ozono K. Global Consensus Recommendations on Prevention and Management of Nutritional Rickets. J Clin Endocrinol Metab (2016) 101(2):394–415. doi: 10.1210/jc.2015-2175 26745253 PMC4880117

[245] Cherniack EP Troen BR. Calciotropic Hormones. In: Osteoporosis in Older Persons: Advances in Pathophysiology and Therapeutic Approaches, 2nd ed. Springer International Publishing (2016). p. 43–58.

[246] Tiosano D Hochberg Z. Hypophosphatemia: The Common Denominator of All Rickets. J Bone Mineral Metab (2009) 27:392–401. doi: 10.1007/s00774-009-0079-1 19504043

[247] Cavalier E Souberbielle JC. Vitamin D and Its Metabolites: From Now and Beyond. EJIFCC (2018) 29(2):105–10. PMC6053816 30050393

[248] Uday S Högler W. Spot the Silent Sufferers: A Call for Clinical Diagnostic Criteria for Solar and Nutritional Osteomalacia. J Steroid Biochem Mol Biol (2019) 188:141–6. doi: 10.1016/j.jsbmb.2019.01.004 30654108

[249] Seldeen KL Pang M Rodríguez-Gonzalez M Hernandez M Sheridan Z Yu P. A Mouse Model of Vitamin D Insufficiency: Is There a Relationship Between 25(OH) Vitamin D Levels and Obesity? Nutr Metab (2017) 14(1):26. doi: 10.1186/s12986-017-0174-6 PMC5346213 28293271

[250] Szulc P Naylor K Hoyle NR Eastell R Leary ET. Use of CTX-I and PINP as Bone Turnover Markers: National Bone Health Alliance Recommendations to Standardize Sample Handling and Patient Preparation to Reduce Pre-Analytical Variability. Osteoporos Int (2017) 28(9):2541–56. doi: 10.1007/s00198-017-4082-4 28631236

[251] Shaw N Högler W. Biochemical Markers of Bone Metabolism. In: Glorieux FH Pettifor JM Jüppner H. Pediatric Bone. Elsevier Inc (2012). p. 361–81. doi: 10.1016/B978-0-12-382040-2.10015-2

[252] Guañabens N Parés A Alvarez L De Osaba MJM Monegal A Peris P. Collagen-Related Markers of Bone Turnover Reflect the Severity of Liver Fibrosis in Patients With Primary Biliary Cirrhosis. J Bone Miner Res (1998) 13(4):731–8. doi: 10.1359/jbmr.1998.13.4.731 9580479

[253] Delmas PD Demiaux B Malaval L Chapuy MC Edouard C Meunier PJ. Serum Bone Gamma Carboxyglutamic Acid-Containing Protein in Primary Hyperparathyroidism and in Malignant Hypercalcemia. Comparison With Bone Histomorphometry. J Clin Invest (1986) 77(3):985–91. doi: 10.1172/JCI112400 PMC423499 3485113

[254] Uebelhart D Gineyts E Chapuy M-C Delmas PD. Urinary Excretion of Pyridinium Crosslinks: A New Marker of Bone Resorption in Metabolic Bone Disease. Bone Miner (1990) 8(1):87–96. doi: 10.1016/0169-6009(91)90143-N 2106358

[255] Eisman JA Bone HG Hosking DJ McClung MR Reid IR Rizzoli R. Odanacatib in the Treatment of Postmenopausal Women With Low Bone Mineral Density: Three-Year Continued Therapy and Resolution of Effect. J Bone Miner Res (2011) 26(2):242–51. doi: 10.1002/jbmr.212 20740685

[256] Imel EA Liu Z Acton D Coffman M Gebregziabher N Tong Y. Interferon Gamma-1b Does Not Increase Markers of Bone Resorption in Autosomal Dominant Osteopetrosis. J Bone Miner Res (2019) 34(8):1436–45. doi: 10.1002/jbmr.3715 PMC6697186 30889272

[257] Vasikaran S Cooper C Eastell R Griesmacher A Morris HA Trenti T. International Osteoporosis Foundation and International Federation of Clinical Chemistry and Laboratory Medicine Position on Bone Marker Standards in Osteoporosis. Clin Chem Lab Med (2011) 49(8):1271–4. doi: 10.1515/CCLM.2011.602 21605012

[258] Szulc P Delmas PD. Biochemical Markers of Bone Turnover in Men. Calciied Tissue Int (2001) 69(4):229–34. doi: 10.1007/s00223-001-1059-1 11730257

[259] Naylor KE Jacques RM Paggiosi M Gossiel F Peel NFA McCloskey EV. Response of Bone Turnover Markers to Three Oral Bisphosphonate Therapies in Postmenopausal Osteoporosis: The TRIO Study. Osteoporos Int (2016) 27(1):21–31. doi: 10.1007/s00198-015-3145-7 25990354

[260] Stepan JJ. Prediction of Bone Loss in Postmenopausal Women. Osteoporosis Int (2000) 11:S45–54. doi: 10.1007/s001980070005 11193239

[261] Vilaca T Gossiel F Eastell R. Bone Turnover Markers: Use in Fracture Prediction. J Clin Densitom (2017) 20(3):346–52. doi: 10.1016/j.jocd.2017.06.020 28716498

[262] Eastell R Pigott T Gossiel F Naylor KE Walsh JS APeel NF. Bone Turnover Markers: Are They Clinically Useful? Eur J Endocrinol (2018) 178:R19–31. doi: 10.1530/EJE-17-0585 29046326

[263] A Practical Approach to Adolescent Bone Health. A Practical Approach to Adolescent Bone Health. Springer International Publishing (2018). doi: 10.1007/978-3-319-72880-3

[264] Redmond J Fulford AJ Jarjou L Zhou B Prentice A Schoenmakers I. Diurnal Rhythms of Bone Turnover Markers in Three Ethnic Groups. J Clin Endocrinol Metab (2016) 101(8):3222–30. doi: 10.1210/jc.2016-1183 PMC4971334 27294326

[265] Christgau S Bitsch-Jensen O Hanover Bjarnason N Gamwell Henriksen E Qvist P Alexandersen P. Serum CrossLaps for Monitoring the Response in Individuals Undergoing Antiresorptive Therapy. Bone (2000) 26(5):505–11. doi: 10.1016/S8756-3282(00)00248-9 10773591

[266] Rauchenzauner M Schmid A Heinz-Erian P Kapelari K Falkensammer G Griesmacher A. Sex- and Age-Specific Reference Curves for Serum Markers of Bone Turnover in Healthy Children From 2 Months to 18 Years. J Clin Endocrinol Metab (2007) 92(2):443–9. doi: 10.1210/jc.2006-1706 17105843

[267] Choi JI Cho HH. Effects of Di(2-Ethylhexyl)Phthalate on Bone Metabolism in Ovariectomized Mice. J Bone Metab (2019) 26(3):169–77. doi: 10.11005/jbm.2019.26.3.169 PMC6746662 31555614

[268] Kim TH Kim HJ Lee SH Kim SY. Potent Inhibitory Effect of Foeniculum Vulgare Miller Extract on Osteoclast Differentiation and Ovariectomy-Induced Bone Loss. Int J Mol Med (2012) 29(6):1053–9. doi: 10.3892/ijmm.2012.950 22447109

[269] Hammond CL Moro E. Using Transgenic Reporters to Visualize Bone and Cartilage Signaling During Development In Vivo. Front Endocrinol (Lausanne) (2012) 3(JUL). doi: 10.3389/fendo.2012.00091 PMC3399225 22826703

[270] Baird DA Evans DMDS Kamanu FK Gregory JS Saunders FR Giuraniuc CV. Identification of Novel Loci Associated With Hip Shape: A Meta-Analysis of Genomewide Association Studies. J Bone Miner Res (2019) 34(2):241–51. doi: 10.1002/jbmr.3605 PMC6375741 30320955

[271] Yi L Guo X Sun L Hou B. Structural and Functional Sensing of Bio-Tissues Based on Compressive Sensing Spectral Domain Optical Coherence Tomography. Sensors (2019) 19(19):4208. doi: 10.3390/s19194208 PMC6807266 31569799

[272] Du J Bydder GM. Qualitative and Quantitative Ultrashort-TE MRI of Cortical Bone. NMR Biomedicine (2013) 26:489–506. doi: 10.1002/nbm.2906 PMC4206448 23280581

[273] Rad HS Lam SCB Magland JF Ong H Li C Song HK. Quantifying Cortical Bone Water *In Vivo* by Three-Dimensional Ultra-Short Echo-Time MRI. NMR Biomed (2011) 24(7):855–64. doi: 10.1002/nbm.1631 PMC3684973 21274960

[274] Klinck RJ Campbell GM Boyd SK. Radiation Effects on Bone Architecture in Mice and Rats Resulting From *In Vivo* Micro-Computed Tomography Scanning. Med Eng Phys (2008) 30(7):888–95. doi: 10.1016/j.medengphy.2007.11.004 18249025

[275] Solsona CM Sasser T Salmon P Gsell W Viertl D Massey JC. Low-Dose Imaging in a New Preclinical Total-Body PET/CT Scanner. Front Med (2019) 6(APR). doi: 10.3389/fmed.2019.00088 PMC6509903 31131277

[276] Ferizi U Honig S Chang G. Artificial Intelligence, Osteoporosis and Fragility Fractures. Curr Opin Rheumatol (2019) 31:368–75. doi: 10.1097/BOR.0000000000000607 PMC7282383 31045948

[277] Olczak J Fahlberg N Maki A Razavian AS Jilert A Stark A. Artificial Intelligence for Analyzing Orthopedic Trauma Radiographs: Deep Learning Algorithms—are They on Par With Humans for Diagnosing Fractures? Acta Orthop (2017) 88(6):581–6. doi: 10.1080/17453674.2017.1344459 PMC5694800 28681679

[278] Letarouilly JG Broux O Clabaut A. New Insights Into the Epigenetics of Osteoporosis. Genomics (2019) 111:793–8. doi: 10.1016/j.ygeno.2018.05.001 29730394

[279] Astleford K Campbell E Norton A Mansky KC. Epigenetic Regulators Involved in Osteoclast Differentiation. Int J Mol Sci (2020) 21:1–15. doi: 10.3390/ijms21197080 PMC7583862 32992908

[280] Martinez-Moreno JM Fontecha-Barriuso M Martin-Sanchez D Guerrero-Mauvecin J Goma-Garces E Fernandez-Fernandez B. Epigenetic Modifiers as Potential Therapeutic Targets in Diabetic Kidney Disease. Int J Mol Sci (2020) 21:1–26. doi: 10.3390/ijms21114113 PMC7312774 32526941

[281] Kwon DH Ryu J Kim YK Kook H. Roles of Histone Acetylation Modifiers and Other Epigenetic Regulators in Vascular Calcification. Int J Mol Sci (2020) 21(9):3246. doi: 10.3390/ijms21093246 PMC7247359 32375326

[282] Lin W Li Y Chen F Yin S Liu Z Cao W. Klotho Preservation *via* Histone Deacetylase Inhibition Attenuates Chronic Kidney Disease-Associated Bone Injury in Mice. Sci Rep (2017) 7(1):46195. doi: 10.1038/srep46195 28387374 PMC5384196

[283] Silva AM Moura SR Teixeira JH Barbosa MA Santos SG Almeida MI. Long Noncoding RNAs: A Missing Link in Osteoporosis. Bone Res (2019) 7:10. doi: 10.1038/s41413-019-0048-9 30937214 PMC6437190

[284] Srinivasan S Duval MX Kaimal V Cuff C Clarke SH. Assessment of Methods for Serum Extracellular Vesicle Small RNA Sequencing to Support Biomarker Development. J Extracell Vesicles (2019) 8(1):1684425. doi: 10.1080/20013078.2019.1684425 31741724 PMC6844434

[285] Ge XQ Lin H. Noncoding RNAs in the Regulation of DNA Replication. Trends Biochem Sci (2014) 39:341–3. doi: 10.1016/j.tibs.2014.06.003 PMC4265214 25027733

[286] Beroual W Brilli M Biondi EG. Non-Coding RNAs Potentially Controlling Cell Cycle in the Model Caulobacter Crescentus: A Bioinformatic Approach. Front Genet (2018) 9:164. doi: 10.3389/fgene.2018.00164 29899753 PMC5988900

[287] Pircher A Gebetsberger J Polacek N. Ribosome-Associated ncRNAs: An Emerging Class of Translation Regulators. RNA Biol (2014) 11(11):1335–9. doi: 10.1080/15476286.2014.996459 PMC4615380 25692232

[288] Will CL Lührmann R. Spliceosome Structure and Function. Cold Spring Harb Perspect Biol (2011) 3(7):1–2. doi: 10.1101/cshperspect.a003707 PMC3119917 21441581

[289] Dykes IM Emanueli C. Transcriptional and Post-Transcriptional Gene Regulation by Long Non-Coding RNA. Genomics Proteomics Bioinf (2017) 15:177–86. doi: 10.1016/j.gpb.2016.12.005 PMC5487525 28529100

[290] Foessl I Kotzbeck P Obermayer-Pietsch B. miRNAs as Novel Biomarkers for Bone Related Diseases. J Lab Precis Med (2019) 4:2–2. doi: 10.21037/jlpm.2018.12.06

[291] Yavropoulou M Anastasilakis A Makras P Grammatiki M Kotsa K Yovos J. Circulating microRNAs in Postmenopausal Women With Osteoporosis and Vertebral Fractures. Bone Abstr (2016) 5:245. doi: 10.1530/boneabs.5.P245

[292] Hassan MQ Tye CE Stein GS Lian JB. Non-Coding RNAs: Epigenetic Regulators of Bone Development and Homeostasis. Bone (2015) 81:746–56. doi: 10.1016/j.bone.2015.05.026 PMC6095476 26039869

[293] Hackl M Heilmeier U Weilner S Grillari J. Circulating microRNAs as Novel Biomarkers for Bone Diseases – Complex Signatures for Multifactorial Diseases? Mol Cell Endocrinol (2016) 432:83–95. doi: 10.1016/j.mce.2015.10.015 26525415

[294] Lee YR Kim G Tak WY Jang SY Kweon YO Park JG. Circulating Exosomal Noncoding RNAs as Prognostic Biomarkers in Human Hepatocellular Carcinoma. Int J Cancer (2019) 144(6):1444–52. doi: 10.1002/ijc.31931 30338850

[295] Lian JB Stein GS van Wijnen AJ Stein JL Hassan MQ Gaur T. MicroRNA Control of Bone Formation and Homeostasis. Nat Rev Endocrinol (2012) 8(4):212–27. doi: 10.1038/nrendo.2011.234 PMC3589914 22290358

[296] Masuda T Mori A Ito S Ohtsuki S. Quantitative and Targeted Proteomics-Based Identification and Validation of Drug Efficacy Biomarkers. Drug Metab (2021) 36:100361. doi: 10.1016/j.dmpk.2020.09.006 33097418

[297] Lee JH Cho JY. Proteomics Approaches for the Studies of Bone Metabolism. BMB Rep (2014) 47(3):141–8. doi: 10.5483/BMBRep.2014.47.3.270 PMC4163882 24499667

[298] Nielson CM Jacobs JM Orwoll ES. Proteomic Studies of Bone and Skeletal Health Outcomes. Bone (2019) 126:18–26. doi: 10.1016/j.bone.2019.03.032 30954730 PMC7302501

[299] Calciolari E Donos N. Proteomic and Transcriptomic Approaches for Studying Bone Regeneration in Health and Systemically Compromised Conditions. Proteomics – Clin Appl (2020) 14(3):1900084. doi: 10.1002/prca.201900084 32131137

[300] Yang TL Shen H Liu A Dong SS Zhang L Deng FY. A Road Map for Understanding Molecular and Genetic Determinants of Osteoporosis. Nat Rev Endocrinol (2020) 16:91–103. doi: 10.1038/s41574-019-0282-7 31792439 PMC6980376

[301] Ohlsson C Sjögren K. Effects of the Gut Microbiota on Bone Mass. Trends Endocrinol Metab (2015) 26:69–74. doi: 10.1016/j.tem.2014.11.004 25497348

[302] Medina-Gomez C. Bone and the Gut Microbiome: A New Dimension. J Lab Precis Med (2018) 3:96–6. doi: 10.21037/jlpm.2018.11.03

[303] Ohlsson C Sjögren K. Osteomicrobiology: A New Cross-Disciplinary Research Field. Calciied Tissue Int (2018) 102:426–32. doi: 10.1007/s00223-017-0336-6 PMC5851705 29079994

[304] Guss JD Horsfield MW Fontenele FF Sandoval TN Luna M Apoorva F. Alterations to the Gut Microbiome Impair Bone Strength and Tissue Material Properties. J Bone Miner Res (2017) 32(6):1343–53. doi: 10.1002/jbmr.3114 PMC5466506 28244143

[305] Jansson PA Curiac D Lazou Ahrén I Hansson F Martinsson Niskanen T Sjögren K. Probiotic Treatment Using a Mix of Three Lactobacillus Strains for Lumbar Spine Bone Loss in Postmenopausal Women: A Randomised, Double-Blind, Placebo-Controlled, Multicentre Trial. Lancet Rheumatol (2019) 1(3):e154–62. doi: 10.1016/S2665-9913(19)30068-2 38229392

[306] Nilsson AG Sundh D Bäckhed F Lorentzon M. *Lactobacillus Reuteri* Reduces Bone Loss in Older Women With Low Bone Mineral Density: A Randomized, Placebo-Controlled, Double-Blind, Clinical Trial. J Intern Med (2018) 284(3):307–17. doi: 10.1111/joim.12805 29926979

[307] Heale R Forbes D. Understanding Triangulation in Research. Evidence-Based Nursing (2013) 16:98. doi: 10.1136/eb-2013-101494 23943076

